# Supplementary figures and images for: Modeling the Putative Ancient Distribution of Aedes togoi (Diptera: Culicidae)
Source: J Insect Sci. 2020 May 26;20(3):7. doi: 10.1093/jisesa/ieaa035 (PMC7248266; doi:10.1093/jisesa/ieaa035)

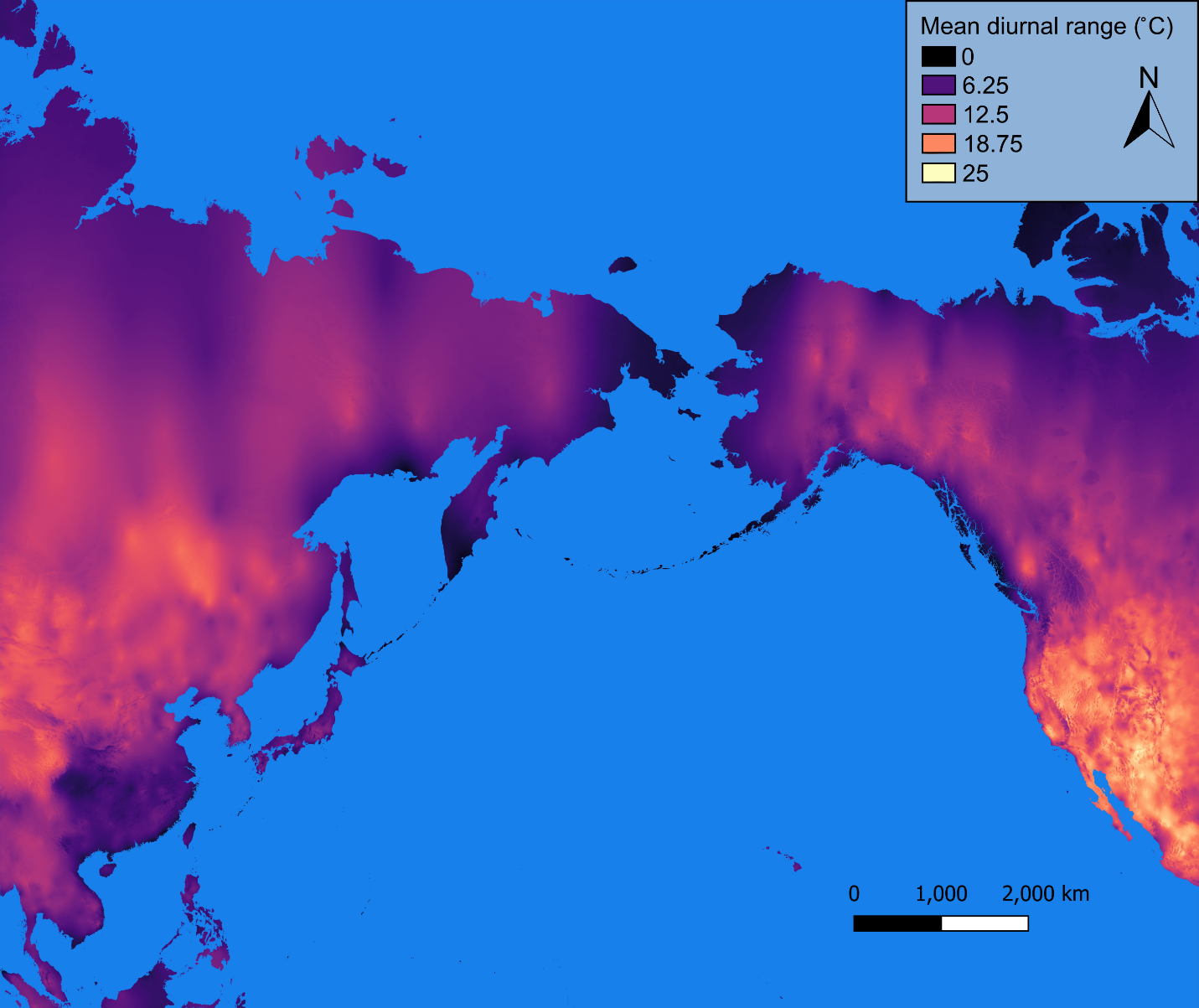


**Supplementary Figure 8.** Mean diurnal temperature range (BIO 02) under current climate conditions.

Supplement: ieaa035_suppl_Supplementary_Figure_1 [file ieaa035_suppl_supplementary_figure_1.docx]

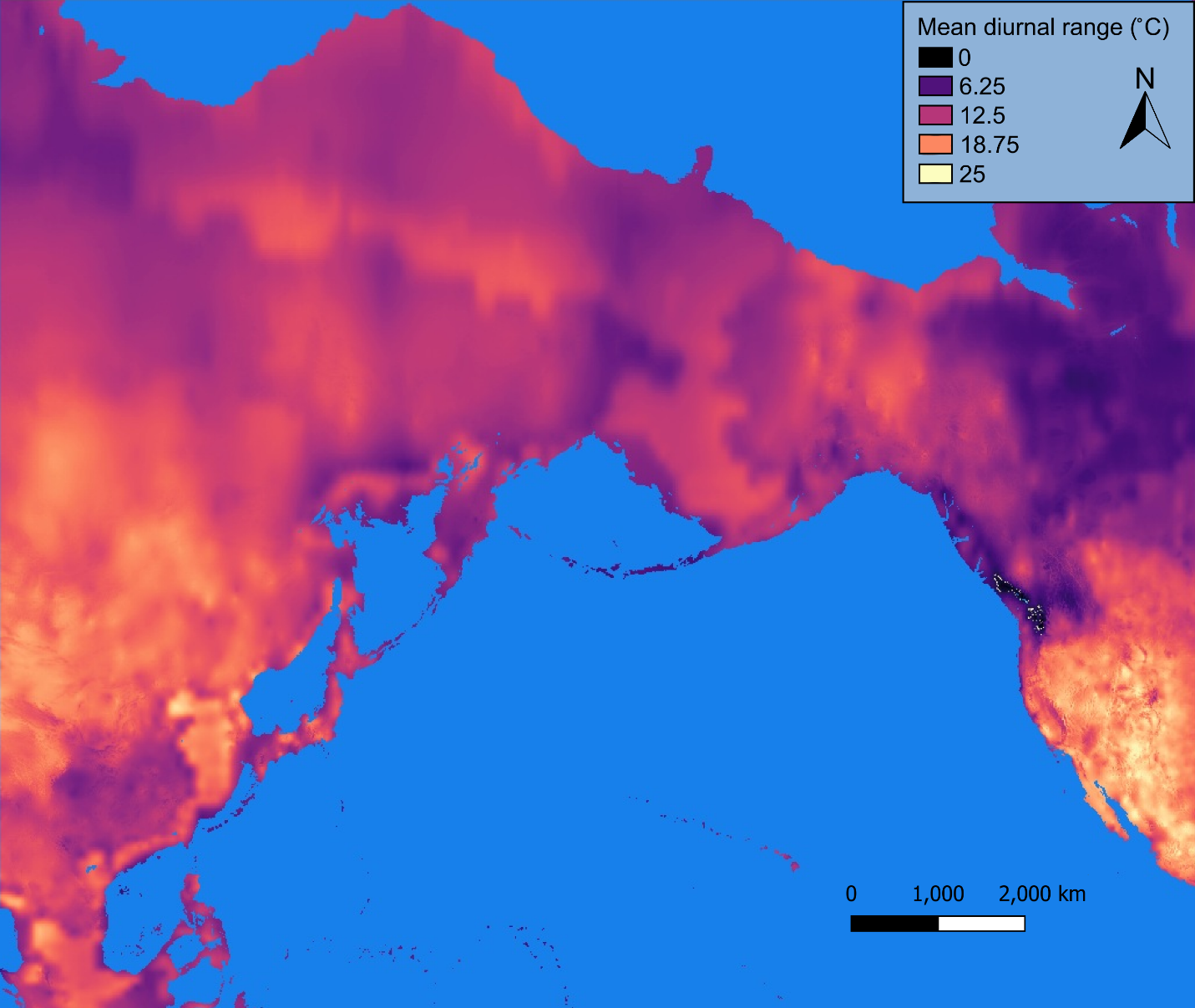
**Supplementary Figure 10.** Mean diurnal temperature range (BIO 02) during the last glacial maximum.

Supplement: ieaa035_suppl_Supplementary_Figure_3 [file ieaa035_suppl_supplementary_figure_3.docx]

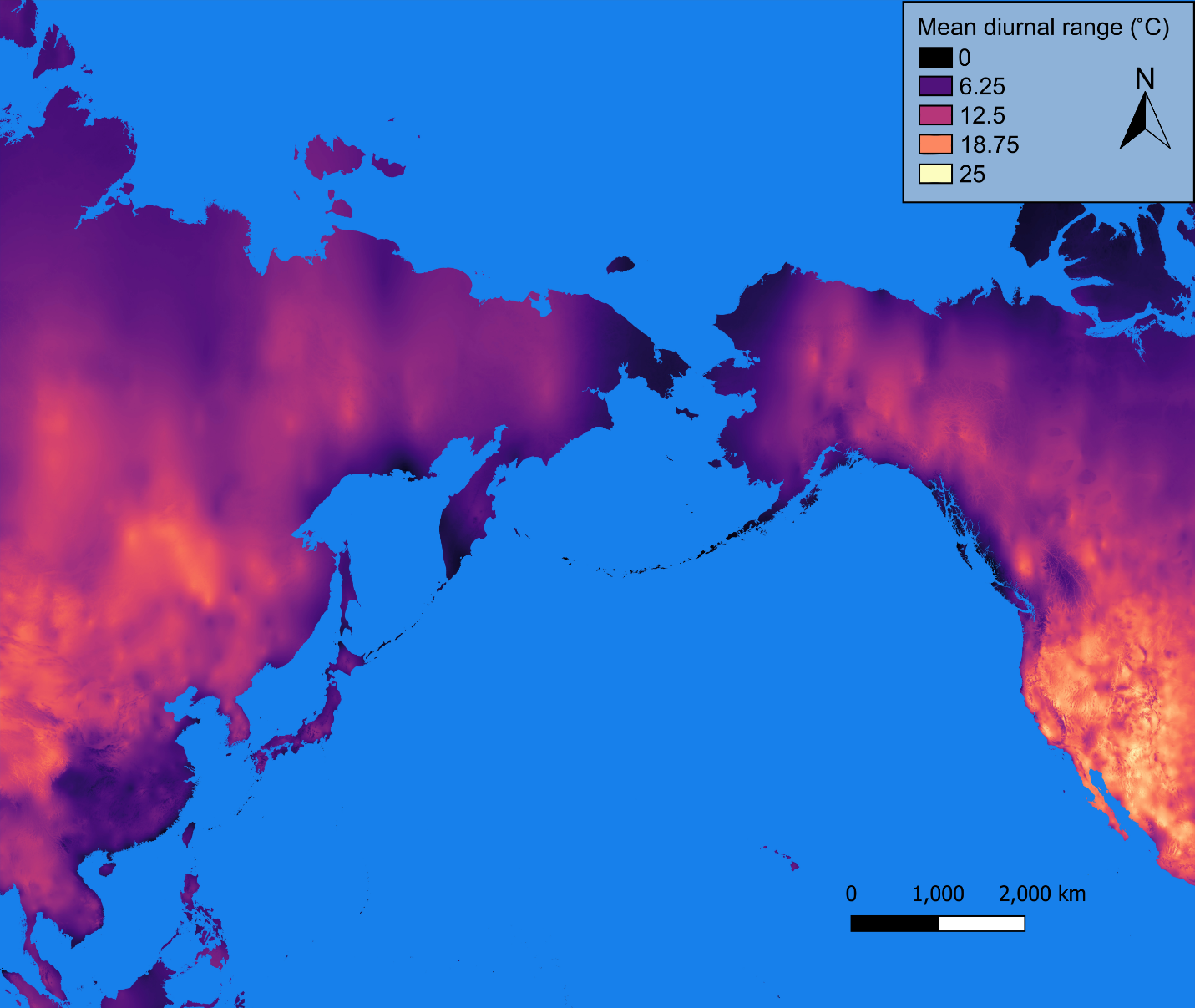
**Supplementary Figure 11.** Mean diurnal temperature range (BIO 02) during the mid-Holocene period.

Supplement: ieaa035_suppl_Supplementary_Figure_4 [file ieaa035_suppl_supplementary_figure_4.docx]
