# Supplementary material for: Modeling the Putative Ancient Distribution of Aedes togoi (Diptera: Culicidae)
Source: J Insect Sci. 2020 May 26;20(3):7. doi: 10.1093/jisesa/ieaa035 (PMC7248266; doi:10.1093/jisesa/ieaa035)
Supplement: ieaa035_suppl_Supplementary_Figure_14 [file ieaa035_suppl_supplementary_figure_14.docx]

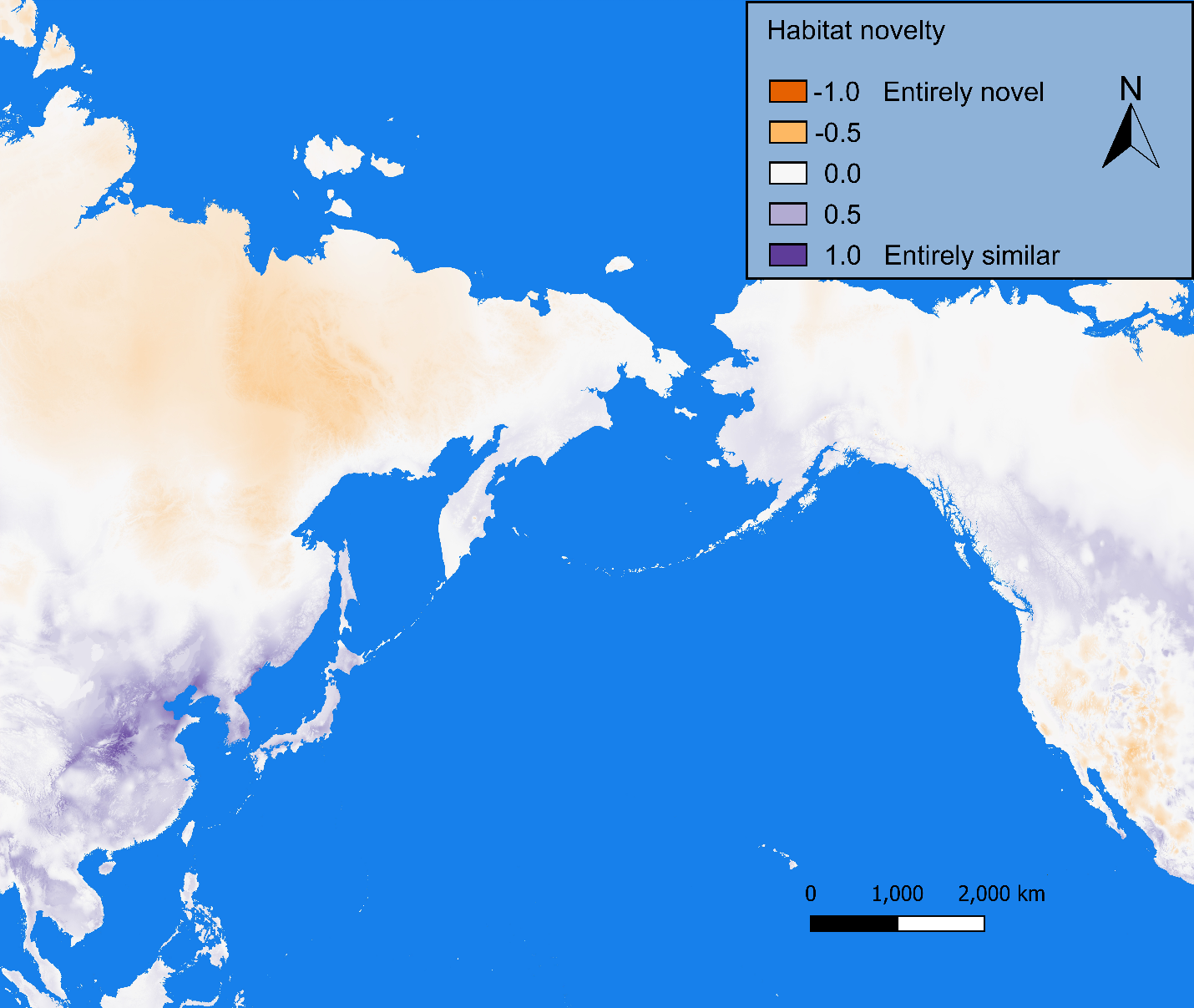


**Supplementary Figure 5.** A multidimensional environmental similarity surface (MESS) showing where novel climate conditions exist in the projection layers of the mid-Holocene period based on model 1.
