# Supplementary material for: Modeling the Putative Ancient Distribution of Aedes togoi (Diptera: Culicidae)
Source: J Insect Sci. 2020 May 26;20(3):7. doi: 10.1093/jisesa/ieaa035 (PMC7248266; doi:10.1093/jisesa/ieaa035)
Supplement: ieaa035_suppl_Supplementary_Files [file ieaa035_suppl_supplementary_files.zip › Peach revised version.pdf]

OXFORD UNIVERSITY PRESS | Journal of Insect Science

Modelling the Putative Ancient Distribution of the Coastal Rock Pool Mosquito *Aedes togoi*

Journal:	*Journal of Insect Science*
Manuscript ID	JIS-2020-0009.R1
Manuscript Type:	Research
Date Submitted by the Author:	07-Apr-2020
Complete List of Authors:	Peach, Daniel; Simon Fraser University, Biological Sciences<br>Matthews, Benjamin; The University of British Columbia, Zoology
Organism Keywords:	Mosquitoes, Culicidae, Aedes
Field Keywords:	Biogeography, Paleobiology, Invasive Species

SCHOLARONE™
Manuscripts

Modelling the Putative Ancient Distribution of the Coastal Rock Pool Mosquito *Aedes togoi*

Daniel A H Peach (ORCID 0000-0002-8913-5133)[1], Benjamin J Matthews (ORCID 0000-0002-8697-699X)[1]

[1] Department of Zoology, University of British Columbia, Vancouver, BC Canada

Correspondence to: dan@danpeach.net and ben.matthews@zoology.ubc.ca

Abstract

The coastal rock pool mosquito, *Aedes togoi*, is found in coastal east Asia in climates ranging from subtropical to subarctic. However, a disjunct population in the Pacific Northwest of North America has an ambiguous heritage. Two potential models explain the presence of *Ae. togoi* in North America: ancient Beringian dispersal or modern anthropogenic introduction. Genetic studies have thus far proved inconclusive. Here we described the putative ancient distribution of *Ae. togoi* habitat in east Asia and examined the climatic feasibility of a Beringian introduction into North America using modern distribution records and ecological niche modeling of bioclimatic data from the last interglacial period (~120,000 BP), the last glacial maximum (~21,000 BP), and the mid-Holocene (~6000 BP). Our results suggest that suitable climatic conditions existed for *Ae. togoi* to arrive in North America through natural dispersal as well as to persist there until present times. Furthermore, we find that ancient distributions of suitable *Ae. togoi* habitat in east Asia may explain the genetic relationships between *Ae. togoi* populations identified in other studies. These findings indicate the utility of ecological niche modeling as a complementary tool for studying insect phylogeography.

Introduction

The coastal rock pool mosquito, *Aedes* (*Tanakius*) *togoi* (Theobald), breeds in pools of brackish or salt-water above the high tide level on rocky shorelines and, sporadically, in containers of freshwater further inland (Petrishcheva 1948, Tanaka et al. 1979). *Ae. togoi* is a vector of the filarial parasite *Brugia malayi* (Tanaka et al. 1979, Hayashi 2011, Wada 2011), Japanese encephalitis (Petrishcheva 1948, Rosen et al. 1978, Rosen 1986) and potentially *Wuchereria bancrofti* and *Dirofilaria immitis* (Tanaka et al. 1979). *Ae. togoi* is found in coastal areas of east Asia, from subtropical to subarctic environments (Petrishcheva 1948, Tanaka et al. 1979, Sota et al. 2015), while a peripheral population of *Ae. togoi* also exists in the Pacific Northwest of North America along the coast of southern British Columbia, Canada (BC) and northern Washington, USA (Belton 1983, Darsie and Ward 2005, Peach 2018, Peach et al. 2019). The provenance of this population is unknown (Sota et al. 2015, Peach 2018), and two alternative hypotheses have been proposed to explain the presence of *Ae. togoi* in North America: invasion via anthropogenic dispersal and arrival via natural Beringian dispersal.

A trans-Beringian distribution is found in several insect species (Kavanaugh 1988), and many species of mosquito are Holarctic in distribution (Wood et al. 1979, Becker et al. 2010), but the restricted coastal habitat used by *Ae. togoi* may have limited it to the Pacific Rim. There are several reasons that the full distribution of *Ae. togoi* is not known. The remoteness and ruggedness of the region and Cold War politics have likely contributed to a dearth of sampling north of Japan. In North America, *Ae. togoi* do not tend to travel more than 20m from the shoreline (Trimble 1984) and thus trapping efforts outside of this narrow zone will not collect them. The rocky pools used by *Ae. togoi* is atypical mosquito habitat and is often difficult to access. Furthermore, larvae can remain submerged in detritus at the bottom of pools for extended

periods of time (Sames et al. 2004). North American *Ae. togoi* have not been found breeding in containers (Trimble 1984, Sames et al. 2004), a common life-history trait of invasive mosquitoes (Hawley et al. 1987, Lounibos 2002). Conversely, there is some evidence for anthropogenic dispersal of *Ae. togoi* in parts of Asia (Sota et al. 2015) where it has been found on ships travelling between islands (Bohart 1956) and breeding in artifical containers (Tanaka et al. 1979) and the bilges of ships (Hsiao and Bohart 1946). One report suggests that certain populations of *Ae. togoi* in the Russian Far East can reside inland in freshwater containers associated with human habitation (Petrishcheva 1948). Furthermore, when suitable habitat is targeted for mosquito surveys *Ae. togoi* is regularly found (Sames et al. 2004), and it has been observed flying over tidewater to bite the occupants of boats at anchor in North America (Belton and Belton 1989).

Ae. togoi was initially described in Asia in 1907 (Theobald 1907) and first reported in North America from Victoria, BC in 1970 (Sollers-Riedel 1971). However, an undated specimen identified in the Canadian National Collection of Insect, Arachnids, and Nematodes in 1974 may have been collected as early as the 1940's (Belton 1980). Furthermore, a 1919 record of *Aedes* (*Ochlerotatus*) *dorsalis* larvae from the supralittoral coastal rock pools at Caulfield Cove in North Vancouver, BC (Hearle 1926) has been proposed to be a mis-identification of *Ae. togoi* (Trimble and Wellington 1979). *Ae. dorsalis* is a floodwater mosquito that breeds in grassy, brackish tidal marshes (Wood et al. 1979, Belton 1983) while the permanent coastal rock pools at Caulfield Cove, at least currently (as of December 2019), host *Ae. togoi* and *Culiseta incidens* larvae (DP, Pers. Obs.). Thus, it is possible that *Ae. togoi* could have gone unnoticed in North America until sometime in the early-to-mid 20th century.

Genetic evidence based on mitochondrial sequencing suggests that there are at least 4 distinct lineages of *Ae. togoi*: a lineage from temperate and subarctic regions of Japan, China, Taiwan, and other parts of Southeast Asia, a lineage from the subtropical islands of Japan, a lineage from subarctic Japan, and a lineage from Canada (Sota et al. 2015). If the *Ae. togoi* lineage in North America had arisen from natural divergence it is estimated to have diverged since some time in the Paleolithic era (Sota et al. 2015). This estimated divergence time spans several glacial cycles during which Beringian dispersal may have been possible. Alternatively, there may be unknown populations of *Ae. togoi* from which the North American population is derived (Sota et al. 2015). In addition to solving an entomological mystery, accurate accounting of *Ae. togoi* as invasive or indigenous has important implications. A change in status could alter *Ae. togoi*'s inclusion in analyses of invasion ecology/biology and help refine analyses of the attributes possessed by invasive mosquitoes. It could also properly inform legislative and other future action to combat invasive species and influence vector monitoring and control efforts. For example, we may not have to worry about the northward spread of *Ae. togoi* under climate change scenarios (e.g. Peach et al. 2019) if it is already indigenous to such areas.

Species distribution modeling, a type of environmental niche modeling, is a useful approach to estimate the suitability of habitat for a species in geographic areas it is not known to occupy or to estimate the changes in suitability of habitat for a species with environmental change over time (Wiens et al. 2009, Warren and Seifert 2011, Herrando-Moraira et al. 2019). Maximum entropy niche modelling (Maxent) is a commonly used approach in species distribution modelling (Rochlin et al. 2013, Melaun et al. 2015, Cunze et al. 2016, Wang et al. 2017, Peach et al. 2019, Rochlin 2019). Maxent is an effective open-source machine-learning algorithm that uses presence-only data to model habitat suitability (Elith et al. 2006, Phillips et

al. 2006, 2017, Merow et al. 2013, West et al. 2016, Ashraf et al. 2017) and is frequently used to predict shifts in distribution of suitable habitat due to climate change (Rochlin et al. 2013, Wang et al. 2017, Peach et al. 2019) and putative distributions for species that become invasive in new regions (West et al. 2016, Peach et al. 2019, Rochlin 2019). Maxent modelling has also be applied to predict suitable habitat for a species under past climate conditions (Ye et al. 2014), including the paleodistribution of mosquitoes (Porretta et al. 2012). In the present study, we use available climate data to model the suitability of habitat for *Ae. togoi* along the Pacific Rim over the most recent glacial cycle to determine if suitable conditions existed for *Ae. togoi* to naturally disperse into and survive in North America. Moreover, we estimate the ancient distribution patterns of this mosquito in Asia and identify potential areas of allopatry that might be responsible for the distinct lineages found by Sota et al. (2015), as well as predict the current distributions of suitable *Ae. togoi* habitat that will help focus the search for as-yet unknown populations.

Materials and methods

Occurrence data

In the present study, we compiled 86 Asian records and 49 North American records of *Ae. togoi* within the study area (Figure 1, Supplementary Table 1). These records were taken from a combination of published literature (Petrishcheva 1948, Bohart 1956, Shestakov 1961, Lien 1962, Omori 1962, Kim and Seo 1968, Ramalingam 1969, Tanaka et al. 1975, 1979, Belton 1980, Sazonova and Smirnova 1986, Belton and Belton 1989, Sota 1994, Lee and Hong 1995, Sames et al. 2004, Stephen et al. 2006, Cheun et al. 2011, Sota et al. 2015, Petersen et al. 2017),

personal collection records and observations (Gowlland Harbour, BC, Malcom Island, BC, Ucluelet, BC, Port Renfrew, BC, West Vancouver, BC, and Victoria, BC; DP, pers. obs.), and from museum specimens in the Beaty Biodiversity Museum in Vancouver, BC and the Royal British Columbia Museum in Victoria, BC. When identifiable place names were given rather than latitude and longitude, we derived associated coordinates from the centre of the location using Google Earth software (http://www.google.com/earth/download/ge). Some data points did not occur on pixels within bioclimatic data due to the irregular shape of coastline or the small size of islands. When this occurred, we relocated data points to the closest 1 km^2 raster cell.

Study area

We selected two study areas: one from Asia from within Latitude 0°N to 49°N and Longitude 94°E to 157°E (boundaries indicated on Figure 3), roughly encompassing the known distribution of *Ae. togoi* in Asia, and one for the North Pacific from within Latitude 0°N to 80°N and Longitude 95°E across the Pacific to 100°W, encompassing all known *Ae. togoi* populations in Asia and North America and possible unknown populations in the North Pacific. We projected a model from the Asian study area (hereafter referred to as model 1), obtained using Asian *Ae. togoi* populations, onto the North Pacific study area, following methodology for projecting habitat of invasive species into new areas (Cunze et al. 2018). We also generated results for the North Pacific study area using all known *Ae. togoi* populations (hereafter referred to as model 2), as if it were indigenous. We used WGS84 Mercator projection for maps in the last glacial maximum, and WGS84/PDC Mercator projection for all other maps.

Environmental variables

We downloaded 19 bioclimatic variables (Table 1) based on current climatic conditions at a scale of approximately 1 km^2 (Hijmans et al. 2005) from Worldclim (http://worldclim.org – dataset version v1.4, bioclimatic variables at 30s resolution). We also downloaded corresponding sets of variables based on climate conditions from: 1) the last interglacial period (~120,000-140,000 BP) at a scale of 1 km^2 (Otto-Bliesner et al. 2009), 2) the last-glacial maximum (~22,000 BP) at a scale of 5 km^2 (Gent et al. 2011), and 3) mid-Holocene period (~6000 BP) at a scale of 1 km^2 (Gent et al. 2011). We used these datasets to project our models onto past environments. We chose input bioclimatic variables based on possession of a weak intercorrelation with other variables, defined as a Pearson correlation $<|0.8|$ calculated with ENMTools version 1.4.4 (Warren et al. 2010) (Supplementary Table 2, 3). When variables were highly correlated, we prioritized parameters for winter temperature due to relevance for *Ae. togoi* overwintering strategy (Sota 1994) and their importance in previous studies (Peach et al. 2019), parameters for precipitation due to reports of larval habitat drying out (Wada et al. 1993), and parameters for temperature of the wettest quarter due to their importance in previous studies (Peach et al. 2019). We calculated an Akaike information criterion (AICc) using ENMTools version 1.4.4 (Warren et al. 2010) and Maxent version 3.4.1 (Phillips et al. 2018) to compare models created with distinct environmental variables, model settings, and/or regularization parameters (Supplementary Table 4, 5). We retained one best-performing model constructed from only Asian *Ae. togoi* observations (model 1) (Table 2) and all known *Ae. togoi* observations (model 2) (Table 3), each with different sets of 6 variables and clamping applied.

Maxent

Maximum entropy niche modelling (Maxent) is a common approach for species habitat modelling (Melaun et al. 2015, Cunze et al. 2016, Peach et al. 2019, Rochlin 2019). Maxent is a machine-learning algorithm that models putative species distributions using presence-only data (Phillips et al. 2006, 2018). Maxent compares favourably to similar methods (Elith et al. 2006, Phillips et al. 2006, Padalia et al. 2014), even when minimal presence data is available (Elith et al. 2006, 2006). We used Maxent version 3.4.1 (Phillips et al. 2018) to model suitable habitat for *Ae. togoi*. We used default Maxent settings with and without linear features, and with and without quadratic features, with 1000 replications, each with 2000 training iterations, 20% of data points withheld for subsampling, clamping applied, and 10,000 background points. We analyzed results based on extensive presence of predicted suitable habitat on maps (original maps available to download at https://doi.org/10.5683/SP2/YPVTYT) calculated from the maximum sensitivity and specificity, a recommended approach for transforming the results of species distribution models into binary presence/absence predictions (Liu et al. 2005), as well as the area under the curve (AUC_{PO}) of the receiving operator characteristic (ROC). This integral gives a sum between 0 and 1 with a result >0.5 indicative of a model that is more accurate than random, >0.7 representing a useful model, >0.9 representing an excellent model, and a result of 1 indicating a perfect fit (Swets 1988). We compared the AUC_{PO} of our three best candidate modes as measured by AICc and selected the top performer across both metrics (Table 2, 3). We also compared the relative contributions of the variables used by having Maxent randomly perform permutations of the value of each variable on presence and background data and then reassess the model. Decreases in AUC_{PO} were transformed to a percentage and used as a relative metric of variable importance. We built maps with QGIS version 3.4.3 (QGIS Development Team 2018), and downloaded spatial data on the extent of glaciation during the last glacial maximum

(Ehlers et al. 2011) from the Collaborative Research Centre 806 Database (https://crc806db.uni-koeln.de/) to add glacial coverage layers where applicable.

Results

Model Selection and relevant variables

We retained six variables under default Maxent settings without linear features and with a regularization multiplier of 2 for model 1 (Table 2), and six variables under default Maxent settings without linear features and with a regularization multiplier of 1 for model 2 (Table 3). Both models used three shared variables: mean diurnal range, minimum temperature of the coldest month, and mean temperature of the warmest quarter. Model 1 has a mean AUC_{PO} of 0.912 (± 0.031) and model 2 has a mean AUC_{PO} of 0.972 (± 0.009), both representing models with excellent fits (Swets 1988). The maximum sensitivity and specificity threshold of model 1 was 0.316, whereas it was 0.137 for model 2. Model 1 converged after a mean of 892.8 training iterations, and model 2 converged after a mean of 1073 training iterations.

For model 1, mean diurnal range, the minimum temperature of the coldest month, and precipitation of the coldest quarter were the relatively most important permutation variables (Table 4, Figure 2). For model 2, mean diurnal range, the minimum temperature of the coldest month, and mean temperature of the warmest quarter were the relatively most important permutation variables (Table 4, Figure 2). The distribution of novel climate conditions for projections during each period can be found in Supplementary Figures 1-7.

Current Conditions

Under current conditions our models predict possible *Ae. togoi* habitat on the North Coast of British Columbia, the Alexander Archipelago, Cook Inlet, and parts of the Aleutian Islands of Alaska, eastern Kamchatka, the Kurile Islands, the Philippines, and the Hawaiian Islands (Figures 3 & 4). Model 1 shows much more extensive suitable habitat for *Ae. togoi* species around most of the North Pacific, including areas not predicted by model 2 such as Taui Bay in the sea of Okhotsk, the northern Kurile Islands, most of Kamchatka, most of the Aleutian Islands, the Pribolif Islands, the coasts of the Bering Sea, and most of the coast of Alaska (Figure 3).

Last Interglacial Period

Our models both show a reduction of suitable habitat for *Ae. togoi* in mainland Asia during the last interglacial period (Figures 5 & 6); however, in general the predicted habitat for *Ae. togoi* during this time period are roughly similar to those predicted by our models for the current time period. One exception is that our model 2 predicts increased areas of suitable habitat in Kamchatka, the Aleutians, mainland Alaska, the Alexander Archipelago, and coastal British Columbia during the last interglacial period (Figure 6).

Last Glacial Maximum

Our model 1 predicts that ample habitat suitable for *Ae. togoi* was present along the coast of the Bering land bridge, the Aleutian Islands, and the coast of North America from Alaska to the Baja during the last glacial maximum (Figure 7). While much of this area was covered by ice sheets, our model predicts that patches of exposed rock pools along the coast could have provided suitable microhabitats for *Ae. togoi*. Moreover, ice-free glacial refugia such as portions of the Aleutian Islands, the Alexander Archipelago, Haida Gwaii, and northern Vancouver Island

are predicted to be suitable habitat for *Ae. togoi*. Model 1 also suggests regions of suitable habitat for *Ae. togoi* in eastern Asian during this time period including Japan's main islands, the Ryukyu archipelago, the Izu Archipelago, the Bonin Islands coastal China, parts of the Philippines, and along the coast of the South China sea and the islands it contained (Figure 7). Conversely, our model 2 predicts that suitable habitat for *Ae. togoi* during the last glacial maximum was restricted to a small region of coastal California, Baja California, and the Channel Islands of California (Figure 8). Model 2 suggests largely the same distribution of suitable habitat for *Ae. togoi* in eastern Asia during the last glacial maximum as model 1 (Figure 8).

Mid-Holocene

Our models both predict that the distribution of *Ae. togoi* habitat in the mid-Holocene was roughly similar to the present-day distribution of *Ae. togoi* (Figures 9 & 10). Differences include model 1 predicting reduced *Ae. togoi* habitat particularly along the coast of the Gulf of Alaska and the coastlines of the Bering Sea (Figure 9), whereas model 2 predicts reduced habitat in Cook Inlet and the Alexander Archipelago (Figure 10).

Discussion

Our models predict the presence of suitable climate for *Ae. togoi* around much of the North Pacific, including regions of North America and Far Eastern Russia without existing observations of *Ae. togoi*. These areas, including parts of Kamchatka, the Aleutian Islands, Cook Inlet, the Alexander Archipelago, and the north Coast of British Columbia, are remote and rugged terrain that has very likely been under-surveyed. Therefore, in agreement with Sota et al. (2015), we predict that undiscovered *Ae. togoi* populations may exist in the North Pacific.

Model 1, which uses only Asian observations of *Ae. togoi,* shows a much greater predicted distribution of *Ae. togoi* habitat than model 2, which additionally included North American observations. Model 1 and model 2 contain different bioclimatic variables, and as such some differences in the distributions they predict should be expected. However, projecting species habitat suitability into a novel range using only training data from the original range can lead to overestimation of projected suitable habitat due to local or regional differences in the ecological adaptations of sub-populations which, when taken in aggregate, can overestimate the species' ecological breadth (Stockwell and Peterson 2002). Furthermore, *Ae. togoi* almost exclusively uses supralittoral rock pools as breeding habitat and, accordingly, results from both models should be interpreted to mean that only subsets of the environmentally suitable range that contain suitable breeding habitat are potential niches for *Ae. togoi*. Interestingly, both of our models predict suitable conditions for *Ae. togoi* in the Hawaiian Islands, as have previous studies (Peach et al. 2019), as well as other Pacific Islands such as Guam and the Northern Mariana Islands.

Both of our models predict that putative *Ae. togoi* habitat existed along the Pacific coast of North America during the last interglacial period, last glacial maximum, mid-Holocene, and present day. However, our model 2 predicts that suitable *Ae. togoi* habitat during the last glacial maximum was restricted to California, the Channel Islands of California, and Baja California. Whether or not *Ae. togoi* could have followed the shifting distribution of suitable environmental conditions to this area as North America cooled is unknown. While much of northern North America was covered by ice sheets during the last glacial maximum (Shafer et al. 2010, Ehlers et al. 2011), *Ae. togoi* could have persisted in one or more glacial refugia such as the Aleutians, the Alexander Archipelago, Haida Gwaii, northern Vancouver Island, or other cryptic refugia

(Shafer et al. 2010). This continual presence of suitable *Ae. togoi* habitat in North America throughout a complete glacial cycle, along with suitable ancient habitat connecting North America to Asian *Ae. togoi* populations, implies that this species could have naturally dispersed into North America and survived long-term climatic cycles into the present. We note that presence-only models can be inaccurate when produced from data that suffers from sampling bias (Yackulic et al. 2013) that and our models may suffer from limitations in predicting paleodistributions of suitable *Ae. togoi* habitat due to a dearth of sampling records from remote and difficult to access regions in the northern coastal regions of *Ae. togoi's* present range.

The present study suggests that *Ae. togoi* habitat has existed in much of coastal southeast Asia throughout the last glacial cycle and that its distribution may partially explain the relationships between geographically distinct populations described in other studies. The continuous presence of suitable climatic conditions for *Ae. togoi* habitat in the Ryukyu archipelago predicted by both of our models throughout all time frames examined supports, and may partially explain, the unique lineage of *Ae. togoi* populations described on these islands (Sota et al. 2015). The potential for continuous presence of *Ae. togoi* on the Ryukyu Archipelago implied by our models, coupled with a barrier to gene flow, may have allowed for the evolution of an *Ae. togoi* sub-population with specific adaptations to these islands. Widespread and largely connected *Ae. togoi* habitat along the coasts of east and southeast Asia throughout the last glacial cycle may also explain the widespread distribution of the single *Ae. togoi* lineage found from subarctic to subtropical locations (Sota et al. 2015). We believe that the benefit of linking population genetic structure to geography across distinct evolutionary scenarios is a key benefit of including studies of paleodistribution from species distribution modelling techniques in phylogeography (Porretta et al. 2012).

Mean diurnal range and minimum temperature of the coldest month were important environmental variables in each of our models. In addition, precipitation during the coldest quarter was also critical in model 1, while mean temperature during the warmest quarter was important in model 2. There is evidence that, under certain conditions, temperature fluctuations (rather than absolute temperature) can have negative effects on the development rate and survival of some mosquitoes (Lyons et al. 2013). With this in mind, the importance of mean diurnal range may reflect a biological reliance on relatively stable environmental temperature conditions during *Ae. togoi* larval development. Alternatively, the importance of mean diurnal temperature range in our models may simply reflect that *Ae. togoi*'s breeding habitat is close to the ocean and subject to its stabilizing effect on temperature. The minimum temperature of the coldest month is an important environmental variable for the habitat of other *Aedes* spp. (Melaun et al. 2015, Lubinda et al. 2019), and another measure of cold tolerance, the mean temperature of the coldest quarter, was found to be important for *Ae. togoi* habitat suitability in North America (Peach et al. 2019). Such parameters are important determinants of overwintering strategy (Sota 1994) and overwinter survival of some mosquitoes (Kaufman and Fonseca 2014). Insulation of mosquito breeding habitat by snow may help to protect eggs and larvae from the effects of low air temperature (Hanson and Craig 1995) and could be responsible for the importance of precipitation during the coldest quarter as a variable in model 1. *Ae. togoi* breeding habitat does occasionally dry out (Wada et al. 1993), and it could also be that this variable simply reflects the danger of these pools drying out when the mosquitoes are restricted to the egg or larval stage and at their most vulnerable to desiccation. The importance of mean temperature of the warmest month on *Ae. togoi* habitat suitability in model 2 may reflect the well-known importance of temperature in mosquito development times and survival (Alto and Juliano 2001, Reinhold et al.

2018). This variable is also important in for the habitat suitability of other *Aedes* spp. (Cunze et al. 2016). Despite its importance in other studies (Peach et al. 2019), the mean temperature of the wettest quarter was not part of our best-fitting models. This could be due to the different geographic areas used in our models. Despite the different co-variables present in model 1 and model 2, we do not observe drastic differences in response curves for important shared variables. Comparing the response curves (Figure 2) indicates that lower mean diurnal ranges of greater than 12 °C indicate poor habitat suitability in both models. Both models also show that minimum temperatures as low as -30 °C still indicate suitable habitat for *Ae. togoi*, but that habitat suitability drops off quickly with minimum temperatures above 20 °C (though peak habitat suitability has shifted to the right in model 2). Consistent with a niche conservatism hypothesis (Wiens and Graham 2005), this overall similarity implies that the thermal niche of *Ae. togoi* may be fairly constant, whether one examines just the population from Asia or the entire known population. Future studies will be required to further elucidate the niches of different populations (e.g. Asia vs. North America, populations that diapause as eggs vs. those that diapause as larvae).

Trade from Japan has been suggested as a potential vehicle for the introduction of *Ae. togoi* into North America (Belton 1983, Sota et al. 2015), but there was Russian activity related to colonization, the fur trade, whaling, collection and shipping of salt, and other commercial activities on the Pacific Coast of North America as far back as the 18th century (Gibson 1980, Lightfoot 2003). This exchange between Northeast Russia and Pacific North America by the Russian American Company and its predecessors, involving Russian ports in the Sea of Okhotsk, Amur region, and Kamchatka (Gibson 1980), could also have been responsible for the introduction of *Ae. togoi* to North America. Interestingly, the ships used to transport and supply Canadian soldiers and the Royal Northwest Mounted Police, deployed to combat Bolsheviks in

Vladivostok and Siberia as part of the Canadian Siberian Expeditionary Force (Isitt 2006), could also have been responsible. The timing of these forces' return to Canada in 1919 (Isitt 2006) lines up conspicuously well if Hearle's 1919 record of *Ae. dorsalis* from coastal rock pools in Caulfield Cove, West Vancouver (Hearle 1926), was mistaken from *Ae. togoi* (Trimble and Wellington 1979).

Ultimately, our modeling is based on climate data alone and is subject to the realities of limited sampling and, thus, cannot definitely prove or disprove either an anthropogenic introduction or Beringian dispersal for the presence of *Ae. togoi* in North America. Different modelling approaches (e.g. random forest, Bioclim, boosted regression tree) can also produce slightly different projections, indicating the inherent uncertainty present in any individual approach (Shabani et al. 2016). In future studies it may be enlightening to compare results between different modelling approaches or create an ensemble combining several modelling approaches (Araújo and New 2007, Shabani et al. 2016). However, even single modelling approaches such as that used in this study (Maxent) can be used to inform survey efforts aimed at detection of additional populations of species of interest (Law et al. 2017, Rhoden et al. 2017). We propose that future survey efforts for *Ae. togoi* should be directed at predicted areas of suitable habitat in Kamchatka, the Aleutian Islands (particularly Umnak and Unalaska), Cook Inlet, the Alexander Archipelago, and the North Coast of British Columbia. We also note a need for *Ae. togoi* surveillance across its novel predicted range in more heavily-surveyed areas, including the Hawaiian Islands and the Mariana Archipelago. To further investigate the origins of *Ae. togoi* in North America, we propose a combination of survey efforts and population genetic analyses based on mitochondrial and nuclear genome sequencing (Lee et al. 2019).

Acknowledgements

We thank Karen Needham of the UBC Beaty Biodiversity Museum and Claudia Copley of the Royal BC Museum for access to specimens. We additionally thank Alistair Blachford of the Zoology Computing Unit, UBC for technical assistance and L. Melissa Guzman and Katie Marshall for helpful discussions. We thank an anonymous reviewer for their thoughtful comments that improved the manuscript.

References

Alto, B. W., and S. A. Juliano. **2001**. Precipitation and temperature effects on populations of *Aedes albopictus* (Diptera: Culicidae): Implications for range expansion. J Med Entomol. 38: 646–656.

Araújo, M. B., and M. New. **2007**. Ensemble forecasting of species distributions. Trends in Ecology & Evolution. 22: 42–47.

Ashraf, U., A. T. Peterson, M. N. Chaudhry, I. Ashraf, Z. Saqib, S. R. Ahmad, and H. Ali. **2017**. Ecological niche model comparison under different climate scenarios: a case study of *Olea* spp. in Asia. Ecosphere. 8: e01825.

Becker, N., D. Petric, M. Zgomba, C. Boase, M. Minoo, C. Dahl, and A. Kaiser. **2010**. Mosquitoes and Their Control Second Edition. Springer, Heidelberg.

Belton, P. 1980. First record of *Aedes togoi* (Theo.) in the United States - aboriginal or ferry passenger? Mosquito News. 40: 624–626.

Belton, P. 1983. The Mosquitoes of British Columbia, Natural History. British Columbia Provincial Museum, Victoria, Canada.

Belton, P., and O. Belton. **1989**. *Aedes togoi* comes aboard. Journal of the American Mosquito Control Association. 6: 328–329.

Bohart, R. M. 1956. Insects of Micronesia. Volume 12, Diptera: Culicidae. Bernice P. Bishop Museum.

Cheun, H. I., S. H. Cho, H. I. Lee, E. H. Shin, J. S. Lee, T. S. Kim, and W. J. Lee. **2011**. Seasonal prevalence of mosquitoes, including vectors of Brugian filariasis, in Southern Islands of the Republic of Korea. Korean Journal of Parasitology. 49: 59–64.

Cunze, S., L. K. Koch, J. Kochmann, and S. Klimpel. **2016**. *Aedes albopictus* and *Aedes japonicus* - two invasive mosquito species with different temperature niches in Europe. Parasites and Vectors. 9: 1–12.

Cunze, S., J. Kochmann, L. K. Koch, and S. Klimpel. **2018**. Niche conservatism of *Aedes albopictus* and *Aedes aegypti* - two mosquito species with different invasion histories. Sci Rep. 8: 7733.

Darsie, R. F. J., and R. A. Ward. **2005**. Identification and Geographical Distribution of the Mosquitoes of North America, North of Mexico. University Press of Florida.

Ehlers, J., P. L. Gibbard, and P. D. Hughes. **2011**. Quaternary Glaciations - Extent and Chronology, Volume 15: A Closer Look, 1st Edition. ed. Elsevier.

Elith, C. H. Graham, R. P. Anderson, M. Dudik, S. Ferrier, A. Guisan, R. J. Hijmans, F. Huettmann, J. R. Leathwick, A. Lehmann, J. Li, L. G. Lohmann, B. A. Loiselle, G. Manion, C. Moritz, M. Nakamura, Y. Nakazawa, J. M. Overton, A. T. Peterson, S. J. Phillips, K. Richardson, R. Scachetti-Pereira, R. E. Schapire, J. Soberon, S. Williams, M. S. Wisz, and N. E. Zimmermann. **2006**. Novel methods improve prediction of species' distributions from occurrence data. Ecography. 29: 129–151.

Gent, P. R., G. Danabasoglu, L. J. Donner, M. M. Holland, E. C. Hunke, S. R. Jayne, D. M. Lawrence, R. B. Neale, P. J. Rasch, M. Vertenstein, P. H. Worlez, Z. Yang, and M. Zhang. **2011**. The community climate system model version 4. Journal of Climate. 24: 4973–4991.

Gibson, J. R. 1980. Russian expansion in Siberia and America. Geographical Review. 70: 127.

Hanson, S. M., and G. B. Craig. **1995**. *Aedes albopictus* (Diptera: Culicidae) eggs: Field survivorship during Northern Indiana winters. Journal of Medical Entomology. 32: 599–604.

Hawley, W., P. Reiter, R. Copeland, C. Pumpuni, and G. Craig. **1987**. *Aedes albopictus* in North America: probable introduction in used tires from northern Asia. Science. 236: 1114–1116.

Hayashi, S. 2011. Brugian Filariasis in Japan. Tropical Medicine and Health. 39: 25–28.

Hearle, E. 1926. The mosquitoes of the lower Fraser Valley, British Columbia, and their control. Natural Research Council of Canada, Report 17. 94.

Herrando-Moraira, S., N. Nualart, A. Herrando-Moraira, M. Y. Chung, M. G. Chung, and J. López-Pujol. **2019**. Climatic niche characteristics of native and invasive *Lilium lancifolium*. Sci Rep. 9: 14334.

Hijmans, R. J., S. E. Cameron, J. L. Parra, P. G. Jones, and A. Jarvis. **2005**. Very high resolution interpolated climate surfaces for global land areas. International Journal of Climatology. 25: 1965–1978.

Hsiao, T.-Y., and R. M. Bohart. **1946**. The mosquitoes of Japan and their medical importance, Navmed. United States Department of the Navy.

Isitt, B. 2006. Mutiny from Victoria to Vladivostok, December 1918. The Canadian Historial Review. 87: 1–45.

Kaufman, M. G., and D. M. Fonseca. **2014**. Invasion biology of *Aedes japonicus japonicus* (Diptera: Culicidae). Annual Review of Entomology. 59: 31–49.

Kavanaugh, D. H. 1988. The insect fauna of the Pacific Northwest coast of North America: present patterns and affinities and their origins. Memoirs of the Entomological Society of Canada. 125–149.

Kim, H., and B.-S. Seo. **1968**. Studies on filariasis in Korea - On the morphology and development of larvae of Brugia malayi in *Aedes togoi*. Korean Journal of Parasitology. 6: 1–10.

Law, B., G. Caccamo, P. Roe, A. Truskinger, T. Brassil, L. Gonsalves, A. McConville, and M. Stanton. **2017**. Development and field validation of a regional, management-scale habitat model: A koala Phascolarctos cinereus case study. Ecology and Evolution. 7: 7475–7489.

Lee, J. S., and H. K. Hong. **1995**. Seasonal prevalence and behaviour of *Aedes togoi*. The Korean journal of parasitology. 33: 19–26.

Lee, Y., H. Schmidt, T. C. Collier, W. R. Conner, M. J. Hanemaaijer, M. Slatkin, J. M. Marshall, J. C. Chiu, C. T. Smartt, G. C. Lanzaro, F. S. Mulligan, and A. J. Cornel. **2019**. Genome-wide divergence among invasive populations of *Aedes aegypti* in California. BMC Genomics. 20: 204.

Lien, J. C. 1962. Non-Anopheline mosquitoes of Taiwan: Annotated catalog and bibliography. Pacific Insects. 4: 615–649.

Lightfoot, K. G. 2003. Russian colonization: The implications of mercantile colonial practices in the North Pacific. Historical Archaeology. 37: 14–28.

Liu, C., P. M. Berry, T. P. Dawson, and R. G. Pearson. **2005**. Selecting thresholds of occurrence in the prediction of species distributions. Ecography. 28: 385–393.

Lounibos, L. P. 2002. Invasions by insect vectors of human disease. Annual Review of Entomology. 47: 233–266.

Lubinda, J., J. A. Treviño C., M. R. Walsh, A. J. Moore, A. A. Hanafi-Bojd, S. Akgun, B. Zhao, A. S. Barro, M. M. Begum, H. Jamal, A. Angulo-Molina, and U. Haque. **2019**. Environmental suitability for *Aedes aegypti* and *Aedes albopictus* and the spatial distribution of major arboviral infections in Mexico. Parasite Epidemiology and Control. 6: e00116.

Lyons, C. L., M. Coetzee, and S. L. Chown. **2013**. Stable and fluctuating temperature effects on the development rate and survival of two malaria vectors, *Anopheles arabiensis* and *Anopheles funestus*. Parasites Vectors. 6: 104.

Melaun, C., A. Werblow, S. Cunze, S. Zotzmann, L. K. Koch, H. Mehlhorn, D. D. Dörge, K. Huber, O. Tackenberg, and S. Klimpel. **2015**. Modeling of the putative distribution of the arbovirus vector *Ochlerotatus japonicus japonicus* (Diptera: Culicidae) in Germany. Parasitology Research. 114: 1051–1061.

Merow, C., M. J. Smith, and J. A. Silander. **2013**. A practical guide to MaxEnt for modeling species' distributions: what it does, and why inputs and settings matter. Ecography. 36: 1058–1069.

Omori, N. 1962. A review of the role of mosquitos in the transmission of malayan and bancroftian filariasis in Japan*. Bulletin of the World Health Organization. 27: 585–594.

Otto-Bliesner, B. L., S. J. Marshall, J. T. Overpeck, G. H. Miller, and A. Hu. **2009**. Simulating Arctic climate warmth and icefield retreat in the last interglaciation. Science. 311: 1751–1755.

Padalia, H., V. Srivastava, and S. P. S. Kushwaha. **2014**. Modeling potential invasion range of alien invasive species, *Hyptis suaveolens* (L.) Poit. in India: Comparison of MaxEnt and GARP. Ecological Informatics. 22: 36–43.

Peach, D. A. H. 2018. An updated list of the mosquitoes of British Columbia with distribution notes. Journal of the Entomological Society of British Columbia. 115: 126–129.

Peach, D. A. H., M. Almond, and J. Pol. **2019**. Modeled distributions of *Aedes japonicus* and *Aedes togoi* (Diptera: Culicidae) in the United States, Canada, and northern Latin America. Journal of Vector Ecology. 44: 119–129.

Petersen, W. H., M. Lyons, M. Westlind, A. Duffy, D. Kangiser, and E. A. Dykstra. **2017**. New distribution records of mosquitoes in Washington State. The American Mosquito Control Association. 33: 60–63.

Petrishcheva, P. 1948. On the transition of *Aedes togoi* Theob. and *Aedes japonicus* Theob. (Diptera, Culicidae) to a synanthropic form of life. Entomologicheskoye Obozreniye. 30: 103–108.

Phillips, S. J., R. P. Anderson, M. Dudík, R. E. Schapire, and M. E. Blair. **2017**. Opening the black box: an open-source release of Maxent. Ecography. 40: 887–893.

Phillips, S. J., R. P. Anderson, and R. E. Schapire. **2006**. Maximum entropy modeling of species geographic distributions. Ecological Modeling. 190: 231–259.

Phillips, S. J., M. Dudík, and R. E. Schapire. **2018**. Maxent 3.4.1 software for modeling species niches and distributions.

Porretta, D., V. Mastrantonio, R. Bellini, P. Somboon, and S. Urbanelli. **2012**. Glacial history of a modern invader: Phylogeography and species distribution modelling of the Asian Tiger mosquito *Aedes albopictus*. PLoS ONE. 7: e44515.

QGIS Development Team. **2018**. QGIS Geographic Information System.

Ramalingam, S. 1969. New record of *Aedes* (Finlaya) *togoi* (Theobold) in West Malaysia. Med J Malaya. 23: 288–292.

Reinhold, J., C. Lazzari, and C. Lahondère. **2018**. Effects of the environmental Temperature on *Aedes aegypti* and *Aedes albopictus* mosquitoes: A review. Insects. 9: 158.

Rhoden, C. M., W. E. Peterman, and C. A. Taylor. **2017**. Maxent-directed field surveys identify new populations of narrowly endemic habitat specialists. PeerJ. 5: e3632.

Rochlin, I. 2019. Modeling the Asian longhorned tick (Acari: Ixodidae) suitable habitat in North America. Journal of Medical Entomology. 56: 384–391.

Rochlin, I., D. V. Ninivaggi, M. L. Hutchinson, and A. Farajollahi. **2013**. Climate change and range expansion of the Asian tiger mosquito (*Aedes albopictus*) in Northeastern USA: Implications for public health practitioners. PLoS ONE. 8: 1–9.

Rosen, L. 1986. The natural history of Japanese encephalitis virus. Annual Reviews in Microbiology. 40: 395–414.

Rosen, L., R. Tesh, J. Lien, and J. Cross. **1978**. Transovarial transmission of Japanese encephalitis virus by mosquitoes. Science. 199: 909–911.

Sames, J., I. Wlliam, A. Florin', E. Francis, and A. Maloneyi. **2004**. Distribution of *Ochlerotatus togoi* along the Pacific Coast of Washington. Journal of the American Mosquito Control Association. 20: 105–109.

Sazonova, O. N., and V. A. Smirnova. **1986**. Distribution area of the bloodsucking mosquito *Aedes togoi*. Parazitologiya. 20: 261–264.

Shabani, F., L. Kumar, and M. Ahmadi. **2016**. A comparison of absolute performance of different correlative and mechanistic species distribution models in an independent area. Ecology and Evolution. 6: 5973–5986.

Shafer, A. B. A., C. I. Cullingham, S. D. Côté, and D. W. Coltman. **2010**. Of glaciers and refugia: a decade of study sheds new light on the phylogeography of northwestern North America. Molecular Ecology. 19: 4589–4621.

Shestakov, V. 1961. On the biology of *Aedes togoi* Theob. Zoologichnyy Zhurnal. 40: 284–285.

Sollers-Riedel, H. 1971. World studies on mosquitoes and diseases carried by them, pp. 1–52. *In* Proceedings of the 58th Annual Meeting of the New Jersey Mosquito Extermination Association. Supplement.

Sota, T. 1994. Larval diapause, size, and autogeny in the mosquito *Aedes togoi* (Diptera, Culicidae) from tropical to subarctic zones. Canadian Journal of Zoology. 72: 1462–1468.

Sota, T., P. Belton, M. Tseng, H. S. Yong, and M. Mogi. **2015**. Phylogeography of the coastal mosquito *Aedes togoi* across climatic zones: Testing an anthropogenic dispersal hypothesis. PLoS ONE. 10: e0131230.

Stephen, C., N. Plamondon, and P. Belton. **2006**. Notes on the distribution of mosquito species that could potentially transmit West Nile virus on Vancouver Island, British Columbia. Journal of the American Mosquito Control Association. 22: 553–556.

Stockwell, D. R. B., and A. T. Peterson. **2002**. Effects of sample size on accuracy of species distribution models. Ecological Modelling. 148: 1–13.

Swets, J. 1988. Measuring the accuracy of diagnostic systems. Science. 240: 1285–1293.

Tanaka, K., K. Mizusawa, and E. S. Saugstad. **1979**. A revision of the adult and larval mosquitoes of Japan (Including the Ryukyu Archipelago and the Ogasawara Islands) and Korea (Diptera: Culicidae). Contributions of the American Entomological Institute. 16: 989.

Tanaka, K., E. S. Saugstad, and K. Mizusawa. **1975**. Mosquitoes of the Ryukyu Archipelago (Diptera: Culicidae). Mosquito Systematics. 7: 207–233.

Theobald, F. V. 1907. A monograph of the Culicidae of the world Vol IV. British Museum (Natural History), London.

Trimble, R. M. 1984. *Aedes togoi* (Diptera: Culicidae) dispersal: Assessment using artificial container habitats and miniature light traps. Journal of Medical Entomology. 21: 120–121.

Trimble, R., and W. Wellington. **1979**. Colonization of North American *Aedes togoi*. Mosquito News. 39: 18–20.

Wada, Y. 2011. Vector mosquitoes of filariasis in Japan. Tropical Medicine and Health. 39: 39–42.

Wada, Y., S. Ito, and T. Oda. **1993**. Seasonal abundance of immature stages of *Aedes togoi* at Fukue Island, Nagasaki (Diptera: Culicidae). Tropical Medicine. 35: 1–10.

Wang, C., D. Hawthorne, Y. Qin, X. Pan, Z. Li, and S. Zhu. **2017**. Impact of climate and host availability on future distribution of Colorado potato beetle. Sci Rep. 7: 4489.

Warren, D. L., R. E. Glor, and M. Turelli. **2010**. ENMTools: A toolbox for comparative studies of environmental niche models. Ecography. 33: 607–611.

Warren, D. L., and S. N. Seifert. **2011**. Ecological niche modeling in Maxent: the importance of model complexity and the performance of model selection criteria. Ecological Applications. 21: 335–342.

West, A. M., S. Kumar, C. S. Brown, T. J. Stohlgren, and J. Bromberg. **2016**. Field validation of an invasive species Maxent model. Ecological Informatics. 36: 126–134.

Wiens, J. A., D. Stralberg, D. Jongsomjit, C. A. Howell, and M. A. Snyder. **2009**. Niches, models, and climate change: Assessing the assumptions and uncertainties. Proceedings of the National Academy of Sciences. 106: 19729–19736.

Wiens, J. J., and C. H. Graham. **2005**. Niche conservatism: Integrating evolution, ecology, and conservation biology. Annu. Rev. Ecol. Evol. Syst. 36: 519–539.

Wood, D. M., P. T. Dang, and R. A. Ellis. **1979**. The Insects and Arachnids of Canada Part 6: The Mosquitoes of Canada - Diptera: Culicidae, The Insects and Arachnids of Canada. Research Branch, Agriculture Canada, Ottawa, Canada.

Yackulic, C. B., R. Chandler, E. F. Zipkin, J. A. Royle, J. D. Nichols, E. H. Campbell Grant, and S. Veran. **2013**. Presence-only modelling using MAXENT: When can we trust the inferences? Methods in Ecology and Evolution. 4: 236–243.

Ye, Z., G. Zhu, P. Chen, D. Zhang, and W. Bu. **2014**. Molecular data and ecological niche modelling reveal the Pleistocene history of a semi-aquatic bug (*Microvelia douglasi douglasi*) in East Asia. Mol Ecol. 23: 3080–3096.

Tables

Table 1. The Worldclim bioclimatic variables.

Code	Variable
BIO 01	Annual Mean Temperature
BIO 02	Mean Dirunal Range
BIO 03	Isothermality
BIO 04	Temperature Seasonality
BIO 05	Maximum Temperature of the Warmest Month
BIO 06	Minimum Temperature of the Coldest Month
BIO 07	Temperature Annual Range
BIO 08	Mean Temperature of the Wettest Quarter
BIO 09	Mean Temperature of the Driest Quarter
BIO 10	Mean Temperature of the Warmest Quarter
BIO 11	Mean Temperature of the Coldest Quarter
BIO 12	Annual Precipitation
BIO 13	Precipitation of the Wettest Month
BIO 14	Precipitation of the Driest Month
BIO 15	Precipitation Seasonality
BIO 16	Precipitation of the Wettest Quarter
BIO 17	Precipitation of the Driest Quarter
BIO 18	Precipitation of the Warmest Quarter
BIO 19	Precipitation of the Coldest Quarter

Table 2. The top candidate models based on AICc and AUC, with the final model constructed from Asian *Ae. togoi* records (model 1) bolded.

Occurrence Records	Variables	Parameters	Regularization	Features	AICc	AUC	SD
**Asia**	**Bio 02, 06, 10, 13, 18, 19**	**33**	**1**	**Without Linear**	**2600.3**	**0.912**	**0.031**
Asia	Bio 02, 06, 10, 13, 18, 19	25	2	Without Linear	2600.8	0.898	0.035
Asia	Bio 02, 05, 06, 13, 18, 19	34	1	Default	2607.2	0.915	0.031

Table 3. The top candidate models based on AICc and AUC, with the final model constructed from all known *Ae. togoi* records (model 2) bolded.

Occurrence Records	Variables	Parameters	Regularization	Features	AICc	AUC	SD
**All**	**Bio 02, 06, 10, 14, 15, 16**	**37**	**1**	**Without Linear**	**4041.1**	**0.972**	**0.009**
All	Bio 02, 06, 10, 15, 16, 17	25	2	Without Linear	4042.2	0.971	0.01
All	Bio 02, 06, 10, 15, 16, 17	41	1	Without Quadratic	4054.9	0.973	0.01

Table 4. The relative permutation importance of the variables used in the *Ae. togoi* habitat models.

Relative importance (%) to the Maxent models		
Variable	Model 1	Model 2
Mean diurnal range (BIO 02)	47	32.1
Minimum temperature of the coldest month (BIO 06)	17.7	26.1
Mean temperature of the warmest quarter (BIO 10)	6.4	27.6
Precipitation of the wettest month (BIO 13)	5	-
Precipitation of the driest month (BIO 14)	-	6.9
Precipitation seasonality (BIO 15)	-	4.3
Precipitation of the wettest quarter (BIO 16)	-	3.0
Precipitation of the warmest quarter (BIO 18)	6.6	-
Precipitation of the coldest quarter (BIO 19)	17.4	-

Figures

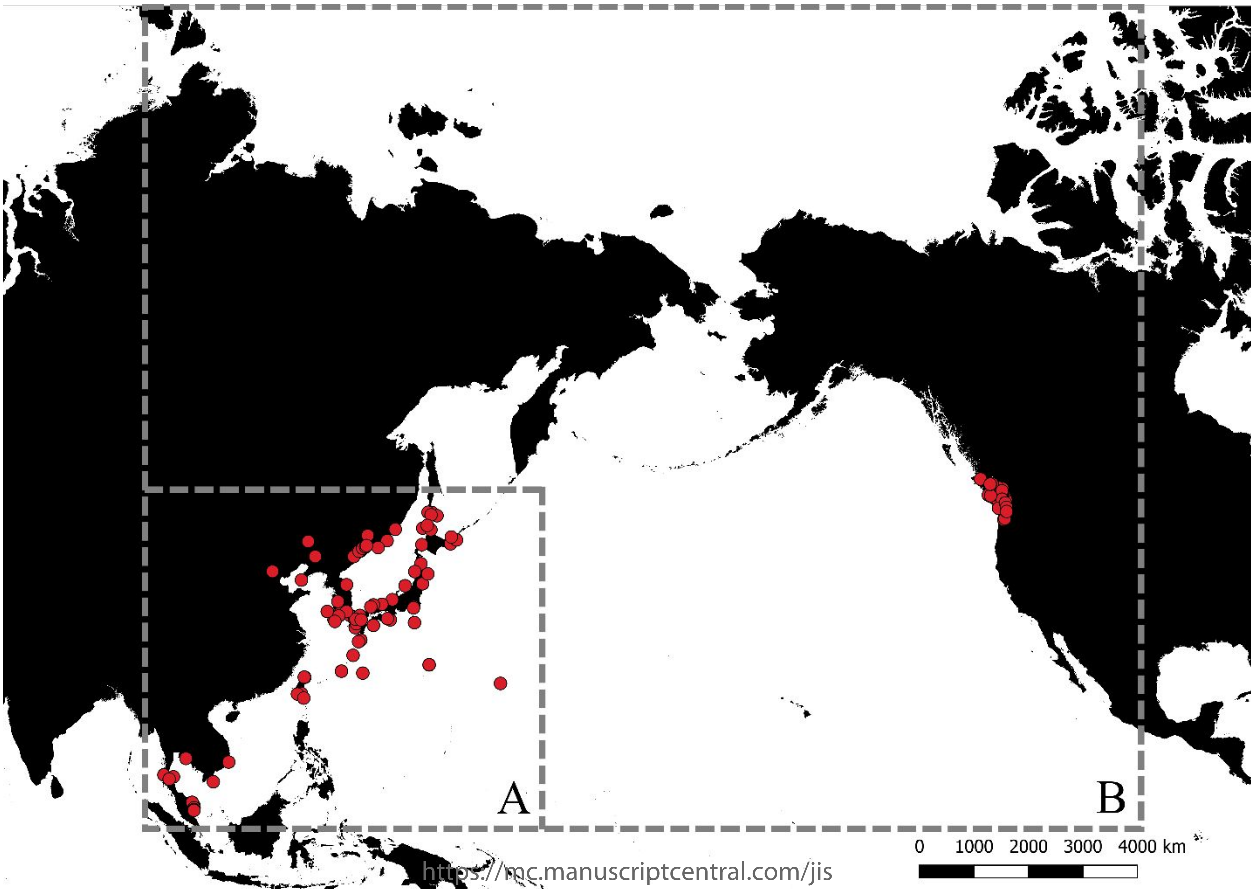

Figure 1. Worldwide distribution records of *Aedes togoi* used in this study (N = 133), with hash marks indicating (A) the Asian study area used to create model 1, and (B) the study area used to create model 2.

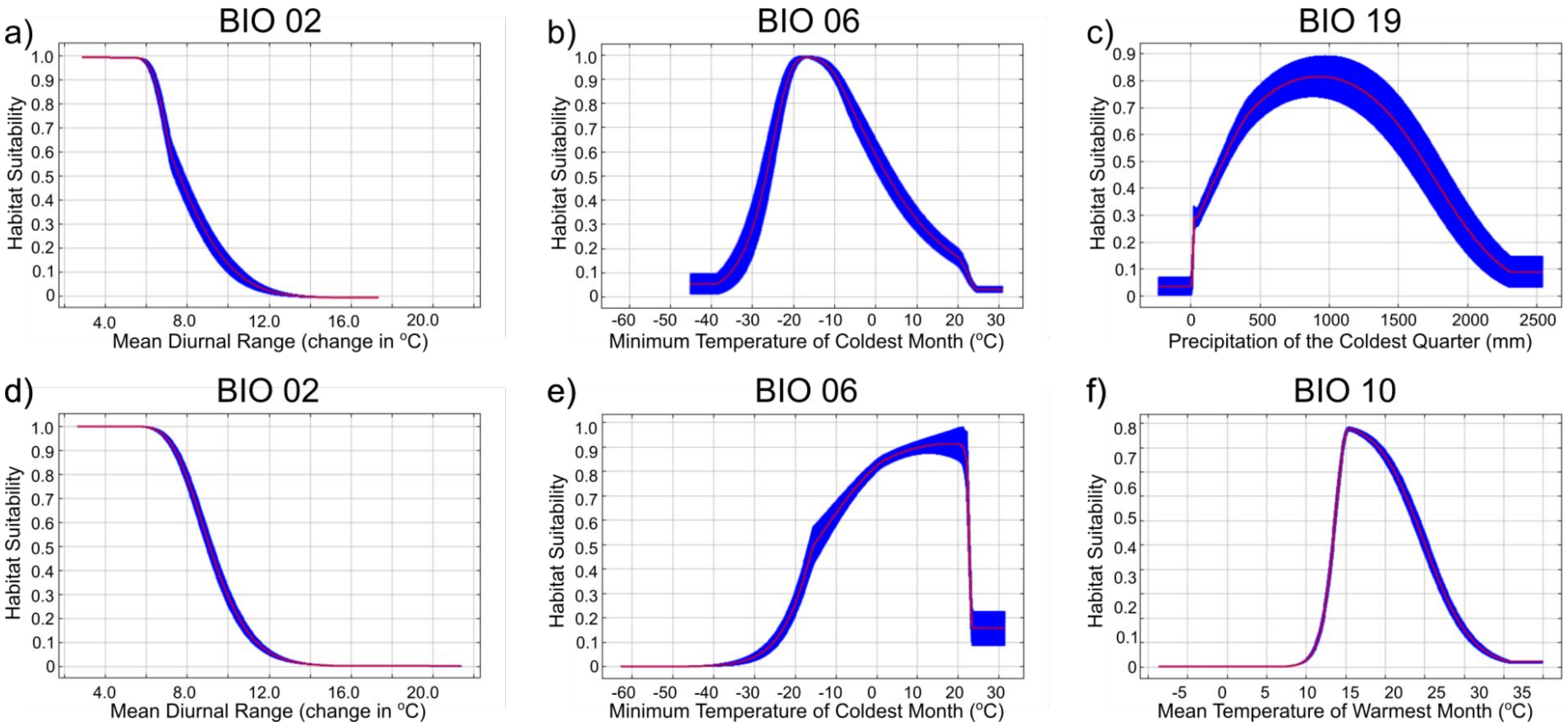

Figure 2. Marginal response curves (mean ±SD) of environmental variables and *Ae. togoi* habitat suitability for model 1 (a,b,c) and model 2 (d,e,f).

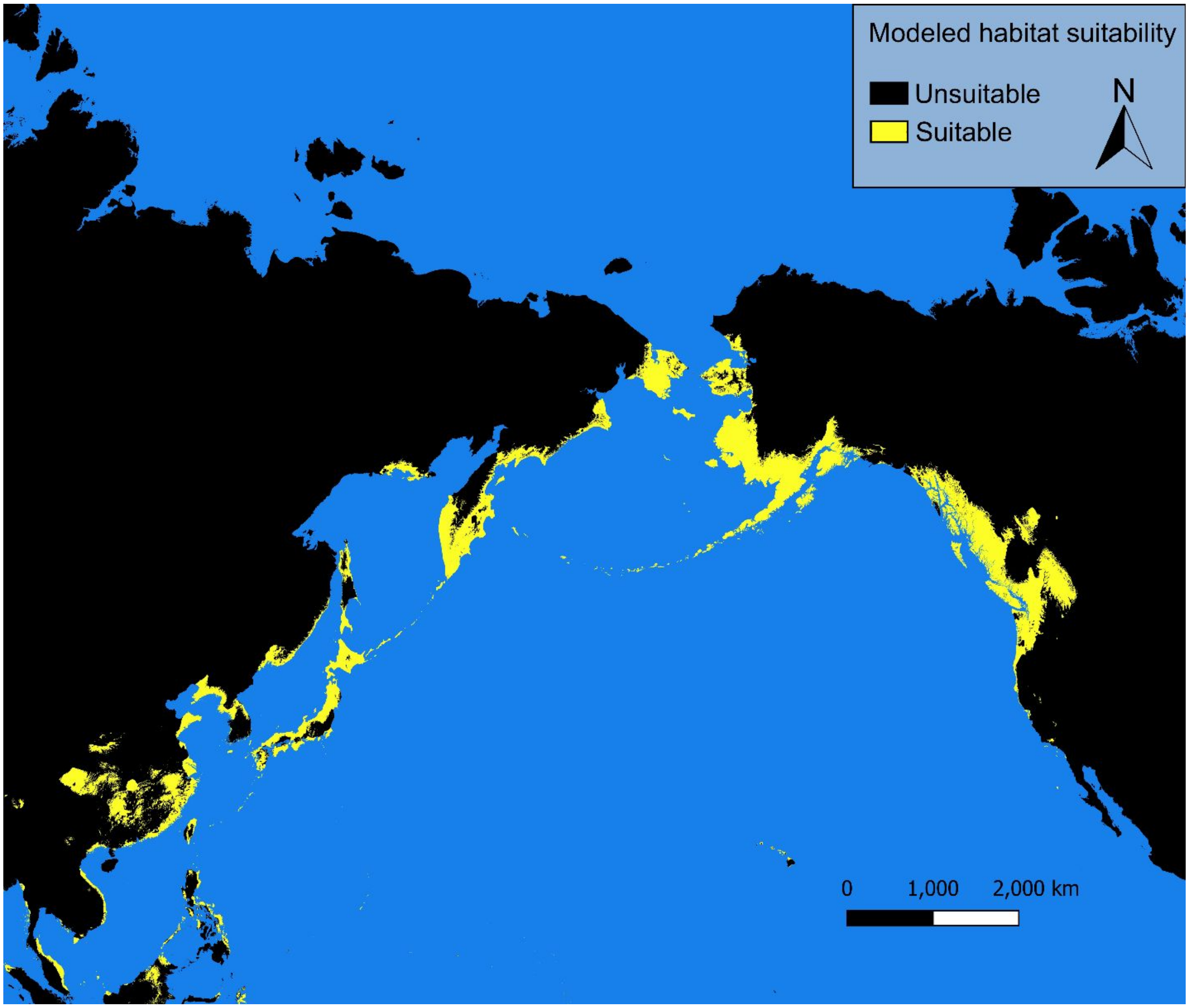

Figure 3. Current distribution of potential *Ae. togoi* predicted by model 1.

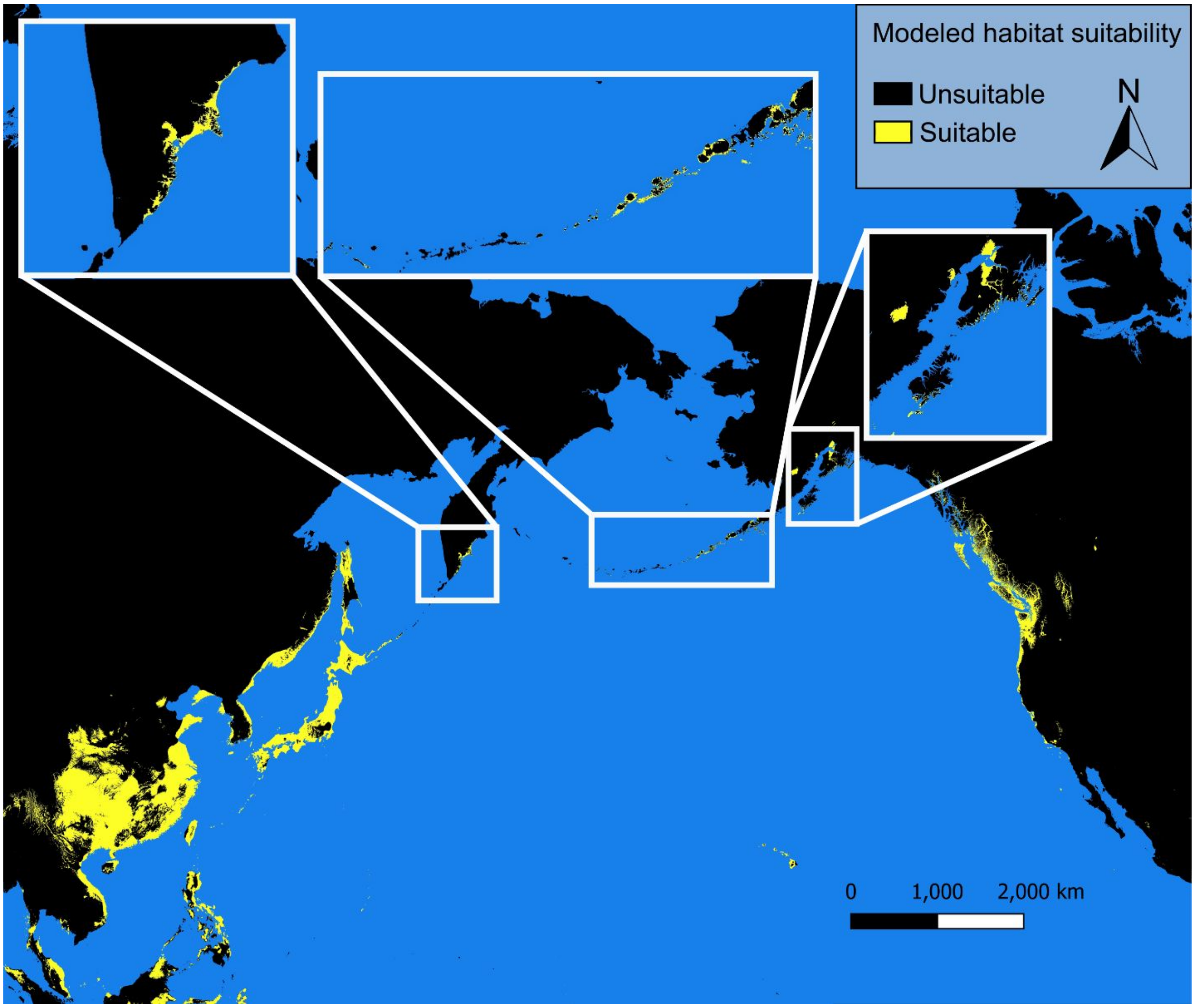

Figure 4. Current distribution of potential *Ae. togoi* predicted by model 2.

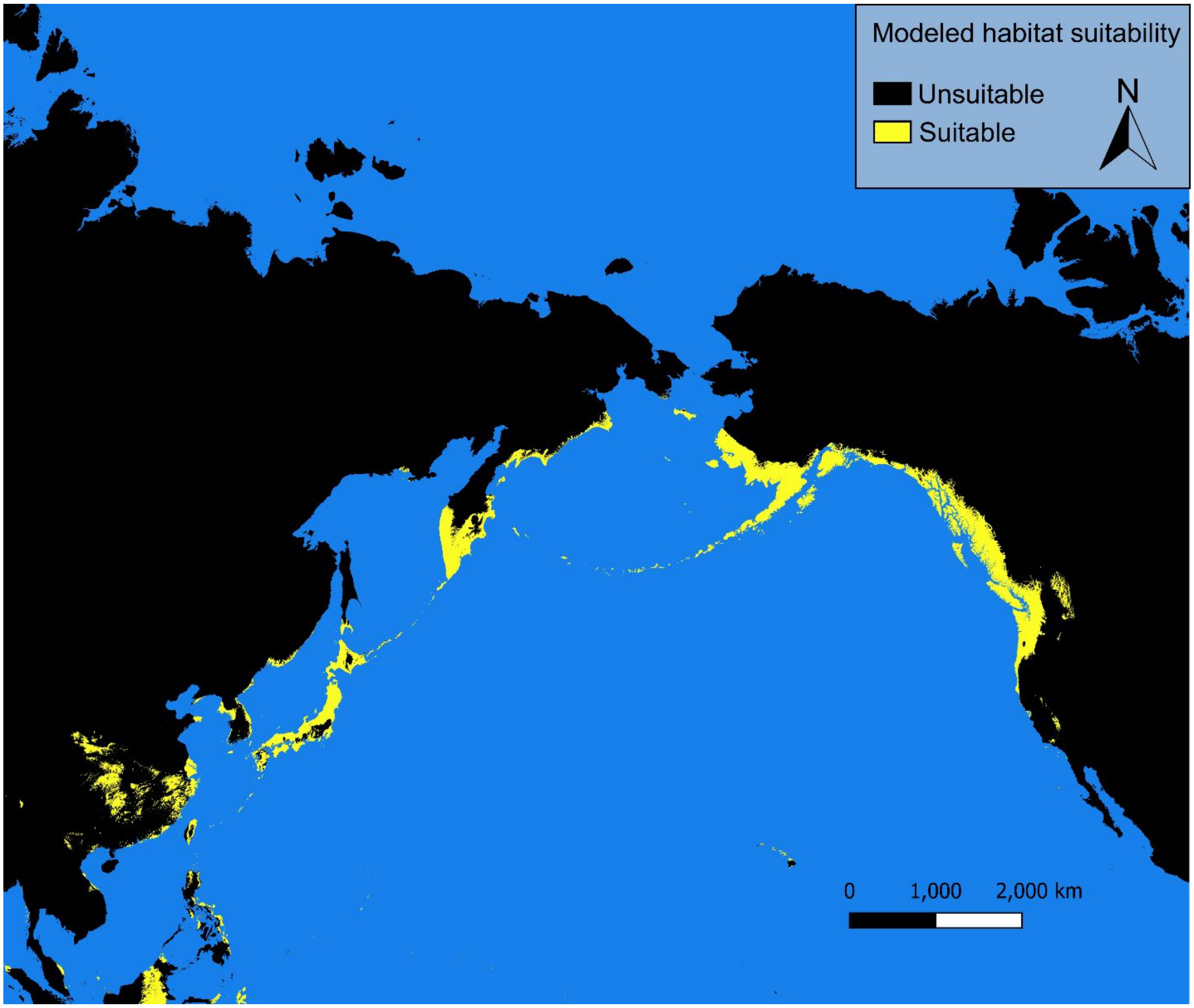

Figure 5. Paleodistribution of potential *Ae. togoi* habitat during the last interglacial period predicted by model 1.

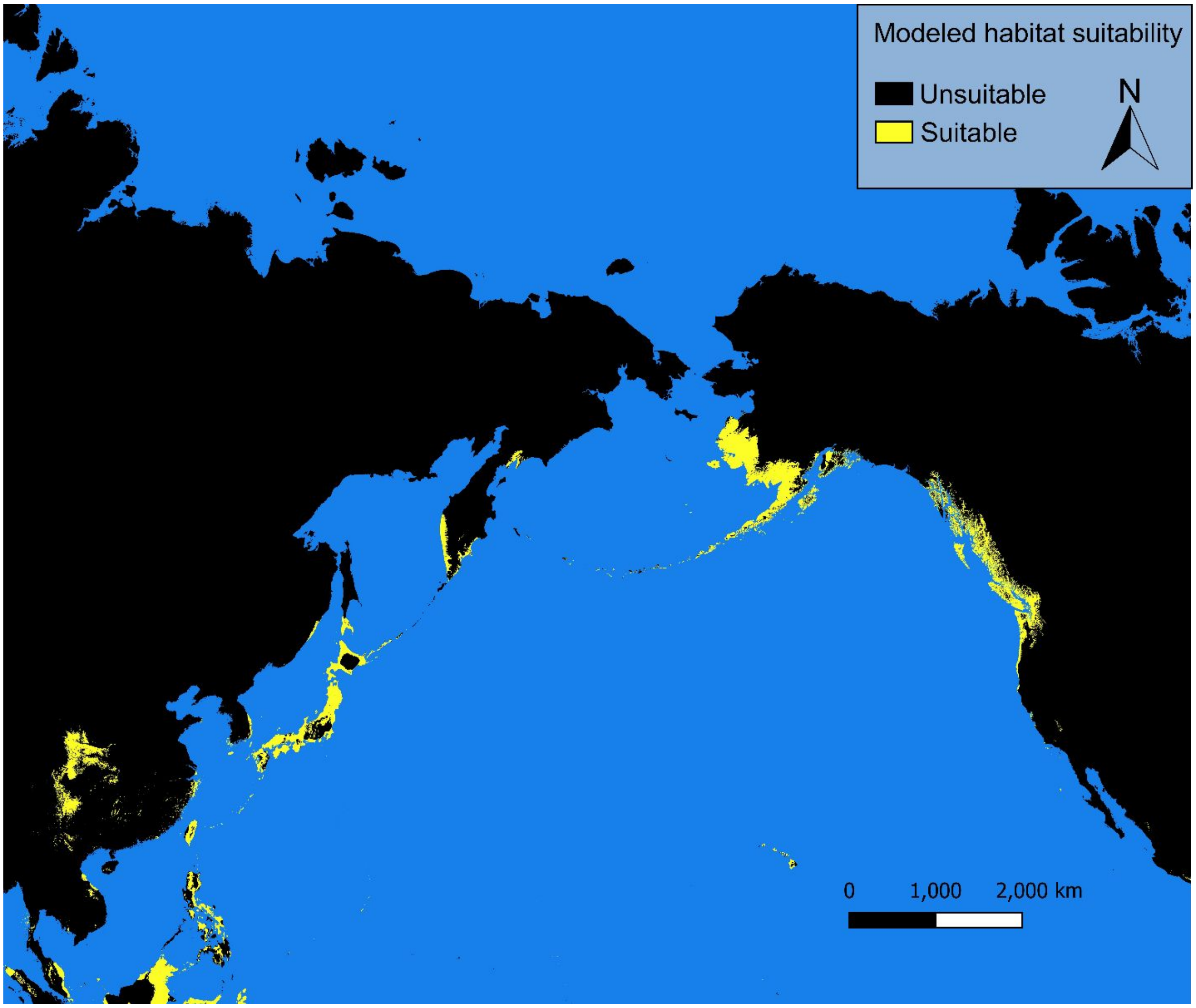

Figure 6. Paleodistribution of potential *Ae. togoi* habitat during the last interglacial period predicted by model 2.

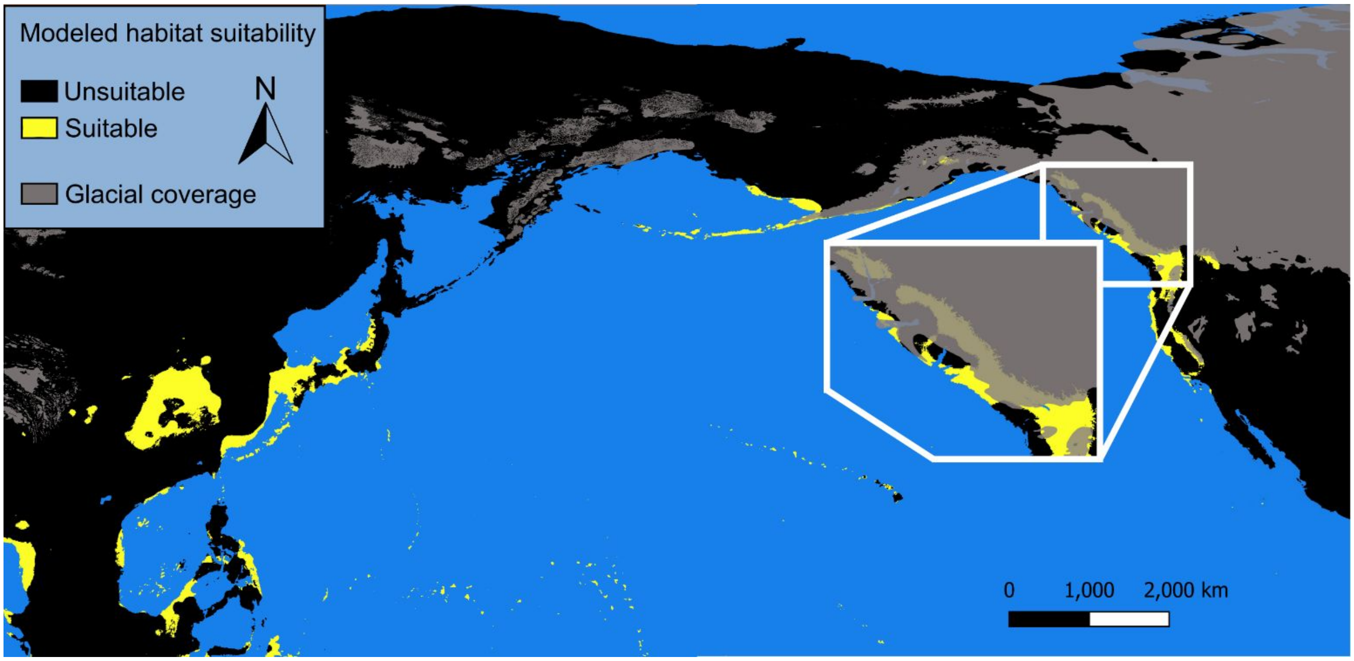

Figure 7. Paleodistribution of potential *Ae. togoi* habitat during the last glacial maximum predicted by model 1, with glacial coverage.

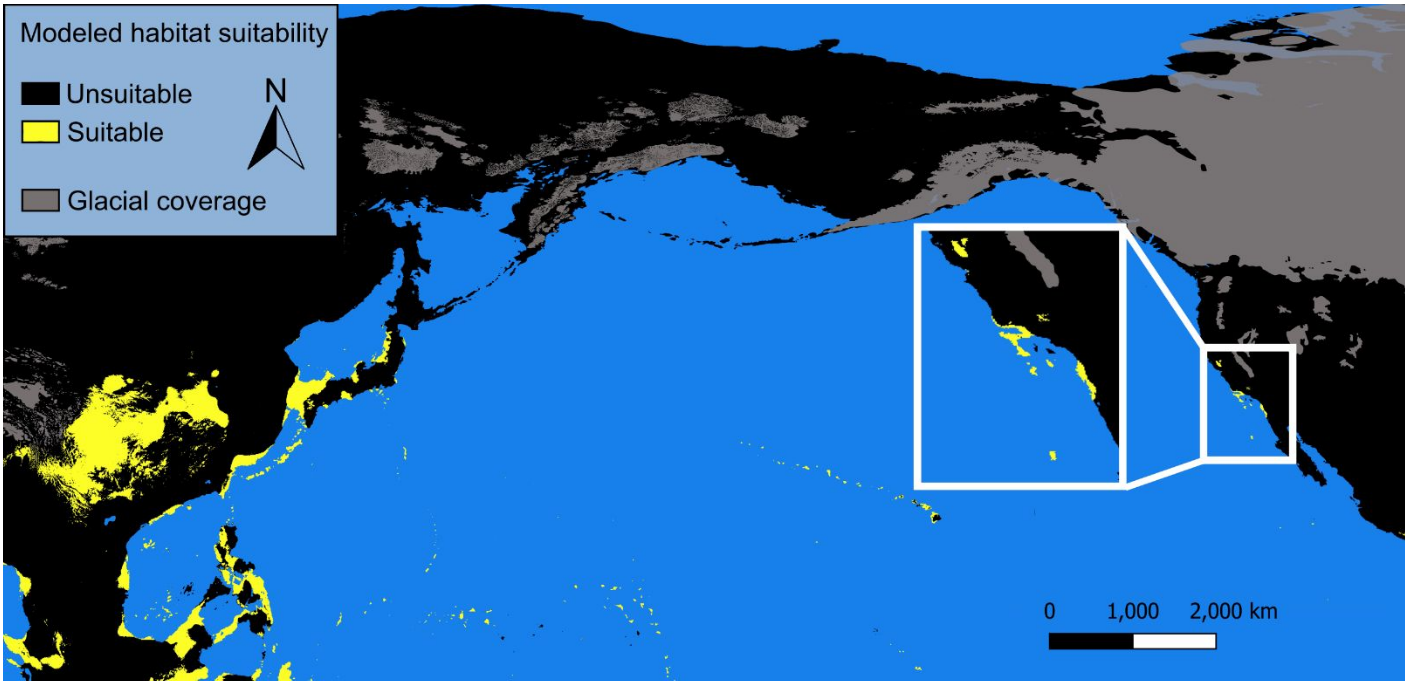

Figure 8. Paleodistribution of potential *Ae. togoi* habitat during the last glacial maximum predicted by model 2, with glacial coverage.

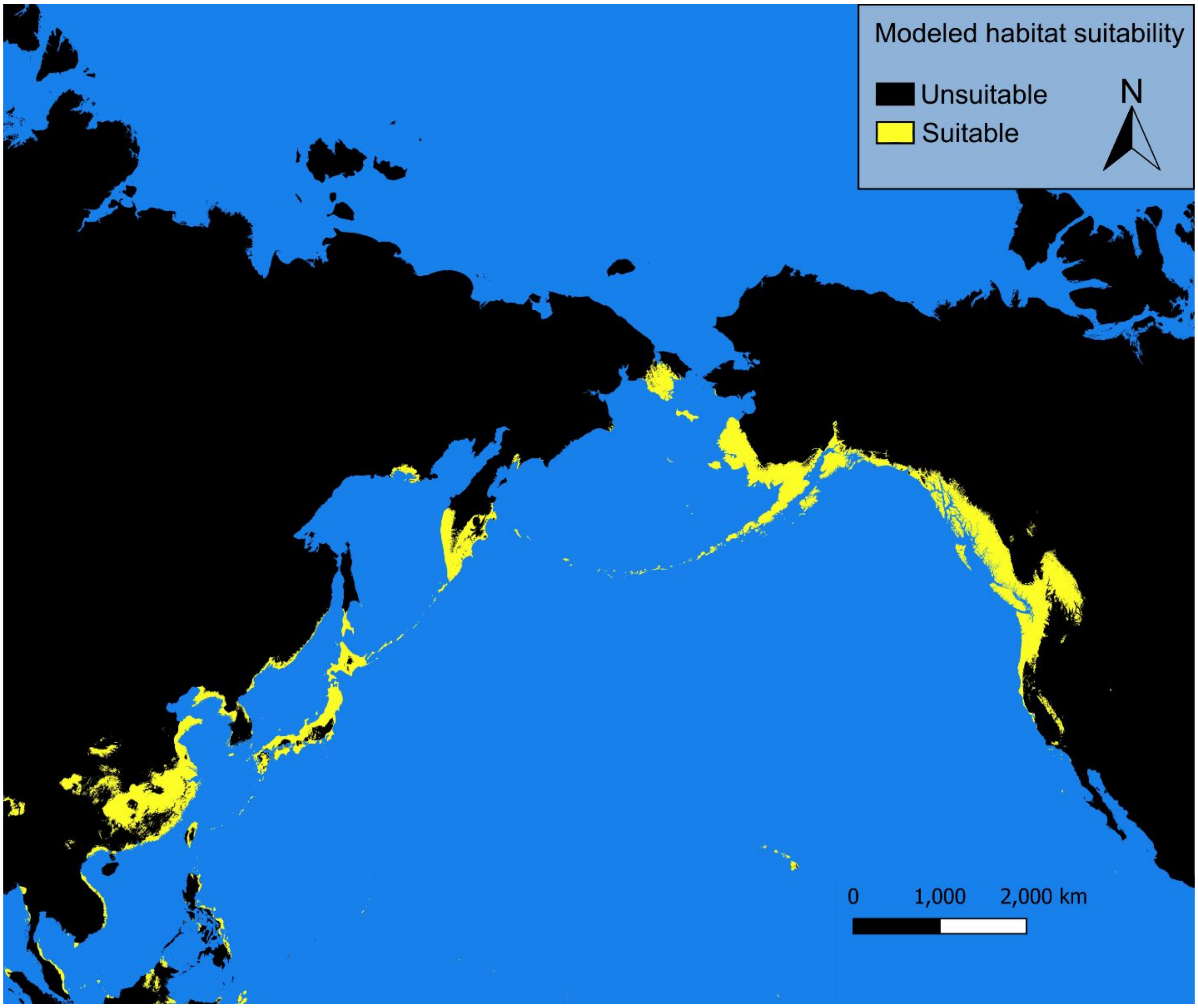

Figure 9. Paleodistribution of potential *Ae. togoi* habitat during the mid-Holocene period predicted by model 1.

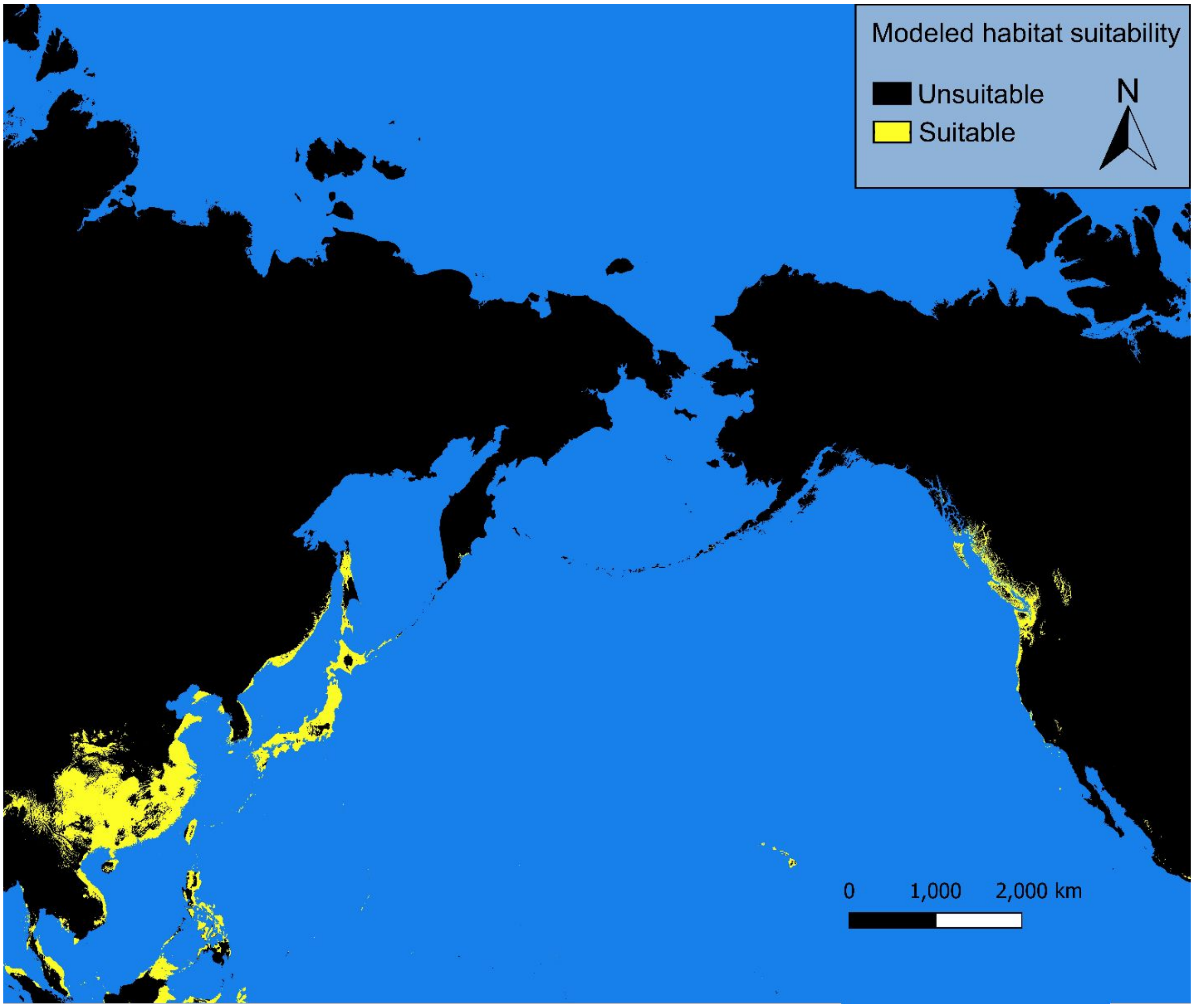

Figure 10. Paleodistribution of potential *Ae. togoi* habitat during the mid-Holocene period predicted by model 2.

Species	Lattitude	Longitude	Source
Aedes togo	49.334	-123.266	Dan Peach, Pers. Obs.
Aedes togo	49.37234	-123.29	Dan Peach, Pers. Obs.
Aedes togo	48.997	-123.583	UBC Spencer Entomology Collection
Aedes togo	49.514	-123.906	UBC Spencer Entomology Collection
Aedes togo	48.525	-123.366	UBC Spencer Entomology Collection
Aedes togo	48.52939	-124.45	Dan Peach Pers. Obs.
Aedes togo	48.67545	-123.397	Stephen et al., 2006
Aedes togo	48.8517	-123.494	Stephen et al., 2006
Aedes togo	50.136	-125.362	Stephen et al., 2006
Aedes togo	48.77784	-125.151	Stephen et al., 2006
Aedes togo	48.926	-125.541	Dan Peach Pers. Obs.
Aedes togo	48.426	-124.05	Royal BC Museum
Aedes togo	48.49782	-122.698	Sames et al., 2004
Aedes togo	48.41673	-122.665	Sames et al., 2004
Aedes togo	48.5395	-122.862	Sames et al., 2004
Aedes togo	48.61745	-123.048	Sames et al., 2004
Aedes togo	48.718	-122.7	Sames et al., 2004
Aedes togo	48.744	-122.835	Sames et al., 2004
Aedes togo	48.59264	-122.924	Sames et al., 2004
Aedes togo	48.553	-123.011	Sames et al., 2004
Aedes togo	48.56	-122.978	Sames et al., 2004
Aedes togo	48.40395	-122.648	Sames et al., 2004
Aedes togo	48.52775	-123.365	Belton and Belton, 1990
Aedes togo	48.567	-123.278	Belton and Belton, 1990
Aedes togo	48.6073	-123.159	Belton and Belton, 1990
Aedes togo	48.75965	-123.235	Belton and Belton, 1990
Aedes togo	48.76614	-123.288	Belton and Belton, 1990
Aedes togo	49.62396	-123.207	Belton and Belton, 1990
Aedes togo	49.42633	-123.658	Belton and Belton, 1990
Aedes togo	49.6343	-124.058	Belton and Belton, 1990
Aedes togo	49.75293	-124.008	Belton and Belton, 1990
Aedes togo	49.95122	-124.996	Belton and Belton, 1990
Aedes togo	50.11786	-124.912	Belton and Belton, 1990
Aedes togo	50.6254	-126.834	Dan Peach Pers. Obs.
Aedes togo	49.3701	-123.29	Sota et al., 2015
Aedes togo	48.4561	-123.269	Sota et al., 2015
Aedes togo	48.85005	-125.167	Sota et al., 2015
Aedes togo	46.14946	-122.975	Peterson et al., 2017
Aedes togo	46.29708	-122.817	Peterson et al., 2017
Aedes togo	47.46133	-123.861	Peterson et al., 2017
Aedes togo	48.05035	-122.729	Peterson et al., 2017
Aedes togo	47.56592	-122.551	Peterson et al., 2017
Aedes togo	47.09935	-122.528	Peterson et al., 2017
Aedes togo	50.08382	-125.222	Dan Peach Pers. Obs.
Aedes togo	50.07727	-125.216	Dan Peach Pers. Obs.
Aedes togo	50.06838	-125.227	Dan Peach Pers. Obs.
Aedes togo	50.08175	-125.234	Dan Peach Pers. Obs.
Aedes togo	50.08378	-125.228	Dan Peach Pers. Obs.
Aedes togo	50.08363	-125.241	Dan Peach Pers. Obs.
Aedes togo	45.19243	141.1551	Sota et al., 2015
Aedes togo	43.3382	145.7518	Sota et al., 2015

Aedes togo	43.231	141.0159	Sota et al., 2015
Aedes togo	40.883	140.8587	Sota et al., 2015
Aedes togo	39.8942	139.8575	Sota et al., 2015
Aedes togo	38.0754	138.2439	Sota et al., 2015
Aedes togo	36.2372	136.1345	Sota et al., 2015
Aedes togo	35.6214	134.426	Sota et al., 2015
Aedes togo	32.9583	130.2199	Sota et al., 2015
Aedes togo	35.5403	133.0241	Sota et al., 2015
Aedes togo	35.273	132.5862	Sota et al., 2015
Aedes togo	38.3331	141.1254	Sota et al., 2015
Aedes togo	35.29	139.6791	Sota et al., 2015
Aedes togo	35.137	139.6278	Sota et al., 2015
Aedes togo	33.4992	135.799	Sota et al., 2015
Aedes togo	33.6669	135.3336	Sota et al., 2015
Aedes togo	32.7241	133.0202	Sota et al., 2015
Aedes togo	34.1092	129.177	Sota et al., 2015
Aedes togo	34.1007	130.7881	Sota et al., 2015
Aedes togo	32.424	130.009	Sota et al., 2015
Aedes togo	33.539	129.9322	Sota et al., 2015
Aedes togo	32.9583	130.2199	Sota et al., 2015
Aedes togo	33.5829	130.0939	Sota et al., 2015
Aedes togo	30.666	130.9425	Sota et al., 2015
Aedes togo	30.4325	130.5662	Sota et al., 2015
Aedes togo	28.4608	129.6755	Sota et al., 2015
Aedes togo	26.1214	127.7555	Sota et al., 2015
Aedes togo	25.8324	131.266	Sota et al., 2015
Aedes togo	25.2075	121.6908	Sota et al., 2015
Aedes togo	27.05475	142.2158	Bohart, 1956
Aedes togo	9.504725	99.99325	Tanaka et al., 1975
Aedes togo	43.79429	146.7332	Shestakov and lukyanchuk, 1980
Aedes togo	43.70845	135.2819	Petrischeva, 1948
Aedes togo	42.47045	130.9226	Petrischeva, 1948
Aedes togo	42.64722	131.0788	Petrischeva, 1948
Aedes togo	42.59372	130.9208	Petrischeva, 1948
Aedes togo	41.78316	123.4282	Petrischeva, 1948
Aedes togo	43.61667	122.2667	Petrischeva, 1948
Aedes togo	38.80869	121.146	Petrischeva, 1948
Aedes togo	42.25473	130.2981	Petrischeva, 1948
Aedes togo	41.77549	129.7728	Petrischeva, 1948
Aedes togo	45.04787	136.6209	Sazanova and Smirnova, 1986
Aedes togo	39.91667	116.3833	Petrischeva, 1948
Aedes togo	27.07972	142.213	Tanaka et al., 1975
Aedes togo	24.28793	153.981	Tanaka et al., 1975
Aedes togo	46.95951	142.7325	Sazanova and Smirnova, 1986
Aedes togo	42.42706	130.6462	Sazanova and Smirnova, 1986
Aedes togo	42.79687	131.2467	Sazanova and Smirnova, 1986
Aedes togo	42.9617	131.7262	Sazanova and Smirnova, 1986
Aedes togo	42.87782	133.7941	Sazanova and Smirnova, 1986
Aedes togo	44.3214	132.0856	Sazanova and Smirnova, 1986
Aedes togo	47.00643	142.0426	Sazanova and Smirnova, 1986
Aedes togo	46.62532	143.5157	Sazanova and Smirnova, 1986
Aedes togo	46.71435	142.5237	Sazanova and Smirnova, 1986

Aedes togo	44.14909	145.8742	Sazanova and Smirnova, 1986
Aedes togo	43.11632	131.8822	Sazanova and Smirnova, 1986
Aedes togo	34.65	127.75	Lee and Hong, 1995
Aedes togo	38.20779	128.5851	Lee and Hong, 1995
Aedes togo	4.8	103.4	Sota, 1994
Aedes togo	5.3	103.1	Sota, 1994
Aedes togo	33.5	131	Sota, 1994
Aedes togo	39.6	142	Sota, 1994
Aedes togo	45	142.5	Sota, 1994
Aedes togo	45.5	141.9	Sota, 1994
Aedes togo	33.50981	126.5237	Kim and Seo, 1968
Aedes togo	22.75833	121.1444	Lien, 1962
Aedes togo	22.67549	120.4914	Lien, 1962
Aedes togo	25.23611	121.6175	Lien, 1962
Aedes togo	22.05	121.5333	Lien, 1962
Aedes togo	34.64652	125.4097	Cheun, 2011
Aedes togo	34.64395	125.3976	Cheun, 2011
Aedes togo	36.00068	127.1673	Cheun, 2011
Aedes togo	34.63932	128.5755	Cheun, 2011
Aedes togo	34.0226	127.304	Cheun, 2011
Aedes togo	33.27568	126.6663	Cheun, 2011
Aedes togo	5.311667	103.12	Ramalingam, 1969
Aedes togo	4.418613	103.4467	Ramalingam, 1969
Aedes togo	4.127022	103.395	Ramalingam, 1969
Aedes togo	3.991407	103.4115	Ramalingam, 1969
Aedes togo	11.9173	109.1444	Ramalingam, 1969
Aedes togo	8.699518	106.6076	Ramalingam, 1969
Aedes togo	9.83052	98.45028	Ramalingam, 1969
Aedes togo	9.52022	100.0314	Ramalingam, 1969
Aedes togo	9.139722	99.33056	Ramalingam, 1969
Aedes togo	12.50755	102.1035	Ramalingam, 1969
Aedes togo	33.1095	139.7909	Omori, 1962

SPECIES	Bio_01.asc	Bio_02.asc	Bio_03.asc	Bio_04.asc	Bio_05.asc	Bio_06.asc	Bio_07.asc	Bio_08.asc
Bio_01.asc	1	-0.67061	0.656703	-0.8361	0.772562	0.974729	-0.85654	0.815427
Bio_02.asc	1	1	-0.27019	0.555295	-0.42522	-0.71442	0.685464	-0.51399
Bio_03.asc	1	1	1	-0.83597	0.216487	0.73707	-0.79806	0.311108
Bio_04.asc	1	1	1	1	-0.31452	-0.92436	0.98326	-0.39846
Bio_05.asc	1	1	1	1	1	0.624221	-0.34193	0.94074
Bio_06.asc	1	1	1	1	1	1	-0.9476	0.688808
Bio_07.asc	1	1	1	1	1	1	1	-0.44385
Bio_08.asc	1	1	1	1	1	1	1	1
Bio_09.asc	1	1	1	1	1	1	1	1
Bio_10.asc	1	1	1	1	1	1	1	1
Bio_11.asc	1	1	1	1	1	1	1	1
Bio_12.asc	1	1	1	1	1	1	1	1
Bio_13.asc	1	1	1	1	1	1	1	1
Bio_14.asc	1	1	1	1	1	1	1	1
Bio_15.asc	1	1	1	1	1	1	1	1
Bio_16.asc	1	1	1	1	1	1	1	1
Bio_17.asc	1	1	1	1	1	1	1	1
Bio_18.asc	1	1	1	1	1	1	1	1
Bio_19.asc	1	1	1	1	1	1	1	1

Bio_09.asc	Bio_10.asc	Bio_11.asc	Bio_12.asc	Bio_13.asc	Bio_14.asc	Bio_15.asc	Bio_16.asc	Bio_17.asc
0.971985	0.853815	0.97449	0.774465	0.724029	0.460223	-0.55432	0.731082	0.490525
-0.65572	-0.58728	-0.65522	-0.70039	-0.60277	-0.51817	0.721692	-0.60935	-0.53969
0.74507	0.288466	0.755585	0.714321	0.539016	0.647911	-0.49808	0.563337	0.66453
-0.93085	-0.42976	-0.93748	-0.79776	-0.70878	-0.50299	0.586275	-0.73391	-0.5302
0.623266	0.975413	0.618328	0.392005	0.400773	0.191846	-0.25252	0.387337	0.21243
0.992613	0.731746	0.995545	0.83151	0.745749	0.536566	-0.63738	0.760434	0.566499
-0.9391	-0.48133	-0.94465	-0.83989	-0.73314	-0.56696	0.663411	-0.7563	-0.59455
0.673661	0.962995	0.678631	0.476074	0.482785	0.25953	-0.28894	0.468229	0.279639
1	0.719533	0.996129	0.815972	0.739792	0.508249	-0.60782	0.755477	0.538365
1	1	0.716988	0.520598	0.514611	0.292903	-0.37137	0.502315	0.316751
1	1	1	0.816951	0.746199	0.501159	-0.5957	0.760965	0.531361
1	1	1	1	0.895286	0.731835	-0.68252	0.915264	0.756398
1	1	1	1	1	0.405273	-0.37187	0.991531	0.433705
1	1	1	1	1	1	-0.79853	0.426282	0.996081
1	1	1	1	1	1	1	-0.40537	-0.8125
1	1	1	1	1	1	1	1	0.454958
1	1	1	1	1	1	1	1	1
1	1	1	1	1	1	1	1	1
1	1	1	1	1	1	1	1	1

Bio_18.asc	Bio_19.asc
0.54509	0.501701
-0.61219	-0.4814
0.37107	0.709818
-0.56048	-0.54587
0.195431	0.227617
0.584133	0.573685
-0.62271	-0.59698
0.350372	0.28446
0.563854	0.551723
0.353659	0.31925
0.571476	0.544056
0.77067	0.735699
0.781165	0.456743
0.457126	0.913424
-0.45088	-0.72862
0.78683	0.472425
0.476287	0.922939
1	0.390376
1	1

SPECIES	Bio 01.asc	Bio 02.asc	Bio 03.asc	Bio 04.asc	Bio 05.asc	Bio 06.asc	Bio 07.asc	Bio 08.asc
Bio 01.asc	1	0.256861	0.819893	-0.86177	0.840268	0.963	-0.794	0.729325
Bio 02.asc	1	1	0.321703	-0.06455	0.523757	0.098052	0.150661	0.263423
Bio 03.asc	1	1	1	-0.8344	0.607393	0.843424	-0.76244	0.489841
Bio 04.asc	1	1	1	1	-0.47828	-0.95409	0.973548	-0.37096
Bio 05.asc	1	1	1	1	1	0.685434	-0.3531	0.814061
Bio 06.asc	1	1	1	1	1	1	-0.92322	0.575616
Bio 07.asc	1	1	1	1	1	1	1	-0.31007
Bio 08.asc	1	1	1	1	1	1	1	1
Bio 09.asc	1	1	1	1	1	1	1	1
Bio 10.asc	1	1	1	1	1	1	1	1
Bio 11.asc	1	1	1	1	1	1	1	1
Bio 12.asc	1	1	1	1	1	1	1	1
Bio 13.asc	1	1	1	1	1	1	1	1
Bio 14.asc	1	1	1	1	1	1	1	1
Bio 15.asc	1	1	1	1	1	1	1	1
Bio 16.asc	1	1	1	1	1	1	1	1
Bio 17.asc	1	1	1	1	1	1	1	1
Bio 18.asc	1	1	1	1	1	1	1	1
Bio 19.asc	1	1	1	1	1	1	1	1

Bio 09.asc	Bio 10.asc	Bio 11.asc	Bio 12.asc	Bio 13.asc	Bio 14.asc	Bio 15.asc	Bio 16.asc	Bio 17.asc
0.919432	0.906918	0.976017	0.650472	0.669213	0.341988	0.156753	0.663839	0.371701
0.200025	0.352292	0.174553	-0.2428	-0.15094	-0.28169	0.331429	-0.16268	-0.28147
0.823344	0.637747	0.854276	0.650975	0.590307	0.492107	0.052416	0.59859	0.514118
-0.9119	-0.57085	-0.94978	-0.65311	-0.61795	-0.42262	0.029714	-0.6235	-0.45136
0.687656	0.970575	0.718596	0.386022	0.44179	0.123331	0.235039	0.430299	0.145485
0.952547	0.776308	0.995556	0.700386	0.680866	0.427364	0.031175	0.681611	0.458063
-0.86109	-0.48536	-0.90003	-0.69623	-0.64164	-0.48351	0.084393	-0.64874	-0.51162
0.489878	0.870306	0.603198	0.420162	0.511367	0.120301	0.400525	0.491807	0.135623
1	0.74206	0.951827	0.620519	0.591107	0.38999	-0.05419	0.593311	0.421448
1	1	0.798575	0.50868	0.563411	0.20737	0.258039	0.550127	0.231586
1	1	1	0.673461	0.668116	0.389534	0.073552	0.667266	0.419817
1	1	1	1	0.924036	0.730504	-0.08959	0.939758	0.759404
1	1	1	1	1	0.460843	0.187046	0.993909	0.492546
1	1	1	1	1	1	-0.4563	0.479751	0.994417
1	1	1	1	1	1	1	0.151133	-0.4566
1	1	1	1	1	1	1	1	0.51233
1	1	1	1	1	1	1	1	1
1	1	1	1	1	1	1	1	1
1	1	1	1	1	1	1	1	1

Bio 18.asc	Bio 19.asc
0.575905	0.378637
-0.14329	-0.20994
0.457839	0.542391
-0.48738	-0.4781
0.379766	0.158412
0.557264	0.4745
-0.51561	-0.52599
0.543676	0.054261
0.449051	0.471919
0.511707	0.222571
0.551634	0.436257
0.806583	0.731942
0.850478	0.512612
0.46031	0.842117
0.186315	-0.36238
0.847022	0.534534
0.480553	0.861589
1	0.341417
1	1

Variables	Regularizat	Log Likelihc	Parameters	Sample Size	AIC score	AICc score
Bio 02, 03, 08, 11, 13, 17, 18	1	-1241.3	51	86	2584.61	2740.61
Bio 02, 03, 05, 11, 13, 17, 18	1	-1236.86	59	86	2591.712	2864.019
Bio 02, 03, 10, 11, 13, 17, 18	1	-1236.08	61	86	2594.155	2909.321
Bio 02, 03, 08, 11, 13, 14, 18	1	-1243.44	56	86	2598.872	2819.01
Bio 02, 03, 05, 11, 13, 14, 18	1	-1237.33	55	86	2584.667	2790
Bio 02, 03, 11, 13, 14, 18	1	-1236.03	59	86	2590.058	2862.366
Bio 02, 03, 08, 11, 13, 14, 18, 19	1	-1250.77	60	86	2621.538	2914.338
Bio 02, 03, 10, 11, 13, 18, 19	1	-1241.79	53	86	2589.583	2768.458
Bio 02, 03, 10, 11, 13, 18, 19	1	-1241.75	56	86	2595.505	2815.643
Bio 02, 03, 08, 11, 16, 17, 18	1	-1244.97	65	86	2619.936	3048.936
Bio 02, 03, 05, 11, 16, 17, 18	1	-1236.97	63	86	2599.933	2966.478
Bio 02, 03, 10, 11, 16, 17, 18	1	-1236.35	56	86	2584.708	2804.846
Bio 02, 03, 08, 11, 14, 16, 18	1	-1245.04	60	86	2610.086	2902.886
Bio 02, 03, 05, 11, 14, 16, 18	1	-1238.3	55	86	2586.603	2791.936
Bio 02, 03, 10, 11, 14, 16, 18	1	-1237.68	58	86	2591.359	2844.841
Bio 02, 03, 08, 11, 16, 18, 19	1	-1250.31	56	86	2612.615	2832.753
Bio 02, 03, 05, 11, 16, 18, 19	1	-1241.61	47	86	2577.22	2695.957
Bio 02, 03, 10, 11, 16, 18, 19	1	-1241.42	48	86	2578.84	2705.975
Bio 02, 03, 06, 08, 13, 17, 18	1	-1239.66	58	86	2595.315	2848.796
Bio 02, 03, 05, 06, 13, 17, 18	1	-1234.13	49	86	2566.25	2702.361
Bio 02, 03, 06, 10, 13, 17, 18	1	-1231.83	55	86	2573.651	2778.984
Bio 02, 03, 06, 08, 13, 14, 18	1	-1240.1	52	86	2584.204	2751.235
Bio 02, 03, 05, 06, 13, 14, 18	1	-1234.93	51	86	2571.863	2727.863
Bio 02, 03, 06, 10, 13, 14, 18	1	-1232.42	56	86	2576.85	2796.988
Bio 02, 06, 08, 13, 18, 19	1	-1257.3	48	86	2610.601	2737.736
Bio 02, 05, 06, 13, 18, 19	1	-1246.29	34	86	2560.584	2607.251
Bio 02, 06, 10, 13, 18, 19	1	-1244.57	37	86	2563.147	2621.73
Bio 02, 06, 08, 16, 17, 18	1	-1256.17	50	86	2612.333	2758.047
Bio 02, 05, 06, 16, 17, 18	1	-1243.94	42	86	2571.878	2655.878
Bio 02, 06, 10, 16, 17, 18	1	-1243.85	48	86	2583.697	2710.832
Bio 02, 06, 08, 14, 16, 18	1	-1255.59	49	86	2609.186	2745.297
Bio 02, 05, 06, 14, 16, 18	1	-1246.43	44	86	2580.865	2677.45
Bio 02, 06, 10, 14, 16, 18	1	-1243.4	44	86	2574.803	2671.389
Bio 02, 06, 08, 16, 18, 19	1	-1259.35	50	86	2618.705	2764.42
Bio 02, 05, 06, 16, 18, 19	1	-1248.5	45	86	2586.999	2690.499
Bio 02, 06, 10, 16, 18, 19	1	-1250.71	39	86	2579.422	2647.248
Bio 02, 04, 07, 08, 09, 12, 13, 18, 19	1	-1238.62	64	86	2605.249	3001.44
Bio 02, 04, 07, 08, 09, 12, 16, 18, 19	1	-1238.65	63	86	2603.292	2969.838
Bio 02, 04, 07, 08, 09, 12, 13, 17, 18	1	-1233.97	67	86	2601.93	3108.152
Bio 02, 04, 07, 08, 09, 16, 17, 18	1	-1235.2	65	86	2600.393	3029.393
Bio 02, 04, 07, 08, 09, 12, 13, 14, 18	1	-1234.61	63	86	2595.227	2961.773
Bio 02, 04, 07, 08, 09, 12, 14, 16, 18	1	-1236.55	73	86	2619.098	3519.431
Bio 02, 03, 08, 11, 13, 17, 18	1	-1241.98	57	86	2597.951	2834.094
Bio 02, 03, 05, 11, 13, 17, 18	1	-1236.33	62	86	2596.651	2936.303
Bio 02, 03, 10, 11, 13, 17, 18	1	-1236.3	62	86	2596.606	2936.258
Bio 02, 03, 08, 11, 13, 14, 18	1	-1242.92	58	86	2601.841	2855.323
Bio 02, 03, 05, 11, 13, 14, 18	1	-1237.18	63	86	2600.356	2966.901
Bio 02, 03, 11, 13, 14, 18	1	-1234.85	59	86	2587.692	2859.999
Bio 02, 03, 08, 11, 13, 14, 18, 19	1	-1240.49	60	86	2600.972	2893.772
Bio 02, 03, 10, 11, 13, 18, 19	1	-1242.11	49	86	2582.229	2718.34
Bio 02, 03, 10, 11, 13, 18, 19	1	-1242.11	49	86	2582.229	2718.34

Bio 02, 03, 08, 11, 16, 17, 18	1	-1241.42	57	86	2596.845	2832.988
Bio 02, 03, 05, 11, 16, 17, 18	1	-1236.59	64	86	2601.19	2997.38
Bio 02, 03, 10, 11, 16, 17, 18	1	-1236.91	53	86	2579.825	2758.7
Bio 02, 03, 08, 11, 14, 16, 18	1	-1244.41	63	86	2614.814	2981.36
Bio 02, 03, 05, 11, 14, 16, 18	1	-1239.02	56	86	2590.044	2810.182
Bio 02, 03, 10, 11, 14, 16, 18	1	-1237.1	64	86	2602.208	2998.398
Bio 02, 03, 08, 11, 16, 18, 19	1	-1250.8	55	86	2611.599	2816.932
Bio 02, 03, 05, 11, 16, 18, 19	1	-1241.5	52	86	2586.997	2754.028
Bio 02, 03, 10, 11, 16, 18, 19	1	-1239.96	57	86	2593.925	2830.068
Bio 02, 03, 06, 08, 13, 17, 18	1	-1240.44	60	86	2600.89	2893.69
Bio 02, 03, 05, 06, 13, 17, 18	1	-1233.45	58	86	2582.891	2836.372
Bio 02, 03, 06, 10, 13, 17, 18	1	-1233.31	62	86	2590.626	2930.279
Bio 02, 03, 06, 08, 13, 14, 18	1	-1242.75	65	86	2615.505	3044.505
Bio 02, 03, 05, 06, 13, 14, 18	1	-1233.48	60	86	2586.964	2879.764
Bio 02, 03, 06, 10, 13, 14, 18	1	-1233.36	63	86	2592.725	2959.271
Bio 02, 06, 08, 13, 18, 19	1	-1257.7	46	86	2607.405	2718.277
Bio 02, 05, 06, 13, 18, 19	1	-1247.43	42	86	2578.865	2662.865
Bio 02, 06, 10, 13, 18, 19	1	-1244.72	53	86	2595.449	2774.324
Bio 02, 06, 08, 16, 17, 18	1	-1257.42	49	86	2612.834	2748.945
Bio 02, 05, 06, 16, 17, 18	1	-1245.33	47	86	2584.667	2703.404
Bio 02, 06, 10, 16, 17, 18	1	-1242.65	44	86	2573.309	2669.895
Bio 02, 06, 08, 14, 16, 18	1	-1256.59	48	86	2609.186	2736.321
Bio 02, 05, 06, 14, 16, 18	1	-1246.25	45	86	2582.498	2685.998
Bio 02, 06, 10, 14, 16, 18	1	-1243.71	49	86	2585.428	2721.539
Bio 02, 06, 08, 16, 18, 19	1	-1261.88	50	86	2623.761	2769.475
Bio 02, 05, 06, 16, 18, 19	1	-1249.4	49	86	2596.802	2732.913
Bio 02, 06, 10, 16, 18, 19	1	-1246.53	49	86	2591.062	2727.173
Bio 02, 04, 07, 08, 09, 12, 13, 18, 19	1	-1239.01	70	86	2618.014	3280.681
Bio 02, 04, 07, 08, 09, 12, 16, 18, 19	1	-1238.53	65	86	2607.07	3036.07
Bio 02, 04, 07, 08, 09, 12, 13, 17, 18	1	-1234.12	65	86	2598.247	3027.247
Bio 02, 04, 07, 08, 09, 16, 17, 18	1	-1235.39	63	86	2596.776	2963.321
Bio 02, 04, 07, 08, 09, 12, 13, 14, 18	1	-1234.93	61	86	2591.863	2907.03
Bio 02, 04, 07, 08, 09, 12, 14, 16, 18	1	-1236.9	63	86	2599.797	2966.342
Bio 02, 03, 08, 11, 13, 17, 18	1	-1242.19	55	86	2594.377	2799.71
Bio 02, 03, 05, 11, 13, 17, 18	1	-1237.88	57	86	2589.76	2825.903
Bio 02, 03, 10, 11, 13, 17, 18	1	-1235.08	63	86	2596.159	2962.705
Bio 02, 03, 08, 11, 13, 14, 18	1	-1244.32	55	86	2598.638	2803.971
Bio 02, 03, 05, 11, 13, 14, 18	1	-1237.01	50	86	2574.017	2719.731
Bio 02, 03, 11, 13, 14, 18	1	-1234.79	58	86	2585.588	2839.07
Bio 02, 03, 08, 11, 13, 14, 18, 19	1	-1250.59	54	86	2609.187	2800.8
Bio 02, 03, 10, 11, 13, 18, 19	1	-1241.69	51	86	2585.387	2741.387
Bio 02, 03, 10, 11, 13, 18, 19	1	-1242.89	53	86	2591.77	2770.645
Bio 02, 03, 08, 11, 16, 17, 18	1	-1243.43	53	86	2592.863	2771.738
Bio 02, 03, 05, 11, 16, 17, 18	1	-1236.07	57	86	2586.133	2822.276
Bio 02, 03, 10, 11, 16, 17, 18	1	-1236.52	56	86	2585.05	2805.188
Bio 02, 03, 08, 11, 14, 16, 18	1	-1242.96	56	86	2597.922	2818.06
Bio 02, 03, 05, 11, 14, 16, 18	1	-1238.23	56	86	2588.463	2808.601
Bio 02, 03, 10, 11, 14, 16, 18	1	-1237.88	55	86	2585.762	2791.095
Bio 02, 03, 08, 11, 16, 18, 19	1	-1250.7	58	86	2617.391	2870.872
Bio 02, 03, 05, 11, 16, 18, 19	1	-1242.32	50	86	2584.641	2730.355
Bio 02, 03, 10, 11, 16, 18, 19	1	-1242.12	53	86	2590.237	2769.112
Bio 02, 03, 06, 08, 13, 17, 18	1	-1240.2	58	86	2596.4	2849.882

Bio 02, 03, 05, 06, 13, 17, 18	1	-1234.25	50	86	2568.501	2714.215
Bio 02, 03, 06, 10, 13, 17, 18	1	-1232.74	51	86	2567.473	2723.473
Bio 02, 03, 06, 08, 13, 14, 18	1	-1239.83	54	86	2587.654	2779.267
Bio 02, 03, 05, 06, 13, 14, 18	1	-1235.01	46	86	2562.029	2672.9
Bio 02, 03, 06, 10, 13, 14, 18	1	-1232.24	48	86	2560.486	2687.622
Bio 02, 06, 08, 13, 18, 19	1	-1256.24	41	86	2594.481	2672.753
Bio 02, 05, 06, 13, 18, 19	1	-1246.46	36	86	2564.926	2619.293
Bio 02, 06, 10, 13, 18, 19	0.00001	-1177.49	266	86	x	x
Bio 02, 06, 10, 13, 18, 19	0.0001	-1167.97	251	86	x	x
Bio 02, 06, 10, 13, 18, 19	0.001	-1169.24	271	86	x	x
Bio 02, 06, 10, 13, 18, 19	0.01	-1174.08	229	86	x	x
Bio 02, 06, 10, 13, 18, 19	0.1	-1198.92	145	86	x	x
Bio 02, 06, 10, 13, 18, 19	0.5	-1232.7	71	86	2607.402	3337.688
Bio 02, 06, 10, 13, 18, 19	10	-1324.9	6	86	2661.8	2662.863
Bio 02, 06, 10, 13, 18, 19	100	-1394.9	1	86	2791.795	2791.843
Bio 02, 06, 10, 13, 18, 19	2	-1264.58	25	86	2579.161	2600.828
Bio 02, 06, 10, 13, 18, 19	1	-1245.57	33	86	2557.142	2600.296
Bio 02, 06, 08, 16, 17, 18	1	-1254.97	46	86	2601.936	2712.808
Bio 02, 05, 06, 16, 17, 18	1	-1244.72	48	86	2585.444	2712.579
Bio 02, 06, 10, 16, 17, 18	1	-1242.67	42	86	2569.34	2653.34
Bio 02, 06, 08, 14, 16, 18	1	-1254.44	49	86	2606.89	2743.001
Bio 02, 05, 06, 14, 16, 18	1	-1245.8	45	86	2581.603	2685.103
Bio 02, 06, 10, 14, 16, 18	1	-1242.21	47	86	2578.426	2697.163
Bio 02, 06, 08, 16, 18, 19	1	-1259.6	48	86	2615.204	2742.339
Bio 02, 05, 06, 16, 18, 19	1	-1247.83	43	86	2581.665	2671.76
Bio 02, 06, 10, 16, 18, 19	1	-1244.74	36	86	2561.471	2615.838
Bio 02, 04, 07, 08, 09, 12, 13, 18, 19	1	-1239.12	56	86	2590.239	2810.377
Bio 02, 04, 07, 08, 09, 12, 16, 18, 19	1	-1239.06	62	86	2602.115	2941.767
Bio 02, 04, 07, 08, 09, 12, 13, 17, 18	1	-1233.59	60	86	2587.175	2879.975
Bio 02, 04, 07, 08, 09, 16, 17, 18	1	-1234.14	63	86	2594.276	2960.822
Bio 02, 04, 07, 08, 09, 12, 13, 14, 18	1	-1233.52	57	86	2581.04	2817.183
Bio 02, 04, 07, 08, 09, 12, 14, 16, 18	1	-1235.04	67	86	2604.071	3110.294

BIC score
2709.781
2736.518
2743.87
2736.316
2719.656
2734.865
2768.799
2719.663
2732.948
2779.468
2754.557
2722.152
2757.347
2721.592
2733.711
2750.058
2692.574
2696.649
2737.667
2686.513
2708.64
2711.83
2697.035
2714.293
2728.41
2644.032
2653.958
2735.05
2674.96
2701.505
2729.449
2688.856
2682.795
2741.423
2697.445
2675.142
2762.327
2757.916
2766.372
2759.925
2749.851
2798.265
2737.849
2748.82
2748.776
2744.194
2754.98
2732.498
2748.233
2702.492
2702.492

2736.743
2758.268
2709.905
2769.438
2727.488
2759.286
2746.588
2714.623
2733.823
2748.15
2725.243
2742.796
2775.037
2734.225
2747.349
2720.305
2681.947
2725.529
2733.097
2700.022
2681.301
2726.995
2692.943
2705.691
2746.478
2717.065
2711.325
2789.819
2766.602
2757.78
2751.399
2741.579
2754.421
2729.366
2729.658
2750.783
2733.627
2696.735
2727.94
2741.722
2710.559
2721.851
2722.943
2726.031
2722.493
2735.365
2725.906
2720.751
2759.743
2707.358
2720.318
2738.753

2691.219
2692.645
2720.189
2674.929
2678.295
2695.109
2653.282
x
x
x
x
x
2781.661
2676.526
2794.249
2640.52
2638.136
2714.836
2703.253
2672.423
2727.153
2692.049
2693.781
2733.013
2687.202
2649.827
2727.683
2754.285
2734.436
2748.9
2720.938
2768.513

Variables	Regularizat	Log Likelihc	Parameters	Sample Size	AIC score	AICc score	BIC score
02, 15, 05, 14, 13, 11	1	-1994.44	50	135	4088.871	4149.585	4234.134
02, 15, 08, 14, 13, 11	1	-2011.37	46	135	4114.731	4163.868	4248.374
02, 15, 10, 14, 13, 11	1	-1977.35	43	135	4040.704	4082.287	4165.631
02, 15, 05, 17, 13, 11	1	-1992.77	51	135	4087.542	4151.446	4235.711
02, 15, 08, 17, 13, 11	1	-2021.05	53	135	4148.094	4218.76	4302.073
02, 15, 10, 17, 13, 11	1	-1977.12	46	135	4046.234	4095.37	4179.877
02, 15, 05, 19, 13, 11	1	-1992.41	50	135	4084.817	4145.532	4230.081
02, 15, 08, 19, 13, 11	1	-2023.3	53	135	4152.591	4223.258	4306.571
02, 15, 10, 19, 13, 11	1	-1982	50	135	4063.995	4124.709	4209.259
02, 15, 05, 14, 16, 11	1	-1989.1	52	135	4082.198	4149.417	4233.272
02, 15, 08, 14, 16, 11	1	-2011.22	51	135	4124.431	4188.334	4272.6
02, 15, 10, 14, 16, 11	1	-1972.87	44	135	4033.746	4077.746	4161.578
02, 15, 05, 17, 16, 11	1	-1985.59	45	135	4061.188	4107.705	4191.925
02, 15, 08, 17, 16, 11	1	-2012.35	44	135	4112.703	4156.703	4240.536
02, 15, 10, 17, 16, 11	1	-1964.55	46	135	4021.102	4070.238	4154.745
02, 15, 05, 19, 16, 11	1	-1989.83	47	135	4073.652	4125.515	4210.2
02, 15, 08, 19, 16, 11	1	-2026.85	53	135	4159.708	4230.375	4313.688
02, 15, 10, 19, 16, 11	1	-1979.26	55	135	4068.526	4146.501	4228.316
02, 15, 05, 14, 18, 11	1	-1974.08	57	135	4062.156	4148.026	4227.757
02, 15, 08, 14, 18, 11	1	-1999.35	49	135	4096.691	4154.338	4239.049
02, 15, 10, 14, 18, 11	1	-1960.14	51	135	4022.279	4086.182	4170.448
02, 15, 05, 17, 18, 11	1	-1973.92	64	135	4075.837	4194.694	4261.775
02, 15, 08, 17, 18, 11	1	-2005.06	52	135	4114.111	4181.33	4265.185
02, 15, 10, 17, 18, 11	1	-1958.55	59	135	4035.108	4129.508	4206.519
02, 15, 05, 19, 18, 11	1	-1986.34	61	135	4094.672	4198.289	4271.894
02, 15, 08, 19, 18, 11	1	-2017.29	57	135	4148.584	4234.454	4314.184
02, 15, 10, 19, 18, 11	1	-1976.77	57	135	4067.549	4153.419	4233.149
02, 15, 05, 14, 12, 11	1	-1994.68	53	135	4095.35	4166.017	4249.33
02, 15, 08, 14, 12, 11	1	-2020.27	48	135	4136.55	4191.247	4276.003
02, 15, 10, 14, 12, 11	1	-1971.12	48	135	4038.241	4092.939	4177.694
02, 15, 05, 17, 12, 11	1	-1990.03	47	135	4074.056	4125.918	4210.604
02, 15, 08, 17, 12, 11	1	-2025.39	45	135	4140.778	4187.295	4271.515
02, 15, 10, 17, 12, 11	1	-1970.27	47	135	4034.541	4086.403	4171.089
02, 15, 05, 19, 12, 11	1	-1987.67	56	135	4087.349	4169.195	4250.045
02, 15, 08, 19, 12, 11	1	-2030.43	53	135	4166.861	4237.528	4320.841
02, 15, 10, 19, 12, 11	1	-1973.76	54	135	4055.513	4129.763	4212.398
02, 15, 05, 14, 13, 06	1	-1998.48	50	135	4096.966	4157.681	4242.23
02, 15, 08, 14, 13, 06	1	-2007.04	46	135	4106.09	4155.226	4239.732
02, 15, 10, 14, 13, 06	1	-1977.35	48	135	4050.693	4105.391	4190.147
02, 15, 05, 17, 13, 06	1	-1991.67	49	135	4081.334	4138.981	4223.693
02, 15, 08, 17, 13, 06	1	-2010.56	47	135	4115.118	4166.98	4251.666
02, 15, 10, 17, 13, 06	1	-1974.81	46	135	4041.627	4090.763	4175.269
02, 15, 05, 19, 13, 06	1	-1993.13	45	135	4076.261	4122.778	4206.999
02, 15, 08, 19, 13, 06	1	-2017.8	59	135	4153.608	4248.008	4325.019
02, 15, 10, 19, 13, 06	1	-1980.6	52	135	4065.209	4132.429	4216.283
02, 15, 05, 14, 16, 06	1	-1991.01	43	135	4068.018	4109.601	4192.945
02, 15, 08, 14, 16, 06	1	-1992.77	47	135	4079.542	4131.404	4216.09
02, 15, 10, 14, 16, 06	1	-1967.99	41	135	4017.988	4055.021	4137.105
02, 15, 05, 17, 16, 06	1	-1985.54	47	135	4065.077	4116.939	4201.625
02, 15, 08, 17, 16, 06	1	-1989.77	49	135	4077.546	4135.193	4219.905
02, 15, 10, 17, 16, 06	1	-1967.92	41	135	4017.849	4054.882	4136.966

02, 15, 10, 17, 16, 06	0.001	-2042.14	258	135	x	x	x
02, 15, 10, 17, 16, 06	0.01	-1989.82	220	135	x	x	x
02, 15, 10, 17, 16, 06	0.1	-1899.79	146	135	x	x	x
02, 15, 10, 17, 16, 06	0.5	-1948.09	71	135	4038.174	4200.46	4244.449
02, 15, 10, 17, 16, 06	2	-1990.06	31	135	4042.114	4061.376	4132.177
02, 15, 10, 17, 16, 06	5	-2046.22	15	135	4122.44	4126.473	4166.019
02, 15, 05, 19, 16, 06	1	-1994.76	54	135	4097.513	4171.763	4254.398
02, 15, 08, 19, 16, 06	1	-2025.18	58	135	4166.356	4256.408	4334.862
02, 15, 10, 19, 16, 06	1	-1981.97	57	135	4077.936	4163.806	4243.536
02, 15, 05, 14, 18, 06	1	-1972.82	58	135	4061.648	4151.701	4230.154
02, 15, 08, 14, 18, 06	1	-1999.73	52	135	4103.459	4170.679	4254.534
02, 15, 10, 14, 18, 06	1	-1957.33	54	135	4022.659	4096.909	4179.544
02, 15, 05, 17, 18, 06	1	-1969.92	67	135	4073.849	4209.849	4268.502
02, 15, 08, 17, 18, 06	1	-1998.79	52	135	4101.582	4168.802	4252.657
02, 15, 10, 17, 18, 06	1	-1956.28	60	135	4032.566	4131.484	4206.882
02, 15, 05, 19, 18, 06	1	-1987.84	62	135	4099.685	4208.185	4279.812
02, 15, 08, 19, 18, 06	1	-2016.74	63	135	4159.47	4273.048	4342.503
02, 15, 10, 19, 18, 06	1	-1978.17	58	135	4072.347	4162.399	4240.853
02, 15, 05, 14, 12, 06	1	-1990.51	52	135	4085.023	4152.243	4236.097
02, 15, 08, 14, 12, 06	1	-2009.71	47	135	4113.417	4165.279	4249.965
02, 15, 10, 14, 12, 06	1	-1974.79	50	135	4049.573	4110.287	4194.837
02, 15, 05, 17, 12, 06	1	-1988.65	53	135	4083.296	4153.962	4237.275
02, 15, 08, 17, 12, 06	1	-2014.57	44	135	4117.148	4161.148	4244.98
02, 15, 10, 17, 12, 06	1	-1970.27	47	135	4034.541	4086.403	4171.089
02, 15, 05, 19, 12, 06	1	-1994.41	52	135	4092.817	4160.037	4243.892
02, 15, 08, 19, 12, 06	1	-2035.43	54	135	4178.862	4253.112	4335.747
02, 15, 10, 19, 12, 06	1	-1980.27	56	135	4072.544	4154.39	4235.239
02, 15, 05, 14, 13, 09	1	-1991.85	57	135	4097.701	4183.571	4263.302
02, 15, 08, 14, 13, 09	1	-1988.33	53	135	4082.66	4153.326	4236.639
02, 15, 10, 14, 13, 09	1	-1976.5	41	135	4034.995	4072.027	4154.111
02, 15, 05, 17, 13, 09	1	-1993.49	47	135	4080.979	4132.841	4217.527
02, 15, 08, 17, 13, 09	1	-1988.72	49	135	4075.441	4133.088	4217.8
02, 15, 10, 17, 13, 09	1	-1974	51	135	4049.995	4113.899	4198.164
02, 15, 05, 19, 13, 09	1	-1993.95	53	135	4093.89	4164.557	4247.87
02, 15, 08, 19, 13, 09	1	-1990.24	59	135	4098.471	4192.871	4269.882
02, 15, 10, 19, 13, 09	1	-1985.81	48	135	4067.628	4122.326	4207.081
02, 15, 05, 14, 16, 09	1	-1988.15	54	135	4084.305	4158.555	4241.19
02, 15, 08, 14, 16, 09	1	-1980.06	62	135	4084.115	4192.615	4264.242
02, 15, 10, 14, 16, 09	1	-1969.05	43	135	4024.091	4065.674	4149.018
02, 15, 05, 17, 16, 09	1	-1987.82	51	135	4077.643	4141.547	4225.812
02, 15, 08, 17, 16, 09	1	-1979.44	56	135	4070.888	4152.734	4233.583
02, 15, 10, 17, 16, 09	1	-1969.53	46	135	4031.058	4080.194	4164.7
02, 15, 05, 19, 16, 09	1	-1994.76	63	135	4115.524	4229.101	4298.556
02, 15, 08, 19, 16, 09	1	-1994.17	60	135	4108.341	4207.26	4282.658
02, 15, 10, 19, 16, 09	1	-1985.1	49	135	4068.208	4125.855	4210.567
02, 15, 05, 14, 18, 09	1	-1980.52	52	135	4065.046	4132.266	4216.121
02, 15, 08, 14, 18, 09	1	-1994.77	53	135	4095.545	4166.212	4249.524
02, 15, 10, 14, 18, 09	1	-1963.28	56	135	4038.559	4120.405	4201.255
02, 15, 05, 17, 18, 09	1	-1982.71	57	135	4079.413	4165.283	4245.014
02, 15, 08, 17, 18, 09	1	-1988.49	53	135	4082.979	4153.645	4236.958
02, 15, 10, 17, 18, 09	1	-1964.8	56	135	4041.604	4123.45	4204.3
02, 15, 05, 19, 18, 09	1	-1990.66	62	135	4105.319	4213.819	4285.446

02, 15, 08, 19, 18, 09	1	-1994.74	60	135	4109.477	4208.395	4283.793
02, 15, 10, 19, 18, 09	1	-1977.51	53	135	4061.021	4131.687	4215
02, 15, 05, 14, 12, 09	1	-1989.81	59	135	4097.627	4192.027	4269.038
02, 15, 08, 14, 12, 09	1	-1988.95	59	135	4095.892	4190.292	4267.304
02, 15, 10, 14, 12, 09	1	-1973.12	46	135	4038.239	4087.375	4171.881
02, 15, 05, 17, 12, 09	1	-1989.75	54	135	4087.5	4161.75	4244.385
02, 15, 08, 17, 12, 09	1	-1985.21	57	135	4084.416	4170.286	4250.017
02, 15, 10, 17, 12, 09	1	-1969.12	47	135	4032.242	4084.104	4168.79
02, 15, 05, 19, 12, 09	1	-1990.34	66	135	4112.687	4242.746	4304.435
02, 15, 08, 19, 12, 09	1	-1996.94	61	135	4115.882	4219.499	4293.104
02, 15, 10, 19, 12, 09	1	-1978.78	52	135	4061.559	4128.779	4212.634
02, 08, 11, 12, 15	1	-2032.55	52	135	4169.093	4236.312	4320.167
02, 06, 10, 13, 18, 19	1	-1972.62	56	135	4057.244	4139.09	4219.939

Points	ASCII file	Variables	Regularizat	Log Likelihc	Parameters	Sample Size	AIC score	AICc score
E:\Beringia	E:\Beringia	02, 15, 05, 14, 13, 11		-1995.58	51	135	4093.16	4157.063
E:\Beringia	E:\Beringia	02, 15, 08, 14, 13, 11		-2012.38	60	135	4144.761	4243.68
E:\Beringia	E:\Beringia	02, 15, 10, 14, 13, 11		-1976.91	39	135	4031.816	4064.658
E:\Beringia	E:\Beringia	02, 15, 05, 17, 13, 11		-1992.92	42	135	4069.848	4109.109
E:\Beringia	E:\Beringia	02, 15, 08, 17, 13, 11		-2017.03	56	135	4146.051	4227.898
E:\Beringia	E:\Beringia	02, 15, 10, 17, 13, 11		-1971.47	50	135	4042.941	4103.655
E:\Beringia	E:\Beringia	02, 15, 05, 19, 13, 11		-1990.95	45	135	4071.909	4118.426
E:\Beringia	E:\Beringia	02, 15, 08, 19, 13, 11		-2030.47	54	135	4168.939	4243.189
E:\Beringia	E:\Beringia	02, 15, 10, 19, 13, 11		-1981.72	45	135	4053.449	4099.966
E:\Beringia	E:\Beringia	02, 15, 05, 14, 16, 11		-1991.61	42	135	4067.226	4106.487
E:\Beringia	E:\Beringia	02, 15, 08, 14, 16, 11		-2006.12	48	135	4108.241	4162.939
E:\Beringia	E:\Beringia	02, 15, 10, 14, 16, 11		-1970.27	46	135	4032.537	4081.673
E:\Beringia	E:\Beringia	02, 15, 05, 17, 16, 11		-1984.95	49	135	4067.907	4125.554
E:\Beringia	E:\Beringia	02, 15, 08, 17, 16, 11		-2004.24	47	135	4102.488	4154.351
E:\Beringia	E:\Beringia	02, 15, 10, 17, 16, 11		-1963.87	44	135	4015.743	4059.743
E:\Beringia	E:\Beringia	02, 15, 05, 19, 16, 11		-1991.22	48	135	4078.437	4133.134
E:\Beringia	E:\Beringia	02, 15, 08, 19, 16, 11		-2050.95	53	135	4207.894	4278.56
E:\Beringia	E:\Beringia	02, 15, 10, 19, 16, 11		-1978.47	54	135	4064.934	4139.184
E:\Beringia	E:\Beringia	02, 15, 05, 14, 18, 11		-1970.29	58	135	4056.588	4146.641
E:\Beringia	E:\Beringia	02, 15, 08, 14, 18, 11		-2003.17	58	135	4122.332	4212.385
E:\Beringia	E:\Beringia	02, 15, 10, 14, 18, 11		-1960.29	55	135	4030.571	4108.546
E:\Beringia	E:\Beringia	02, 15, 05, 17, 18, 11		-1971.58	59	135	4061.153	4155.553
E:\Beringia	E:\Beringia	02, 15, 08, 17, 18, 11		-2004.28	58	135	4124.565	4214.617
E:\Beringia	E:\Beringia	02, 15, 10, 17, 18, 11		-1958.95	51	135	4019.91	4083.813
E:\Beringia	E:\Beringia	02, 15, 05, 19, 18, 11		-1987.36	45	135	4064.716	4111.233
E:\Beringia	E:\Beringia	02, 15, 08, 19, 18, 11		-2020.48	62	135	4164.96	4273.46
E:\Beringia	E:\Beringia	02, 15, 10, 19, 18, 11		-1977.1	51	135	4056.195	4120.099
E:\Beringia	E:\Beringia	02, 15, 05, 14, 12, 11		-1994.71	45	135	4079.418	4125.935
E:\Beringia	E:\Beringia	02, 15, 08, 14, 12, 11		-2012.9	57	135	4139.806	4225.676
E:\Beringia	E:\Beringia	02, 15, 10, 14, 12, 11		-1972.41	48	135	4040.811	4095.509
E:\Beringia	E:\Beringia	02, 15, 05, 17, 12, 11		-1994.93	45	135	4079.852	4126.369
E:\Beringia	E:\Beringia	02, 15, 08, 17, 12, 11		-2017.56	54	135	4143.12	4217.37
E:\Beringia	E:\Beringia	02, 15, 10, 17, 12, 11		-1967.36	44	135	4022.72	4066.72
E:\Beringia	E:\Beringia	02, 15, 05, 19, 12, 11		-1990.61	53	135	4087.224	4157.891
E:\Beringia	E:\Beringia	02, 15, 08, 19, 12, 11		-2043.24	58	135	4202.481	4292.533
E:\Beringia	E:\Beringia	02, 15, 10, 19, 12, 11		-1974.18	55	135	4058.357	4136.332
E:\Beringia	E:\Beringia	02, 15, 05, 14, 13, 06		-1996.56	44	135	4081.11	4125.11
E:\Beringia	E:\Beringia	02, 15, 08, 14, 13, 06		-2000.58	53	135	4107.167	4177.834
E:\Beringia	E:\Beringia	02, 15, 10, 14, 13, 06		-1975.4	47	135	4044.808	4096.67
E:\Beringia	E:\Beringia	02, 15, 05, 17, 13, 06		-1990.48	44	135	4068.953	4112.953
E:\Beringia	E:\Beringia	02, 15, 08, 17, 13, 06		-2006.79	62	135	4137.588	4246.088
E:\Beringia	E:\Beringia	02, 15, 10, 17, 13, 06		-1967.12	51	135	4036.237	4100.141
E:\Beringia	E:\Beringia	02, 15, 05, 19, 13, 06		-1991.94	45	135	4073.882	4120.399
E:\Beringia	E:\Beringia	02, 15, 08, 19, 13, 06		-2016.61	55	135	4143.217	4221.192
E:\Beringia	E:\Beringia	02, 15, 10, 19, 13, 06		-1981.31	47	135	4056.618	4108.48
E:\Beringia	E:\Beringia	02, 15, 05, 14, 16, 06		-1990.4	48	135	4076.801	4131.499
E:\Beringia	E:\Beringia	02, 15, 08, 14, 16, 06		-1992.69	58	135	4101.372	4191.425
E:\Beringia	E:\Beringia	02, 15, 10, 14, 16, 06		-1969.07	37	135	4012.143	4041.133
E:\Beringia	E:\Beringia	02, 15, 05, 17, 16, 06		-1983.1	51	135	4068.193	4132.097
E:\Beringia	E:\Beringia	02, 15, 08, 17, 16, 06		-1999.79	49	135	4097.583	4155.23
E:\Beringia	E:\Beringia	02, 15, 10, 17, 16, 06		-1964.71	42	135	4013.414	4052.675

E:\Beringia	E:\Beringia	02, 15, 05, 19, 16, 06	-1995.86	53	135	4097.722	4168.388
E:\Beringia	E:\Beringia	02, 15, 08, 19, 16, 06	-2028.79	56	135	4169.582	4251.428
E:\Beringia	E:\Beringia	02, 15, 10, 19, 16, 06	-1981.43	52	135	4066.855	4134.074
E:\Beringia	E:\Beringia	02, 15, 05, 14, 18, 06	-1971.84	55	135	4053.675	4131.65
E:\Beringia	E:\Beringia	02, 15, 08, 14, 18, 06	-1996.94	61	135	4115.884	4219.501
E:\Beringia	E:\Beringia	02, 15, 10, 14, 18, 06	-1955.97	50	135	4011.93	4072.644
E:\Beringia	E:\Beringia	02, 15, 05, 17, 18, 06	-1969.02	58	135	4054.033	4144.086
E:\Beringia	E:\Beringia	02, 15, 08, 17, 18, 06	-1996.83	61	135	4115.66	4219.276
E:\Beringia	E:\Beringia	02, 15, 10, 17, 18, 06	-1955.39	52	135	4014.777	4081.997
E:\Beringia	E:\Beringia	02, 15, 05, 19, 18, 06	-1987.45	57	135	4088.896	4174.767
E:\Beringia	E:\Beringia	02, 15, 08, 19, 18, 06	-2018.09	59	135	4154.176	4248.576
E:\Beringia	E:\Beringia	02, 15, 10, 19, 18, 06	-1978.47	54	135	4064.932	4139.182
E:\Beringia	E:\Beringia	02, 15, 05, 14, 12, 06	-1992.92	49	135	4083.848	4141.495
E:\Beringia	E:\Beringia	02, 15, 08, 14, 12, 06	-2006.49	57	135	4126.981	4212.851
E:\Beringia	E:\Beringia	02, 15, 10, 14, 12, 06	-1970.57	47	135	4035.149	4087.011
E:\Beringia	E:\Beringia	02, 15, 05, 17, 12, 06	-1992.7	49	135	4083.392	4141.039
E:\Beringia	E:\Beringia	02, 15, 08, 17, 12, 06	-2007.38	56	135	4126.768	4208.615
E:\Beringia	E:\Beringia	02, 15, 10, 17, 12, 06	-1969.19	45	135	4028.377	4074.894
E:\Beringia	E:\Beringia	02, 15, 05, 19, 12, 06	-2000.48	54	135	4108.969	4183.219
E:\Beringia	E:\Beringia	02, 15, 08, 19, 12, 06	-2028.56	58	135	4173.123	4263.176
E:\Beringia	E:\Beringia	02, 15, 10, 19, 12, 06	-1978.63	52	135	4061.257	4128.476
E:\Beringia	E:\Beringia	02, 15, 05, 14, 13, 09	-1995.95	47	135	4085.897	4137.759
E:\Beringia	E:\Beringia	02, 15, 08, 14, 13, 09	-1985.7	65	135	4101.406	4225.753
E:\Beringia	E:\Beringia	02, 15, 10, 14, 13, 09	-1977.54	54	135	4063.079	4137.329
E:\Beringia	E:\Beringia	02, 15, 05, 17, 13, 09	-1996.53	47	135	4087.063	4138.925
E:\Beringia	E:\Beringia	02, 15, 08, 17, 13, 09	-1982.88	59	135	4083.754	4178.154
E:\Beringia	E:\Beringia	02, 15, 10, 17, 13, 09	-1974.26	44	135	4036.519	4080.519
E:\Beringia	E:\Beringia	02, 15, 05, 19, 13, 09	-1992.18	51	135	4086.35	4150.254
E:\Beringia	E:\Beringia	02, 15, 08, 19, 13, 09	-1988.93	66	135	4109.851	4239.91
E:\Beringia	E:\Beringia	02, 15, 10, 19, 13, 09	-1986.06	47	135	4066.113	4117.975
E:\Beringia	E:\Beringia	02, 15, 05, 14, 16, 09	-1987.98	48	135	4071.956	4126.654
E:\Beringia	E:\Beringia	02, 15, 08, 14, 16, 09	-1981.28	54	135	4070.566	4144.816
E:\Beringia	E:\Beringia	02, 15, 10, 14, 16, 09	-1967.48	47	135	4028.967	4080.829
E:\Beringia	E:\Beringia	02, 15, 05, 17, 16, 09	-1984.26	48	135	4064.516	4119.214
E:\Beringia	E:\Beringia	02, 15, 08, 17, 16, 09	-1983.51	58	135	4083.014	4173.067
E:\Beringia	E:\Beringia	02, 15, 10, 17, 16, 09	-1967.08	45	135	4024.156	4070.672
E:\Beringia	E:\Beringia	02, 15, 05, 19, 16, 09	-1992.32	65	135	4114.635	4238.983
E:\Beringia	E:\Beringia	02, 15, 08, 19, 16, 09	-1988.71	70	135	4117.42	4272.732
E:\Beringia	E:\Beringia	02, 15, 10, 19, 16, 09	-1984.08	55	135	4078.169	4156.144
E:\Beringia	E:\Beringia	02, 15, 05, 14, 18, 09	-1981.73	51	135	4065.462	4129.366
E:\Beringia	E:\Beringia	02, 15, 08, 14, 18, 09	-1990	52	135	4083.998	4151.218
E:\Beringia	E:\Beringia	02, 15, 10, 14, 18, 09	-1963.25	46	135	4018.509	4067.645
E:\Beringia	E:\Beringia	02, 15, 05, 17, 18, 09	-1984.68	48	135	4065.359	4120.057
E:\Beringia	E:\Beringia	02, 15, 08, 17, 18, 09	-1992.57	57	135	4099.15	4185.02
E:\Beringia	E:\Beringia	02, 15, 10, 17, 18, 09	-1965.37	50	135	4030.75	4091.464
E:\Beringia	E:\Beringia	02, 15, 05, 19, 18, 09	-1989.7	58	135	4095.403	4185.455
E:\Beringia	E:\Beringia	02, 15, 08, 19, 18, 09	-1993.3	73	135	4132.596	4309.711
E:\Beringia	E:\Beringia	02, 15, 10, 19, 18, 09	-1977.54	55	135	4065.081	4143.056
E:\Beringia	E:\Beringia	02, 15, 05, 14, 12, 09	-1992.04	50	135	4084.072	4144.787
E:\Beringia	E:\Beringia	02, 15, 08, 14, 12, 09	-1988.75	58	135	4093.51	4183.562
E:\Beringia	E:\Beringia	02, 15, 10, 14, 12, 09	-1970.53	51	135	4043.053	4106.957
E:\Beringia	E:\Beringia	02, 15, 05, 17, 12, 09	-1992.34	54	135	4092.677	4166.927

E:\Beringia	E:\Beringia	02, 15, 08, 17, 12, 09		-1990.46	53	135	4086.927	4157.593
E:\Beringia	E:\Beringia	02, 15, 10, 17, 12, 09		-1971.32	47	135	4036.643	4088.505
E:\Beringia	E:\Beringia	02, 15, 05, 19, 12, 09		-1990.5	71	135	4123.004	4285.29
E:\Beringia	E:\Beringia	02, 15, 08, 19, 12, 09		-1998.59	57	135	4111.185	4197.055
E:\Beringia	E:\Beringia	02, 15, 10, 19, 12, 09		-1977.45	53	135	4060.908	4131.574
E:\Beringia	E:\Beringia	02, 08, 11, 12, 15		-2027.29	58	135	4170.588	4260.641
E:\Beringia	E:\Beringia	02, 06, 10, 13, 18, 19		-1970.41	59	135	4058.825	4153.225
E:\Beringia	E:\Beringia	02, 15, 10,	0.001	-2075.45	250	135	x	x
E:\Beringia	E:\Beringia	02, 15, 10,	0.01	-1917.4	207	135	x	x
E:\Beringia	E:\Beringia	02, 15, 10,	0.1	-1895.03	137	135	x	x
E:\Beringia	E:\Beringia	02, 15, 10,	0.5	-1947.36	75	135	4044.723	4237.944
E:\Beringia	E:\Beringia	02, 15, 10,	2	-1990.14	25	135	4030.274	4042.201
E:\Beringia	E:\Beringia	02, 15, 10,	5	-2041.27	16	135	4114.547	4119.157
E:\Beringia	E:\Beringia	02, 15, 10,	0.001	-2029.88	229	135	x	x
E:\Beringia	E:\Beringia	02, 15, 10,	0.01	-1969.88	203	135	x	x
E:\Beringia	E:\Beringia	02, 15, 10,	0.1	-1913.21	139	135	x	x
E:\Beringia	E:\Beringia	02, 15, 10,	0.5	-1951.61	63	135	4029.224	4142.802
E:\Beringia	E:\Beringia	02, 15, 10,	2	-1995.03	22	135	4034.059	4043.095
E:\Beringia	E:\Beringia	02, 15, 10,	5	-2041.45	16	135	4114.906	4119.516

BIC score
4241.329
4319.077
4145.121
4191.87
4308.747
4188.205
4202.646
4325.824
4184.187
4189.247
4247.694
4166.18
4210.265
4239.036
4143.575
4217.89
4361.873
4221.819
4225.094
4290.838
4190.361
4232.564
4293.07
4168.079
4195.454
4345.087
4204.364
4210.156
4305.406
4180.264
4210.589
4300.005
4150.552
4241.204
4370.987
4218.148
4208.942
4261.146
4181.356
4196.785
4317.716
4184.406
4204.619
4303.007
4193.166
4216.255
4269.878
4119.638
4216.362
4239.941
4135.436

4251.701
4332.277
4217.929
4213.465
4293.106
4157.194
4222.539
4292.882
4165.851
4254.497
4325.587
4221.817
4226.206
4292.582
4171.697
4225.751
4289.464
4159.114
4265.854
4341.629
4212.331
4222.444
4290.249
4219.963
4223.611
4255.166
4164.351
4234.519
4301.599
4202.661
4211.41
4227.451
4165.515
4203.969
4251.52
4154.893
4303.478
4320.789
4237.959
4213.631
4235.072
4152.151
4204.813
4264.75
4176.013
4263.908
4344.681
4224.871
4229.336
4262.016
4191.222
4249.561

4240.906
4173.191
4329.279
4276.785
4214.887
4339.094
4230.236
x
x
x
4262.619
4102.906
4161.031
x
x
x
4212.257
4097.975
4161.391

Variables	Regularization	Log Likeliho	Parameters	Sample Size	AIC score	AICc score
02, 15, 05, 14, 13, 11	1	-1994.06	40	135	4068.112	4103.005
02, 15, 08, 14, 13, 11	1	-2020.13	52	135	4144.262	4211.481
02, 15, 10, 14, 13, 11	1	-1980.91	47	135	4055.828	4107.69
02, 15, 05, 17, 13, 11	1	-1992.97	45	135	4075.95	4122.467
02, 15, 08, 17, 13, 11	1	-2020.3	47	135	4134.601	4186.463
02, 15, 10, 17, 13, 11	1	-1972.72	48	135	4041.441	4096.139
02, 15, 05, 19, 13, 11	1	-1990.7	46	135	4073.409	4122.545
02, 15, 08, 19, 13, 11	1	-2024.85	55	135	4159.699	4237.674
02, 15, 10, 19, 13, 11	1	-1980.71	43	135	4047.422	4089.005
02, 15, 05, 14, 16, 11	1	-1989.52	53	135	4085.038	4155.705
02, 15, 08, 14, 16, 11	1	-2002.79	48	135	4101.588	4156.286
02, 15, 10, 14, 16, 11	1	-1966.46	42	135	4016.913	4056.174
02, 15, 05, 17, 16, 11	1	-1985.53	45	135	4061.055	4107.572
02, 15, 08, 17, 16, 11	1	-2020.03	44	135	4128.055	4172.055
02, 15, 10, 17, 16, 11	1	-1961.07	45	135	4012.148	4058.665
02, 15, 05, 19, 16, 11	1	-1990.07	47	135	4074.135	4125.997
02, 15, 08, 19, 16, 11	1	-2026.66	55	135	4163.314	4241.289
02, 15, 10, 19, 16, 11	1	-1979.5	47	135	4053.002	4104.864
02, 15, 05, 14, 18, 11	1	-1970.38	57	135	4054.757	4140.627
02, 15, 08, 14, 18, 11	1	-2003.33	53	135	4112.658	4183.324
02, 15, 10, 14, 18, 11	1	-1956.76	47	135	4007.513	4059.375
02, 15, 05, 17, 18, 11	1	-1971.93	56	135	4055.851	4137.697
02, 15, 08, 17, 18, 11	1	-2006.71	55	135	4123.414	4201.388
02, 15, 10, 17, 18, 11	1	-1958.21	55	135	4026.42	4104.395
02, 15, 05, 19, 18, 11	1	-1986.49	51	135	4074.979	4138.883
02, 15, 08, 19, 18, 11	1	-2017.52	66	135	4167.041	4297.1
02, 15, 10, 19, 18, 11	1	-1976.65	49	135	4051.308	4108.955
02, 15, 05, 14, 12, 11	1	-1990.83	57	135	4095.669	4181.54
02, 15, 08, 14, 12, 11	1	-2021.9	57	135	4157.799	4243.669
02, 15, 10, 14, 12, 11	1	-1972.93	51	135	4047.857	4111.76
02, 15, 05, 17, 12, 11	1	-1992.9	43	135	4071.806	4113.388
02, 15, 08, 17, 12, 11	1	-2019.02	47	135	4132.044	4183.906
02, 15, 10, 17, 12, 11	1	-1972.53	42	135	4029.07	4068.331
02, 15, 05, 19, 12, 11	1	-1994.51	60	135	4109.011	4207.93
02, 15, 08, 19, 12, 11	1	-2030.38	53	135	4166.766	4237.432
02, 15, 10, 19, 12, 11	1	-1976.38	55	135	4062.755	4140.73
02, 15, 05, 14, 13, 06	1	-1996.07	47	135	4086.137	4137.999
02, 15, 08, 14, 13, 06	1	-2011.99	40	135	4103.973	4138.867
02, 15, 10, 14, 13, 06	1	-1977.83	50	135	4055.661	4116.375
02, 15, 05, 17, 13, 06	1	-1993.75	46	135	4079.496	4128.632
02, 15, 08, 17, 13, 06	1	-2015.88	49	135	4129.757	4187.404
02, 15, 10, 17, 13, 06	1	-1970.17	43	135	4026.34	4067.922
02, 15, 05, 19, 13, 06	1	-1991.91	46	135	4075.828	4124.965
02, 15, 08, 19, 13, 06	1	-2013.42	57	135	4140.849	4226.72
02, 15, 10, 19, 13, 06	1	-1981.57	46	135	4055.141	4104.277
02, 15, 05, 14, 16, 06	1	-1986	51	135	4073.991	4137.895
02, 15, 08, 14, 16, 06	1	-2002.78	43	135	4091.563	4133.145
02, 15, 10, 14, 16, 06	1	-1971.05	49	135	4040.103	4097.75
02, 15, 05, 17, 16, 06	1	-1985.86	47	135	4065.728	4117.59
02, 15, 08, 17, 16, 06	1	-2004.19	43	135	4094.384	4135.967
02, 15, 10, 17, 16, 06	1	-1967.45	44	135	4022.906	4066.906

02, 15, 05, 19, 16, 06	1	-1992.8	50	135	4085.591	4146.305
02, 15, 08, 19, 16, 06	1	-2021.57	54	135	4151.145	4225.395
02, 15, 10, 19, 16, 06	1	-1980.59	53	135	4067.179	4137.846
02, 15, 05, 14, 18, 06	1	-1970.21	62	135	4064.423	4172.923
02, 15, 08, 14, 18, 06	1	-1999.51	51	135	4101.011	4164.915
02, 15, 10, 14, 18, 06	1	-1953.42	51	135	4008.836	4072.739
02, 15, 05, 17, 18, 06	1	-1970.07	58	135	4056.134	4146.186
02, 15, 08, 17, 18, 06	1	-2001.66	52	135	4107.316	4174.535
02, 15, 10, 17, 18, 06	1	-1955.18	49	135	4008.364	4066.011
02, 15, 05, 19, 18, 06	1	-1988.55	58	135	4093.094	4183.146
02, 15, 08, 19, 18, 06	1	-2017.82	64	135	4163.647	4282.504
02, 15, 10, 19, 18, 06	1	-1979.05	52	135	4062.11	4129.33
02, 15, 05, 14, 12, 06	1	-1995.67	50	135	4091.34	4152.054
02, 15, 08, 14, 12, 06	1	-2012.15	49	135	4122.293	4179.94
02, 15, 10, 14, 12, 06	1	-1974.97	49	135	4047.945	4105.592
02, 15, 05, 17, 12, 06	1	-1991.94	43	135	4069.886	4111.469
02, 15, 08, 17, 12, 06	1	-2014.65	51	135	4131.309	4195.212
02, 15, 10, 17, 12, 06	1	-1965.97	52	135	4035.943	4103.162
02, 15, 05, 19, 12, 06	1	-1998.79	58	135	4113.581	4203.633
02, 15, 08, 19, 12, 06	1	-2027.5	52	135	4159.001	4226.221
02, 15, 10, 19, 12, 06	1	-1980.94	56	135	4073.879	4155.725
02, 15, 05, 14, 13, 09	1	-1998.96	52	135	4101.914	4169.134
02, 15, 08, 14, 13, 09	1	-1986.98	52	135	4077.963	4145.183
02, 15, 10, 14, 13, 09	1	-1974.65	54	135	4057.303	4131.553
02, 15, 05, 17, 13, 09	1	-1996.34	56	135	4104.69	4186.536
02, 15, 08, 17, 13, 09	1	-1983.38	52	135	4070.757	4137.976
02, 15, 10, 17, 13, 09	1	-1977.61	46	135	4047.225	4096.361
02, 15, 05, 19, 13, 09	1	-1992.84	53	135	4091.683	4162.35
02, 15, 08, 19, 13, 09	1	-1988.49	58	135	4092.982	4183.035
02, 15, 10, 19, 13, 09	1	-1985.61	46	135	4063.213	4112.35
02, 15, 05, 14, 16, 09	1	-1988.89	54	135	4085.781	4160.031
02, 15, 08, 14, 16, 09	1	-1978.59	55	135	4067.176	4145.151
02, 15, 10, 14, 16, 09	1	-1966.7	47	135	4027.407	4079.27
02, 15, 05, 17, 16, 09	1	-1984.26	51	135	4070.511	4134.415
02, 15, 08, 17, 16, 09	1	-1982.44	61	135	4086.877	4190.493
02, 15, 10, 17, 16, 09	1	-1969.46	41	135	4020.915	4057.947
02, 15, 05, 19, 16, 09	1	-1994.6	63	135	4115.194	4228.771
02, 15, 08, 19, 16, 09	1	-1990.37	67	135	4114.746	4250.746
02, 15, 10, 19, 16, 09	1	-1984.44	54	135	4076.888	4151.138
02, 15, 05, 14, 18, 09	1	-1980.49	49	135	4058.982	4116.629
02, 15, 08, 14, 18, 09	1	-1992.43	54	135	4092.853	4167.103
02, 15, 10, 14, 18, 09	1	-1964.66	58	135	4045.322	4135.374
02, 15, 05, 17, 18, 09	1	-1983.75	54	135	4075.501	4149.751
02, 15, 08, 17, 18, 09	1	-1992.41	51	135	4086.819	4150.723
02, 15, 10, 17, 18, 09	1	-1963.68	53	135	4033.359	4104.026
02, 15, 05, 19, 18, 09	1	-1989.23	61	135	4100.47	4204.086
02, 15, 08, 19, 18, 09	1	-1994.49	65	135	4118.981	4243.329
02, 15, 10, 19, 18, 09	1	-1978.06	56	135	4068.111	4149.957
02, 15, 05, 14, 12, 09	1	-1989.75	59	135	4097.495	4191.895
02, 15, 08, 14, 12, 09	1	-1990.98	53	135	4087.952	4158.619
02, 15, 10, 14, 12, 09	1	-1974.85	46	135	4041.708	4090.844
02, 15, 05, 17, 12, 09	1	-1991.88	58	135	4099.76	4189.813

02, 15, 08, 17, 12, 09	1	-1988.9	58	135	4093.805	4183.857
02, 15, 10, 17, 12, 09	1	-1967.09	53	135	4040.174	4110.841
02, 15, 05, 19, 12, 09	1	-1990.38	71	135	4122.753	4285.039
02, 15, 08, 19, 12, 09	1	-1995.44	62	135	4114.876	4223.376
02, 15, 10, 19, 12, 09	1	-1982.07	60	135	4084.135	4183.054
02, 08, 11, 12, 15	1	-2029.43	46	135	4150.866	4200.003
02, 06, 10, 13, 18, 19	1	-1970.89	56	135	4053.788	4135.634

BIC score
4184.323
4295.336
4192.376
4206.687
4271.149
4180.894
4207.051
4319.489
4172.349
4239.018
4241.041
4138.934
4191.792
4255.887
4142.886
4210.683
4323.104
4189.55
4220.357
4266.637
4144.061
4218.546
4283.204
4186.211
4223.148
4358.789
4193.666
4261.27
4323.4
4196.026
4196.732
4268.592
4151.092
4283.327
4320.745
4222.545
4222.684
4220.184
4200.924
4213.138
4272.116
4151.267
4209.471
4306.45
4188.783
4222.16
4216.49
4182.462
4202.276
4219.311
4150.738

4230.855
4308.03
4221.158
4244.55
4249.18
4157.005
4224.64
4258.39
4150.722
4261.599
4349.585
4213.184
4236.603
4264.652
4190.303
4194.813
4279.478
4187.017
4282.087
4310.075
4236.574
4252.988
4229.038
4214.187
4267.385
4221.831
4180.867
4245.663
4261.488
4196.856
4242.666
4226.966
4163.955
4218.68
4264.098
4140.031
4298.226
4309.399
4233.773
4201.34
4249.738
4213.828
4232.386
4234.988
4187.339
4277.692
4307.824
4230.806
4268.906
4241.932
4175.351
4268.266

4262.311
4194.154
4329.028
4295.003
4258.451
4284.509
4216.483

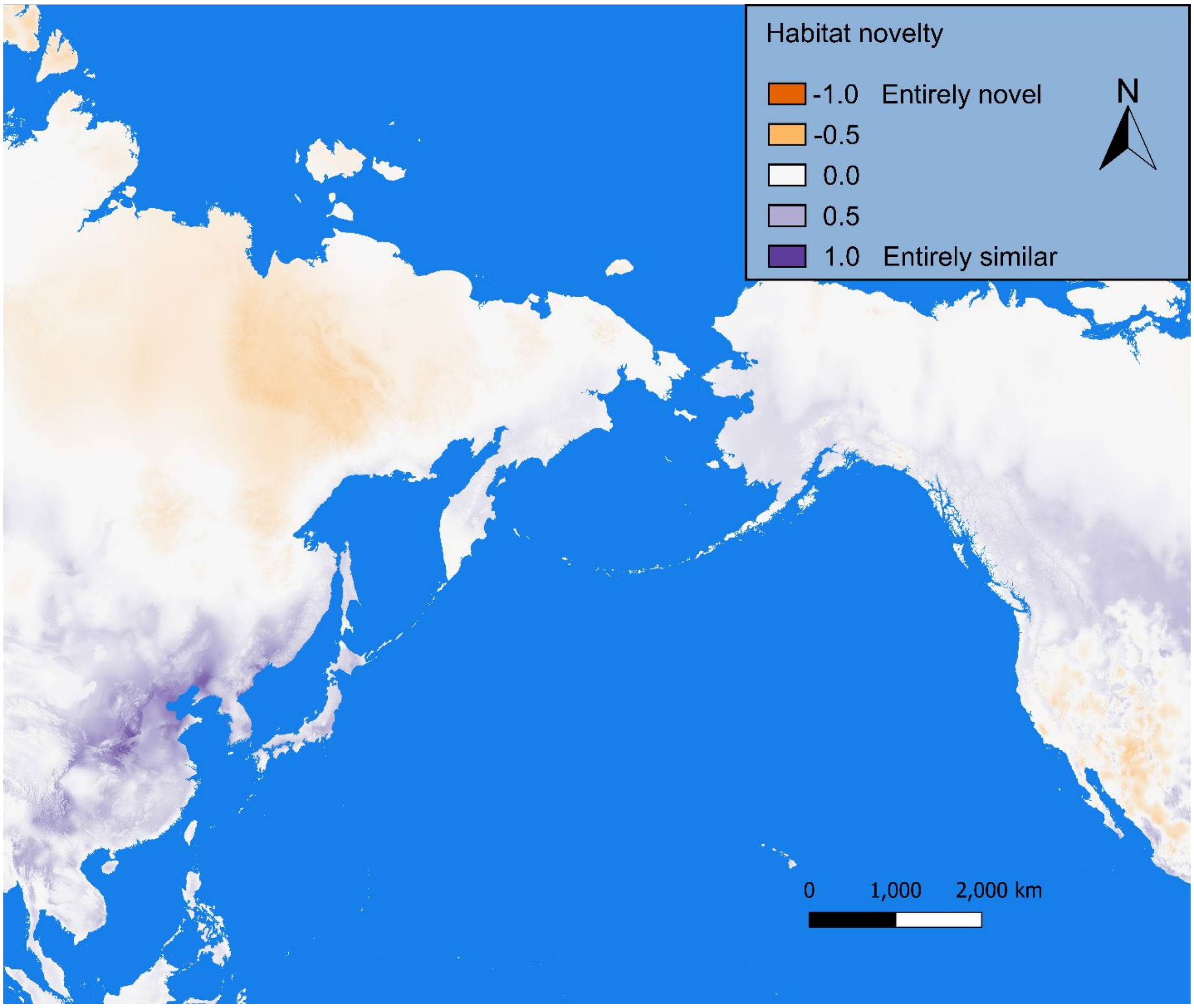

Supplementary Figure 1. A multidimensional environmental similarity surface (MESS) showing where novel climate conditions exist in the projection layers based on model 1 under current climate conditions.

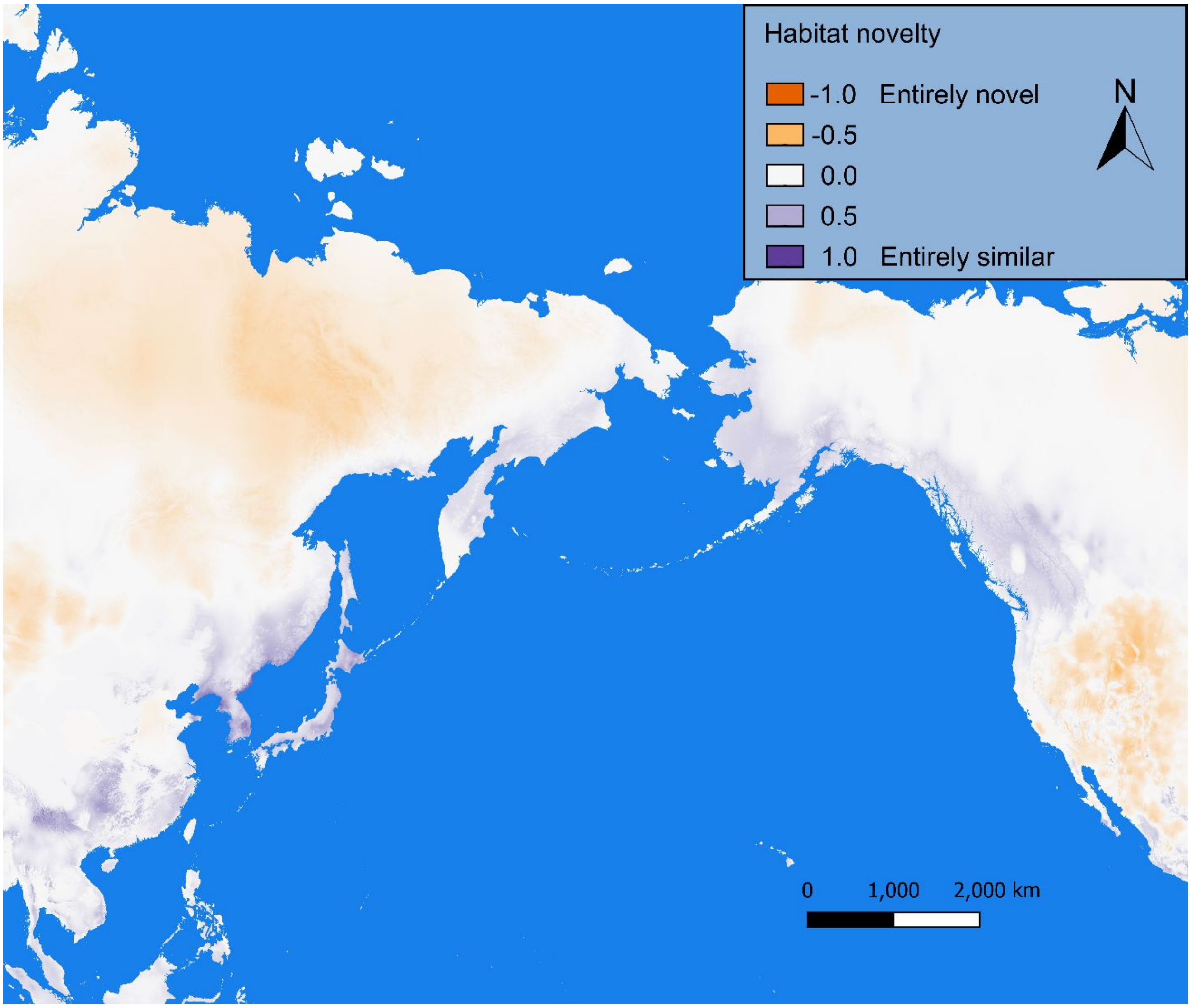

Supplementary Figure 2. A multidimensional environmental similarity surface (MESS) showing where novel climate conditions exist in the projection layers of the last interglacial period based on model 1.

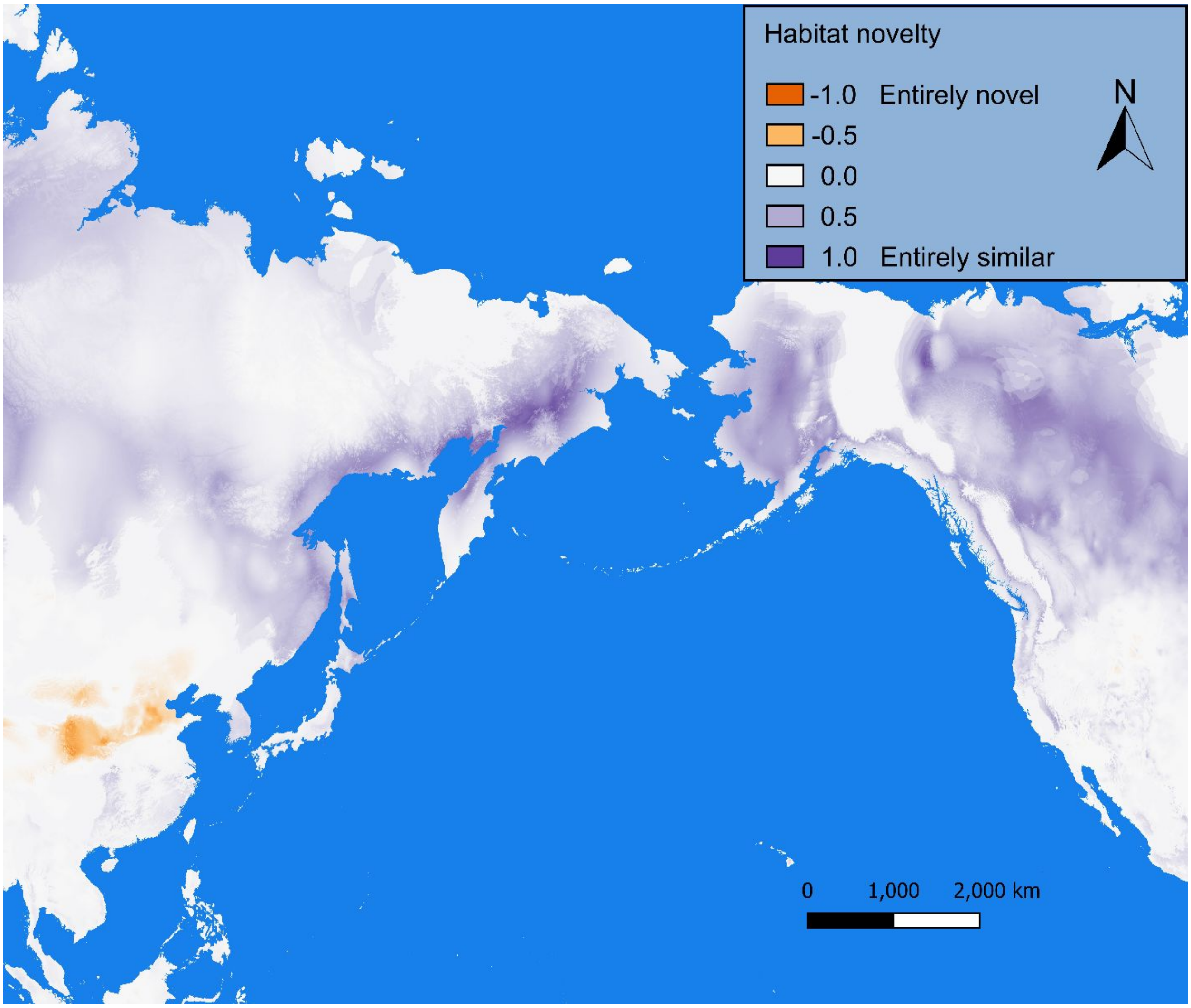

Supplementary Figure 3. A multidimensional environmental similarity surface (MESS) showing where novel climate conditions exist in the projection layers of the last interglacial period based on model 2.

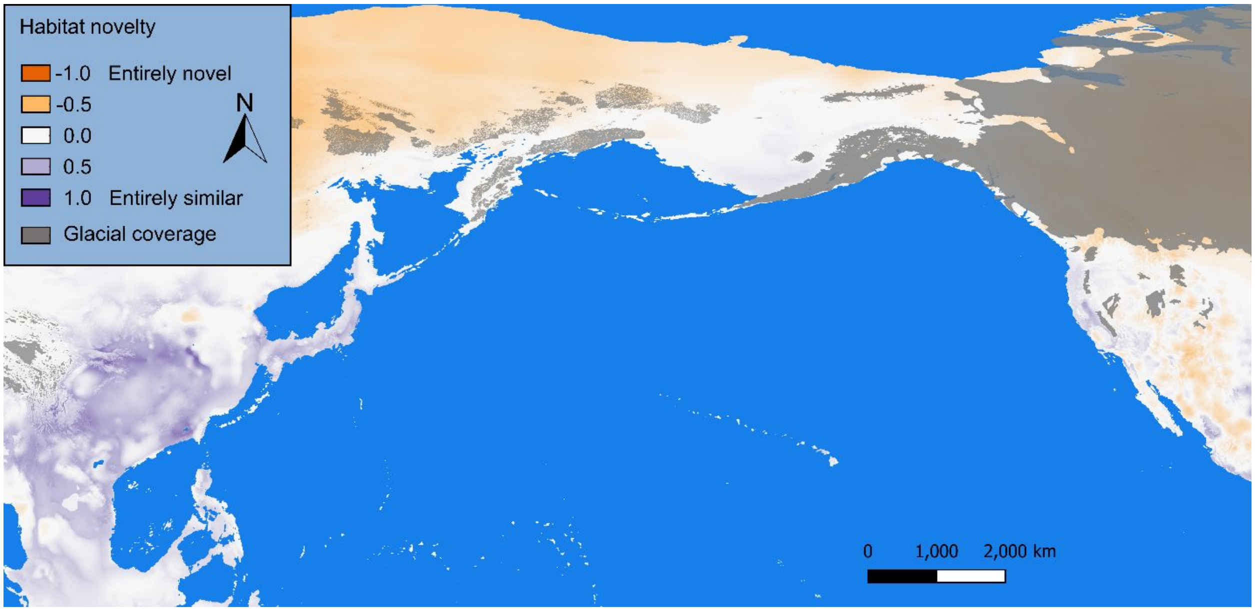

Supplementary Figure 4. A multidimensional environmental similarity surface (MESS) showing where novel climate conditions exist in the projection layers of the last glacial maximum based on model 1.

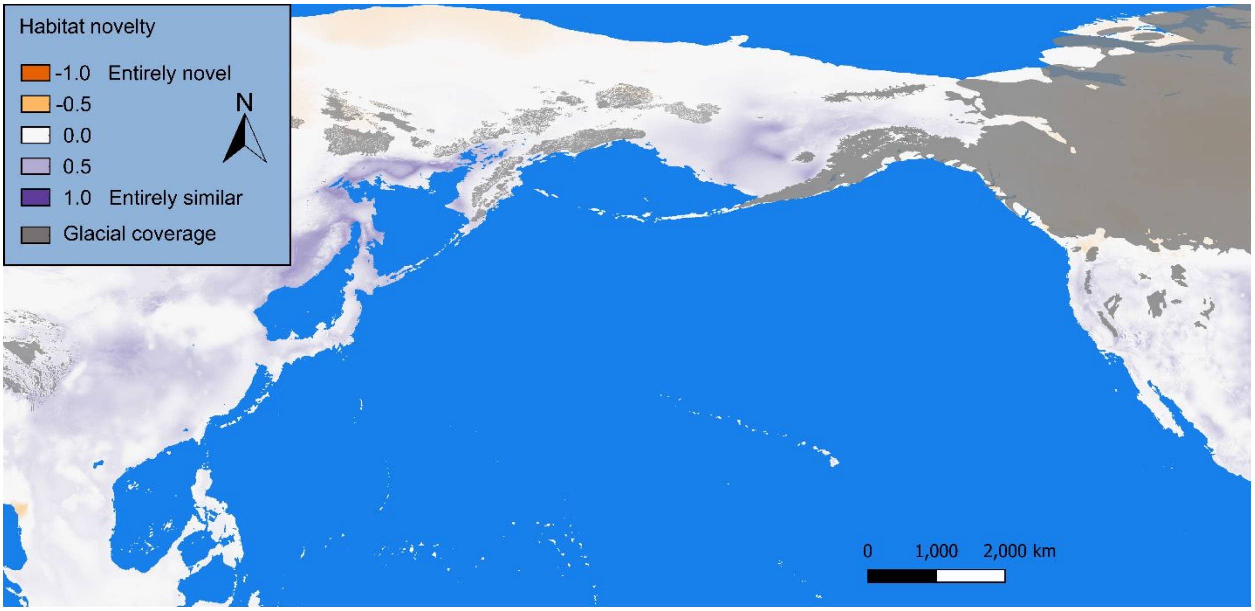

Supplementary Figure 5. A multidimensional environmental similarity surface (MESS) showing where novel climate conditions exist in the projection layers of the last glacial maximum based on model 2.

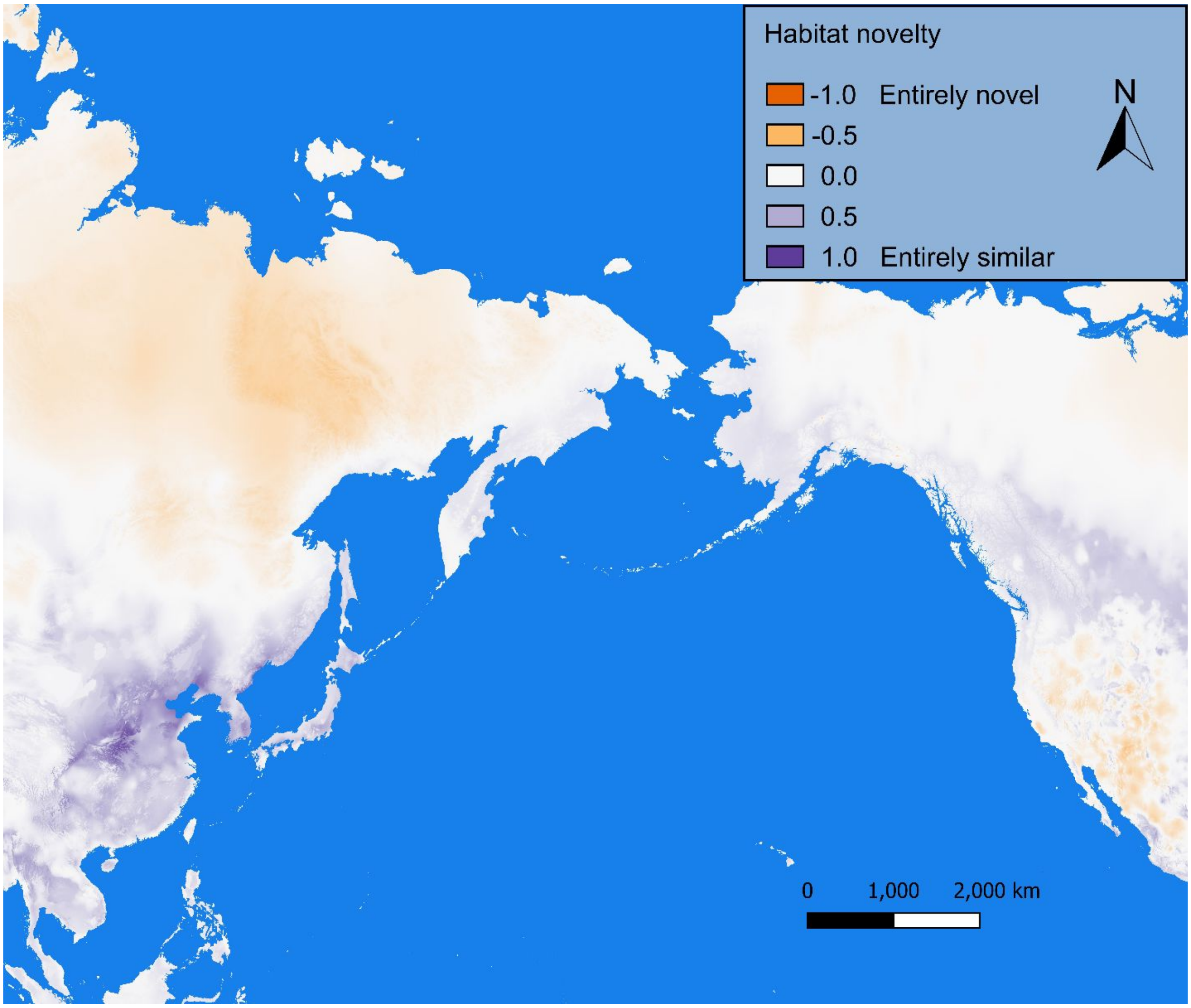

Supplementary Figure 5. A multidimensional environmental similarity surface (MESS) showing where novel climate conditions exist in the projection layers of the mid-Holocene period based on model 1.

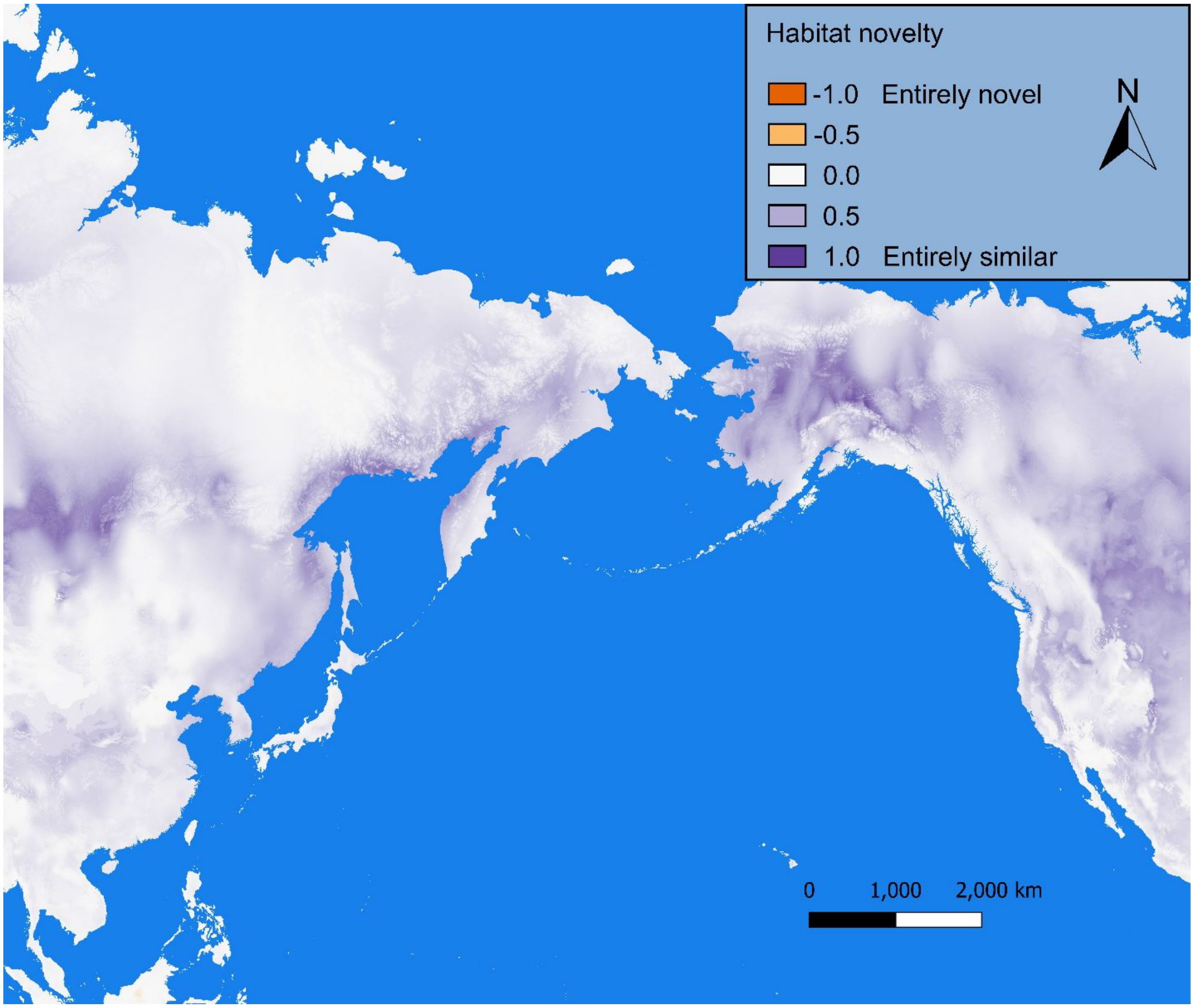

Supplementary Figure 7. A multidimensional environmental similarity surface (MESS) showing where novel climate conditions exist in the projection layers of the mid-Holocene period based on model 2.

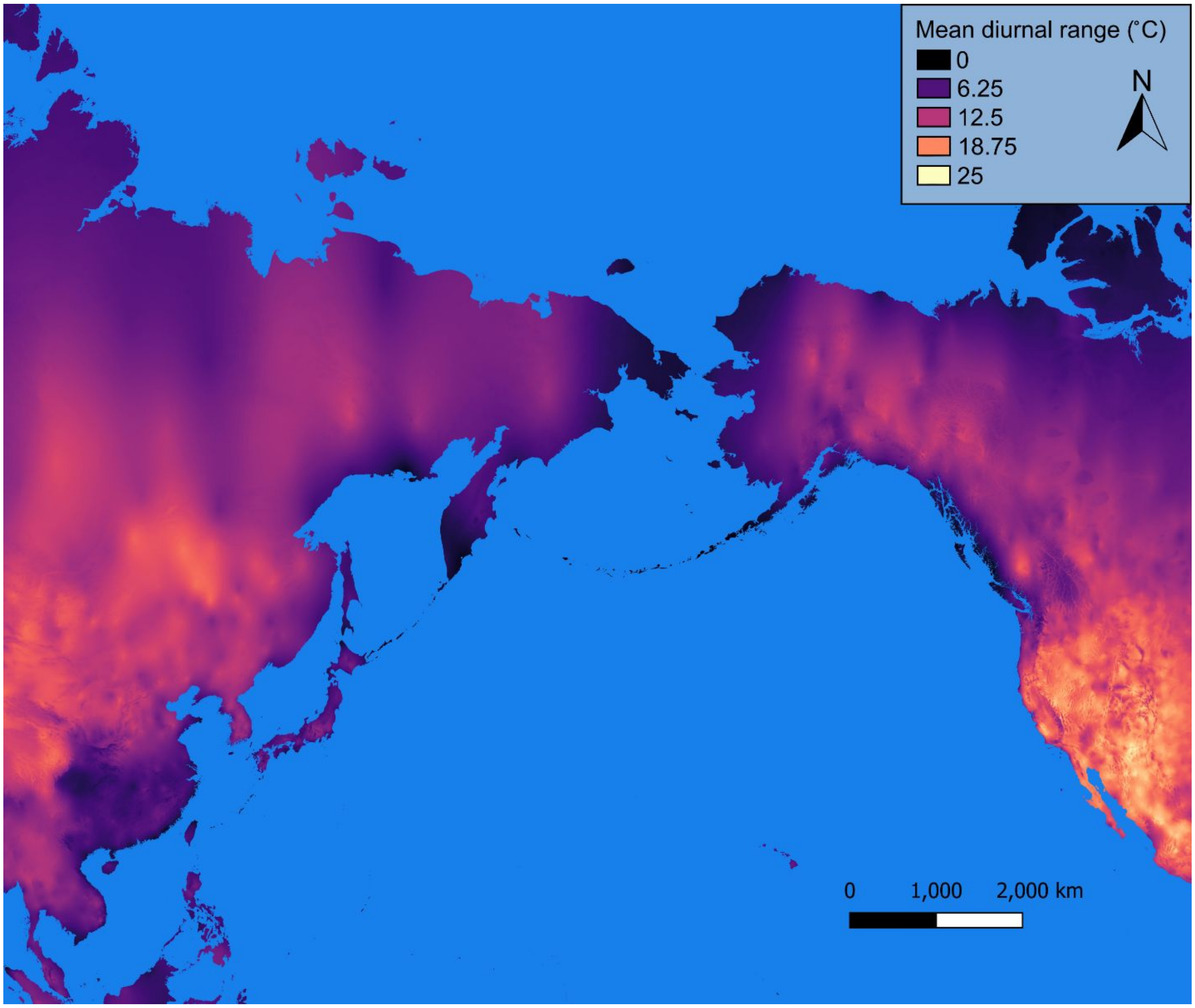

Supplementary Figure 8. Mean diurnal temperature range (BIO 02) under current climate conditions.

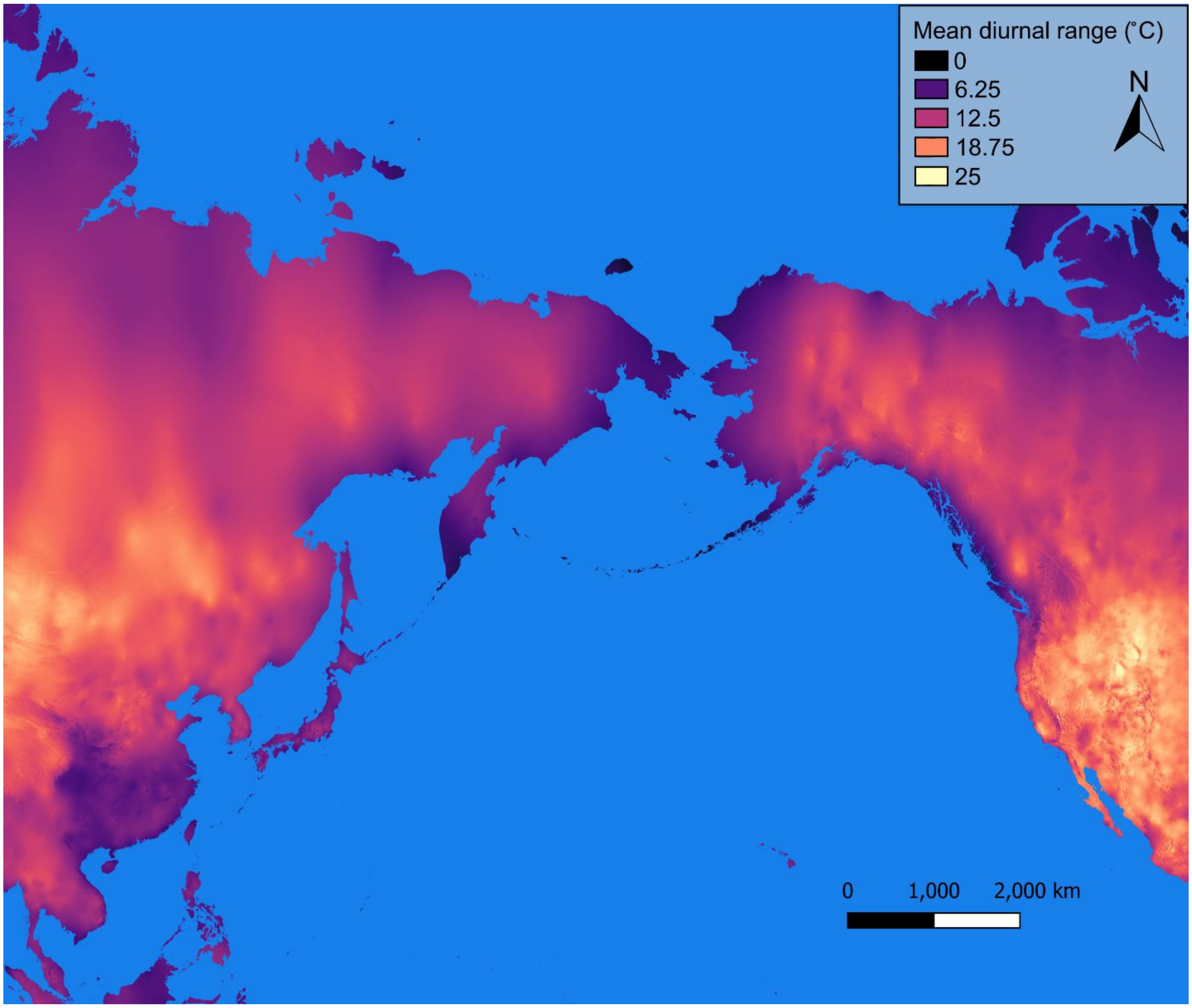

Supplementary Figure 9. Mean diurnal temperature range (BIO 02) during the last interglacial period.

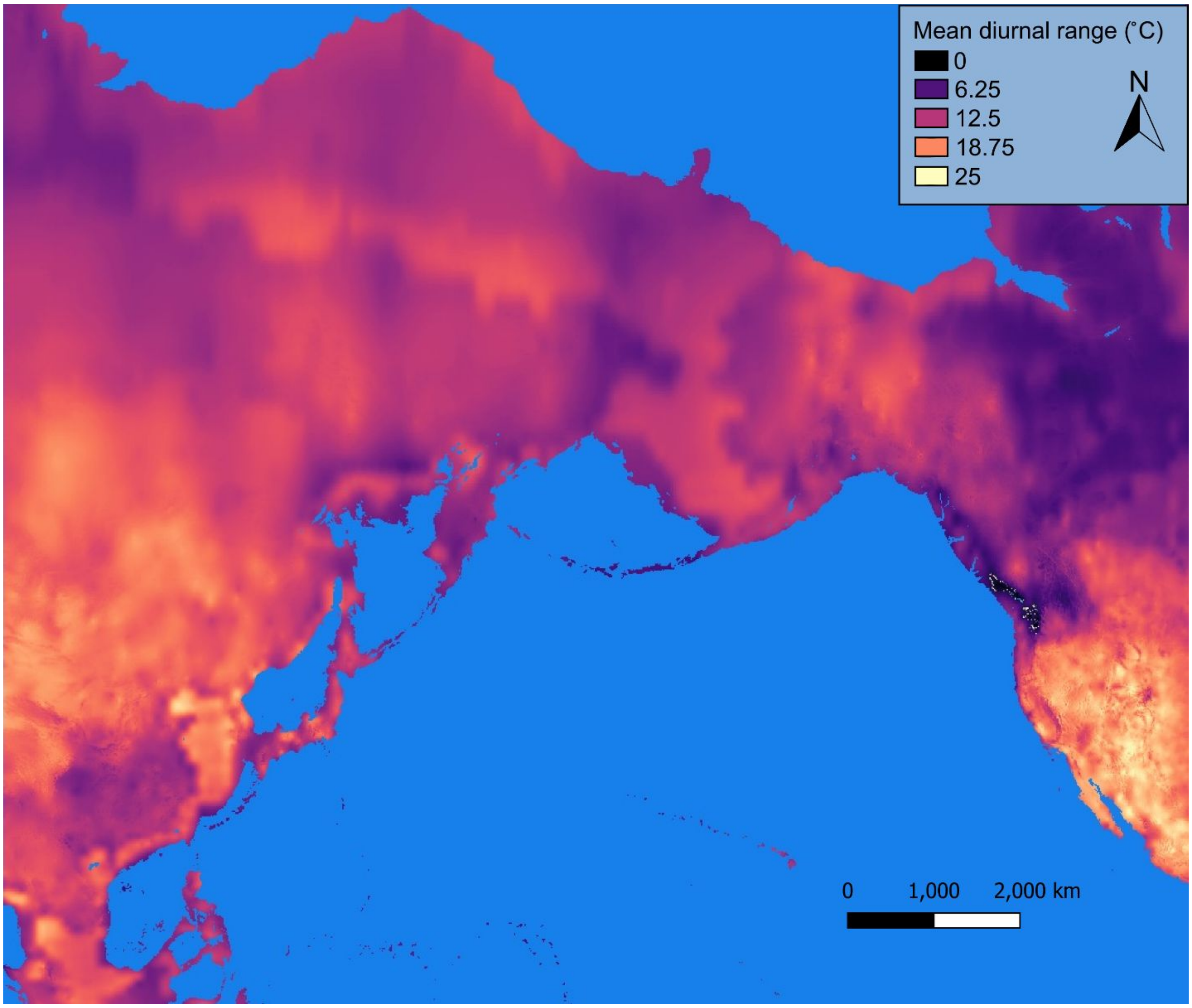

Supplementary Figure 10. Mean diurnal temperature range (BIO 02) during the last glacial maximum.

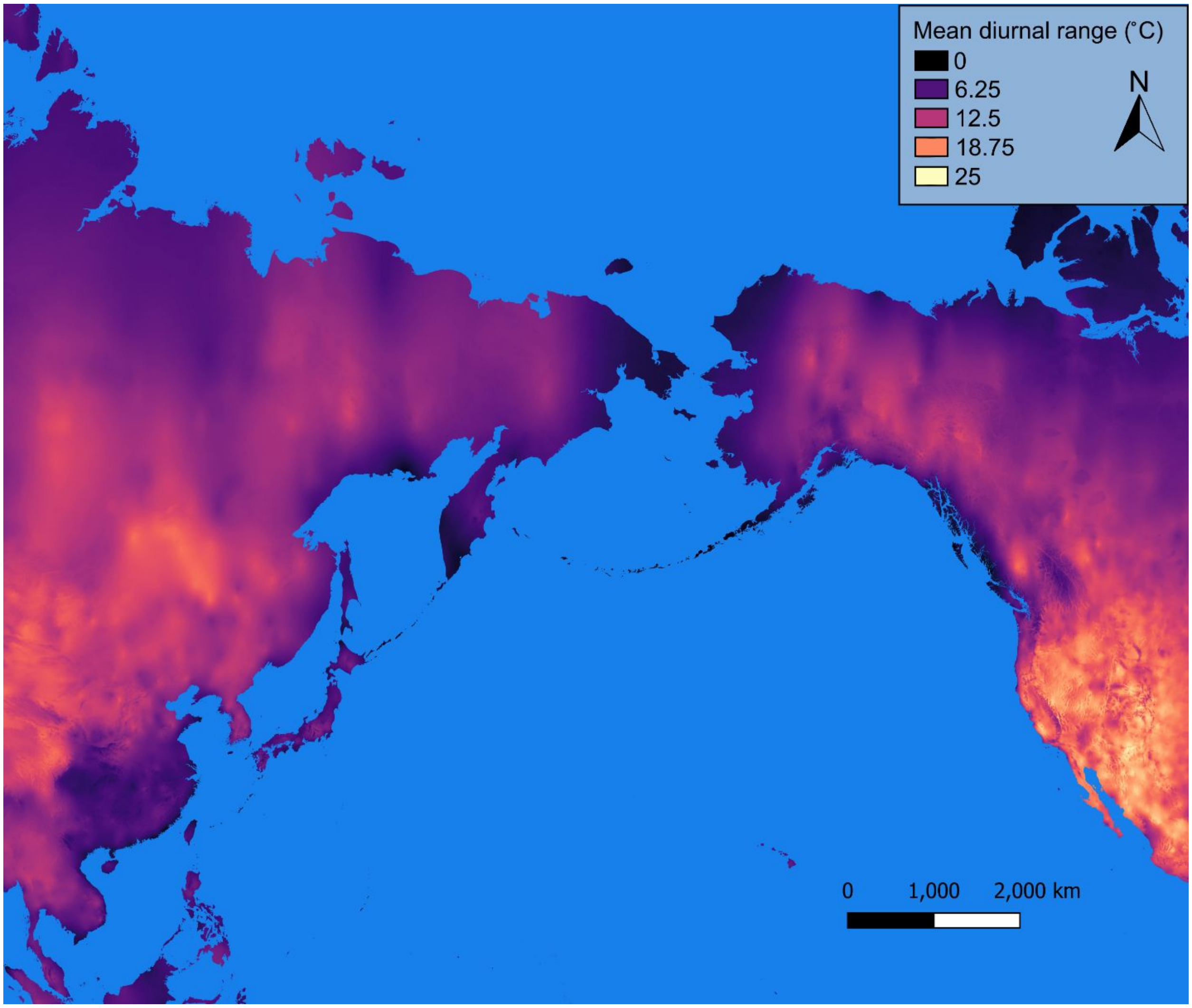

Supplementary Figure 11. Mean diurnal temperature range (BIO 02) during the mid-Holocene period.

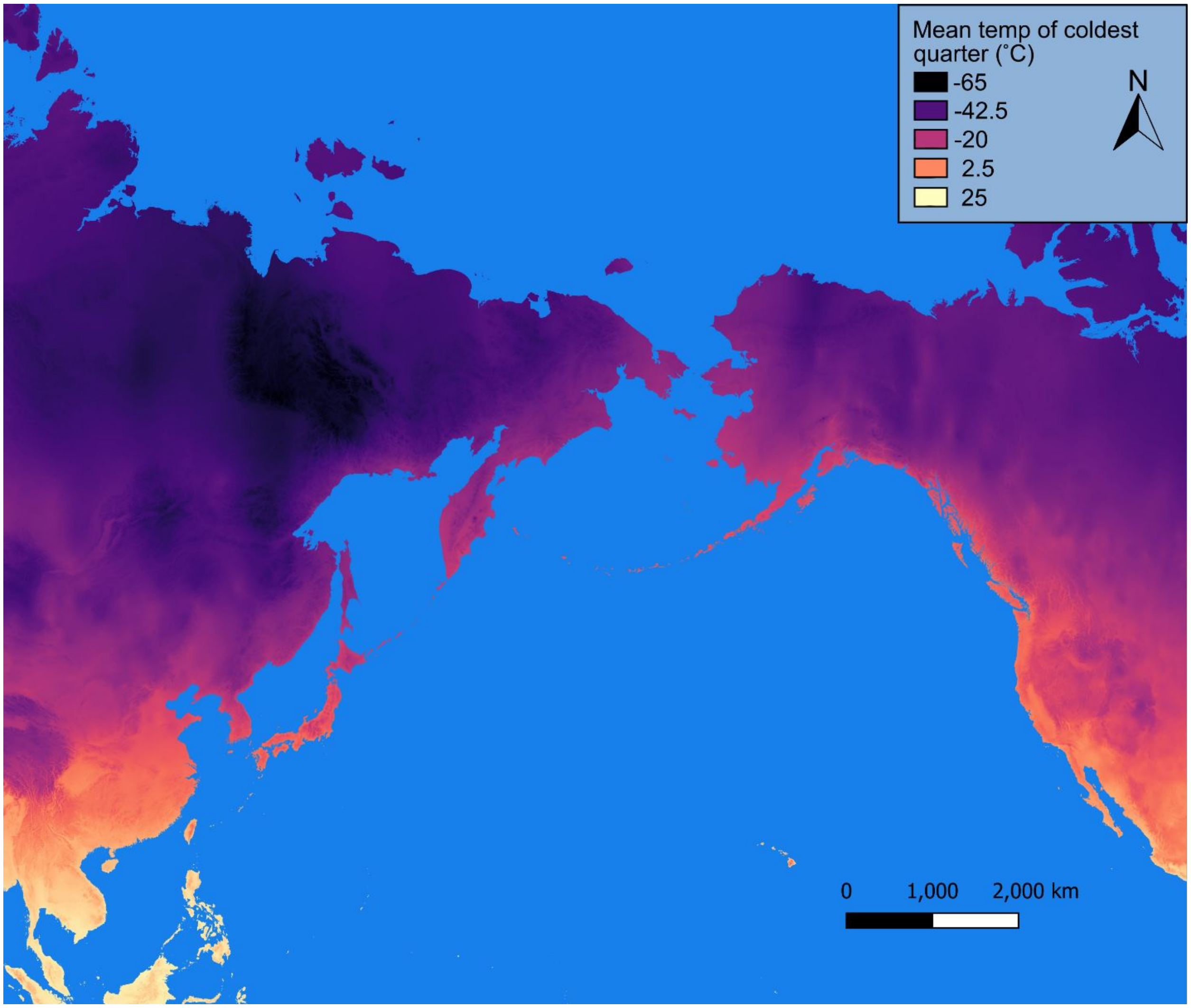

Supplementary Figure 12. Mean temperature of the coldest quarter (BIO 06) under current climate conditions.

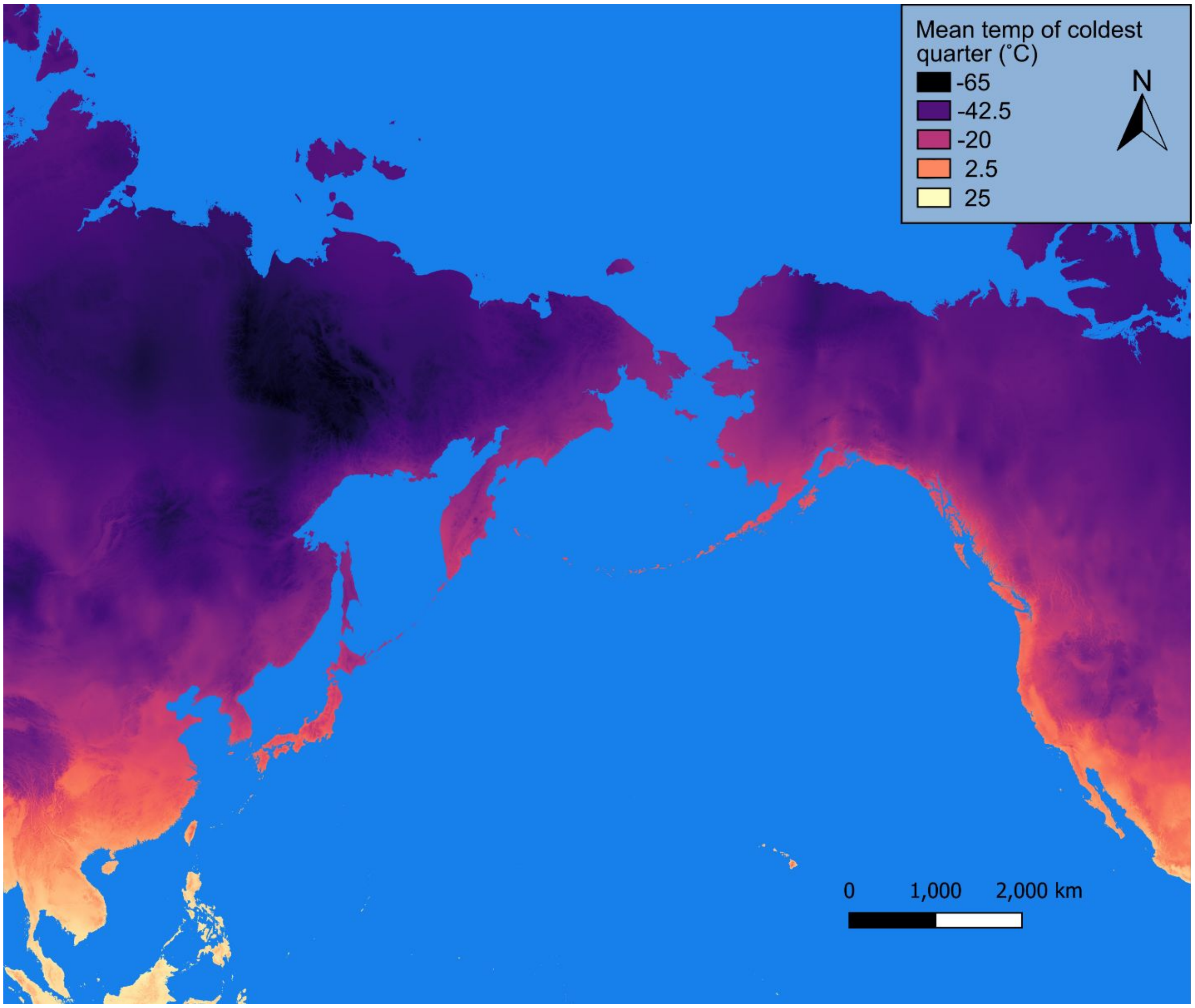

Supplementary Figure 13. Mean temperature of the coldest quarter (BIO 06) during the last interglacial period.

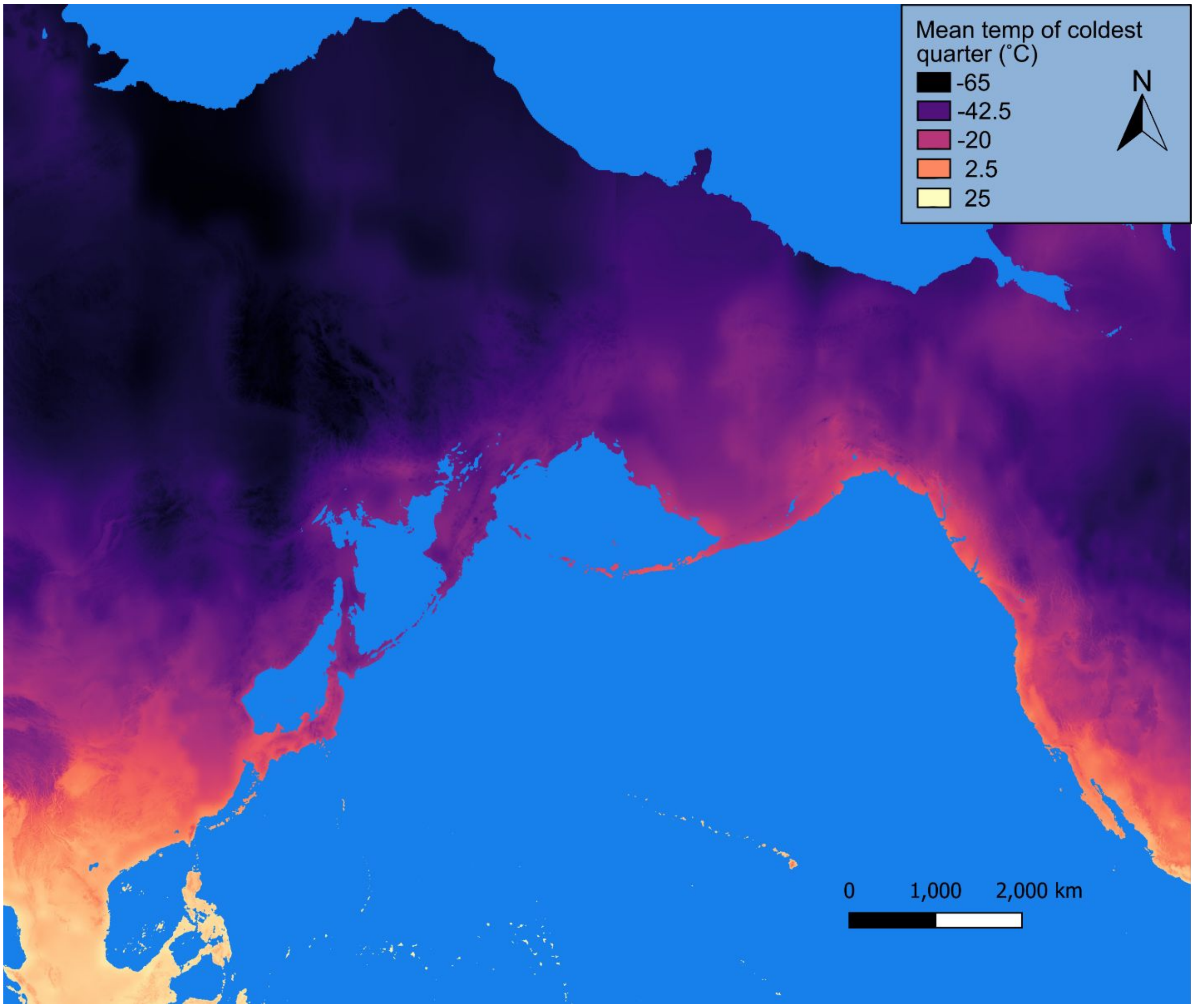

Supplementary Figure 14. Mean temperature of the coldest quarter (BIO 06) during the last glacial maximum.

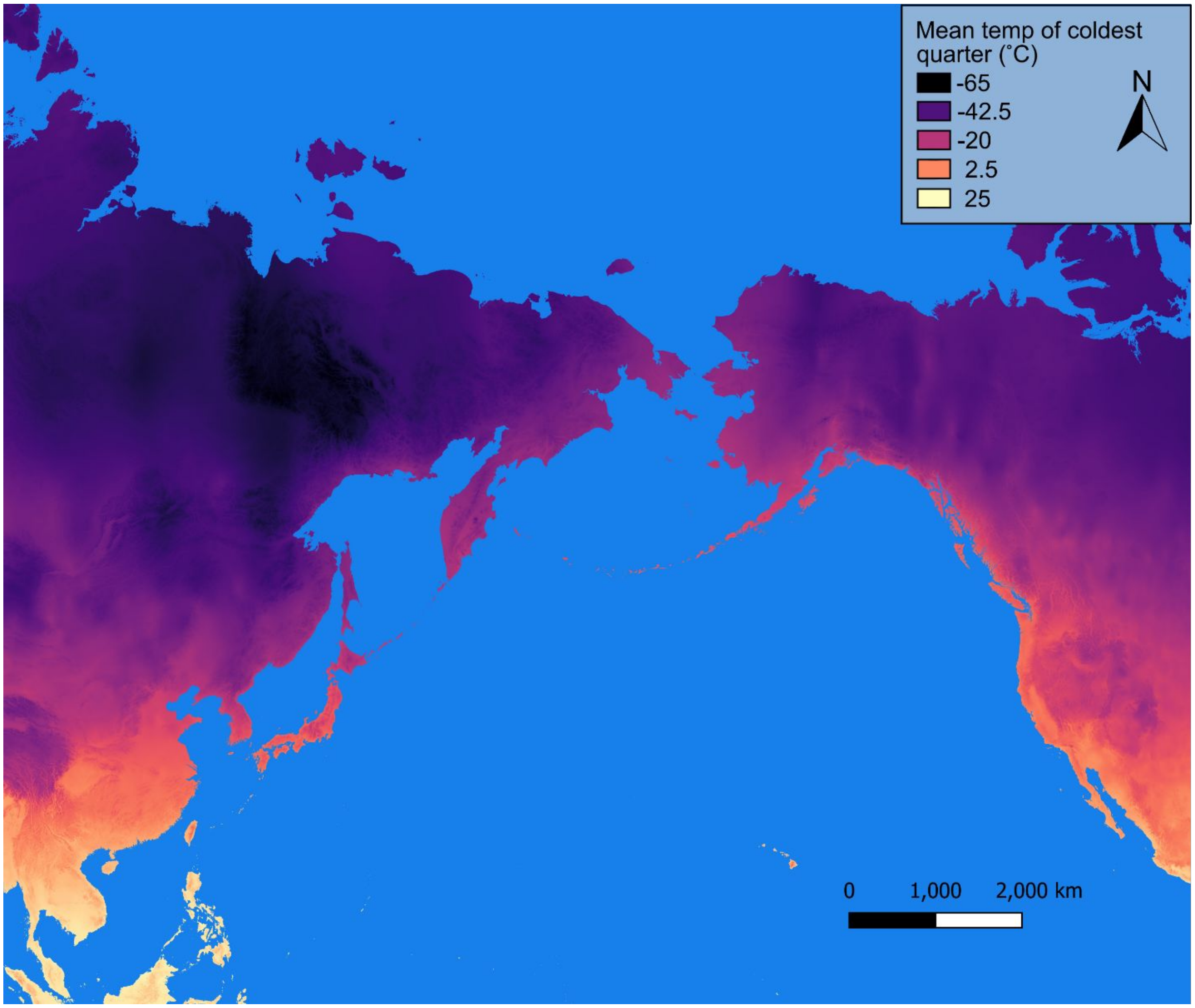

Supplementary Figure 15. Mean temperature of the coldest quarter (BIO 06) during the last mid-Holocene period.
