# Supplementary material for: Modeling the Putative Ancient Distribution of Aedes togoi (Diptera: Culicidae)
Source: J Insect Sci. 2020 May 26;20(3):7. doi: 10.1093/jisesa/ieaa035 (PMC7248266; doi:10.1093/jisesa/ieaa035)
Supplement: ieaa035_suppl_Supplementary_Files [file Peach decision letter.pdf › Peach decision letter.pdf]

Journal of Insect Science

Decision Letter (JIS-2020-0009)

From: konrad.fiedler@univie.ac.at

To: dan@danpeach.net

CC: konrad.fiedler@univie.ac.at

BCC:

Subject: Journal of Insect Science - Decision on Manuscript ID JIS-2020-0009

Body: 30-Jan-2020

Dear Mr. Peach,

Manuscript ID JIS-2020-0009 entitled "Modelling the Putative Ancient Distribution of the Costal Rock Pool Mosquito <i>Aedes togoi</i>" which you submitted to the Journal of Insect Science, has been reviewed and will be reconsidered for publication after the completion of the major revisions as noted. The comments of the reviewer(s) are included at the bottom of this letter.

Your revised manuscript should be uploaded within 45 days. Please let us know if you will require an extension. The due date is 15-Mar-2020.

To revise your manuscript, log into https://mc.manuscriptcentral.com/jis and enter your Author Centre, where you will find your manuscript title listed under "Manuscripts with Decisions." Under "Actions," click on "Create a Revision." Your manuscript number has been appended to denote a revision.

You may also click the below link to start the revision process.

*** PLEASE NOTE: This is a two-step process. After clicking on the link, you will be directed to a webpage to confirm. ***

https://mc.manuscriptcentral.com/jis?URL_MASK=7027ae3d67984000a9061f2c3f690395

Manuscript revision instructions
• Please ensure your paper follows journal style and requirements as listed in the instructions to authors: https://academic.oup.com/jinsectscience/pages/jinsectscience/pages/Instructions_To_Authors
• Please highlight the changes to your manuscript within the document by using the track changes mode in MS Word or by using bold or colored text. Please also include a point-by-point response to reviews as well as a clean version (non-track changes version) of the paper.
• If your paper was created in LaTeX, please upload the PDF along with the tex files.
• Please delete any outdated files (e.g., the original manuscript, any outdated figures, etc.) before uploading the revised files.
• Figures should be submitted as separate files and not inserted into the main document. Figures should be submitted as at least 300 dpi (600 dpi for line graphs) and in one of the following file types: tif, eps, rtf, ppt/pptx, xls/xlsx, editable PDF, ps, psd, ai, gif, or png.

Once again, thank you for submitting your manuscript to the Journal of Insect Science and I look forward to receiving your revision.

Sincerely,
Dr. Konrad Fiedler
Editor, Journal of Insect Science
konrad.fiedler@univie.ac.at
ESA Editorial Office: 3 Park Place, Suite 307, Annapolis, MD 21401-3722, USA. Editorial Office Phone: 1-301-731-4535.

Reviewer(s)' Comments to Author:

Reviewer: 1

Comments to the Author
The manuscript reports an ecological niche modelling for a vector competent mosquito species Aedes togoi

in the context of the question whether a Beringian dispersal could explain the two currently known distribution sites in Asia and North America.
I really enjoyed reading the manuscript. This paper provides an interesting analysis. The authors addressed an interesting question with adequate methodological approaches.
However, there are some items that should be addressed:
For the approach used here (an ecological niche modelling approach used for hindcasting, i.e. for projecting suitable habitats under past climatic conditions) it is of great importance to account for model extrapolations. Could you indicate areas with climatic conditions outside the range of climatic conditions considered in model training? In these areas, the modelling results are particularly subjected to uncertainties. Without the identification of those areas with climatic conditions outside the training range, the results cannot be interpreted in a reliable way.
Using ecological niche modelling approaches to project past climatic suitability requires certain assumptions that have to be satisfied. As for any ecological niche model, the species should be in equilibrium with its environment (no dispersal limitation), the occurrence data should be unbiased and reflect the potential distribution well, all relevant factors should be taken into account, and particularly important for hindcasting: the species' niche should be conserved over time. The aspect of niche conservatism is very important for the approach used for this study and still missing in the discussion.
I think it would be of interest to compare the respective niches of the species: in Asia, North America and based on all available records. Either considering the densities in the niche space as it is done e.g. in the R-package ecospat (cf. Broennimann et al. 2012) or at least comparing the modelled Maxent response curves for both models (cf. Figure 2, but it is hard to compare the curves due to the different axis scaling).
Model 1 uses the Asian data for training. Model 2 uses all available records (cf. L 152, table 2) and the whole North Pacific area as background (L.130)? Comparing Figure 3 and 4 it seems that model 2 results in generally lower modelled habitat suitability although more occurrence records certainly referring to a broader range of climatic conditions. One would perhaps expect otherwise. Here too, a comparison of the modelled niches would be informative. The discussion on this point should be extended. I am not yet convinced by the arguments (L.242). I'm not quite sure what influence the choice of background (and thus the prevalence) has on the maxent cloglog output. I could also imagine that the generally higher habitat suitability in model 1 is also due to methodological reasons. Have you looked at the thresholds according to different optimality criteria? Maybe binary habitat suitability maps would be easier to interpret? Binary modelling results would also have the advantage that they could be displayed together in one map for different time periods (and thus areas with continuously suitable climate conditions).
It would be of interest to see maps of the climatic conditions (for selected variables, e.g. bio06 or bio10) for the considered time periods.

Minor items:
I think the material and method section describing the modelling approach could be extended and some items should be clarified.
L122: Why are the boundaries of the second study area not shown? And why in Fig. 3 and not in Fig. 1?
L 126: Why did you exclude linear and quadratic features?
20% of data points excluded from model training may be problematic regarding the small number of available records.
What about model convergence? Could you please check? The default maximum number of iterations might be too low to ensure convergence.
L.134: How do you deal with the different spatial resolutions of the climatic data (current data used for training vs. past climate data)?
L136: Why didn't you use the most recent version of the worldclim data (current conditions)?
L168: Although it is often done in the literature, I think an interpretation of the exact AUC value is problematic, because the AUC value depends on the prevalence. Models with a low number of occurrences used for training tend to show higher AUC values. Thus, the AUC can only be used to compare different approaches based on the same occurrence data.
L272: the continuous presence of "suitable climatic conditions for" Ae. togoi
The comparison of single models of different explaining variable set and of different settings (table 2) is perhaps a bit too extensive. I would focus on the comparison between model 1 and model 2. (I would have preferred both models to be based on the same variables, even if they would then be slightly worse according to statistical criteria, because the modeled niche functions would then be more comparable)
Figure 2: Are these the so-called marginal response curves or response curves of the corresponding one variable maxent models?
All Figures: a scale bar would be preferable. Could you please specify the used projection / reference coordinate system?
Figure 3: Model 1, trained on the Asian data, projected to a larger extent. Could you please account for potential extrapolation and specify area outside the range of climatic conditions considered in model training? This is especially important for the projections on other time periods (modeling results under past climatic conditions) for both models.

Figure 7 and 8: with glacial coverage displayed as e.g. hatched area it would be easier to see the modelled climatic suitability in these areas.
References: Please check that all species names are written in italics

Date Sent: 30-Jan-2020

Print Close Window

© Clarivate Analytics | © ScholarOne, Inc., 2020. All Rights Reserved.

Admin: configure instructions |
