# Supplementary material for: Modeling the Putative Ancient Distribution of Aedes togoi (Diptera: Culicidae)
Source: J Insect Sci. 2020 May 26;20(3):7. doi: 10.1093/jisesa/ieaa035 (PMC7248266; doi:10.1093/jisesa/ieaa035)
Supplement: ieaa035_suppl_Supplementary_Files [file ieaa035_suppl_supplementary_files.zip › Peach response to reviews.docx]

We really appreciate the thorough and thoughtful comments of Reviewer 1 and, if they so choose, would be open to discussing future experiments in more detail outside of the context of this review. Please find below a detailed point-by-point response to their concerns and comments, as well as highlights in the main manuscript indicating changes.

Reviewer(s)' Comments to Author:

Reviewer: 1

Comments to the Author
1)	The manuscript reports an ecological niche modelling for a vector competent mosquito species Aedes togoi in the context of the question whether a Beringian dispersal could explain the two currently known distribution sites in Asia and North America.
I really enjoyed reading the manuscript. This paper provides an interesting analysis. The authors addressed an interesting question with adequate methodological approaches.

R1)	Thank you!

2)	However, there are some items that should be addressed:
For the approach used here (an ecological niche modelling approach used for hindcasting, i.e. for projecting suitable habitats under past climatic conditions) it is of great importance to account for model extrapolations. Could you indicate areas with climatic conditions outside the range of climatic conditions considered in model training? In these areas, the modelling results are particularly subjected to uncertainties. Without the identification of those areas with climatic conditions outside the training range, the results cannot be interpreted in a reliable way.

R2)	We thank the reviewer for bringing this to our attention. We have now included Supplementary Figures 1-7 that present a multidimensional environmental similarity surface (MESS) analysis, showing where novel climate conditions exist in the projected and hindcasted layers. We direct the reader towards these data in the results (L198-199).

3)	Using ecological niche modelling approaches to project past climatic suitability requires certain assumptions that have to be satisfied. As for any ecological niche model, the species should be in equilibrium with its environment (no dispersal limitation), the occurrence data should be unbiased and reflect the potential distribution well, all relevant factors should be taken into account, and particularly important for hindcasting: the species’ niche should be conserved over time. The aspect of niche conservatism is very important for the approach used for this study and still missing in the discussion.
I think it would be of interest to compare the respective niches of the species: in Asia, North America and based on all available records. Either considering the densities in the niche space as it is done e.g. in the R-package ecospat (cf. Broennimann et al. 2012) or at least comparing the modelled Maxent response curves for both models (cf. Figure 2, but it is hard to compare the curves due to the different axis scaling).

R3)	We have changed the axis scaling in Figure 2 to allow comparison between maxent response curves and added further discussion about the differences and similarities between the marginal response curves and niche conservatism (L317-327).

4)	Model 1 uses the Asian data for training. Model 2 uses all available records (cf. L 152, table 2) and the whole North Pacific area as background (L.130)? Comparing Figure 3 and 4 it seems that model 2 results in generally lower modelled habitat suitability although more occurrence records certainly referring to a broader range of climatic conditions. One would perhaps expect otherwise. Here too, a comparison of the modelled niches would be informative. The discussion on this point should be extended. I am not yet convinced by the arguments (L.242). I'm not quite sure what influence the choice of background (and thus the prevalence) has on the maxent cloglog output. I could also imagine that the generally higher habitat suitability in model 1 is also due to methodological reasons. Have you looked at the thresholds according to different optimality criteria? Maybe binary habitat suitability maps would be easier to interpret? Binary modelling results would also have the advantage that they could be displayed together in one map for different time periods (and thus areas with continuously suitable climate conditions).

R4)	We thank the reviewer for this suggestion. We have now used maximum test sensitivity plus specificity as a threshold to turn our maps into binary habitat suitability maps (L167-169, 191-193). We do not take a stand on the perspective mentioned in L242 (now L247-252), but we rather use this section to acknowledge its presence and we use both models for completeness, allowing the reader to see both. We have also added further clarification that the components of model 1 and model 2 are different, and differences in projected habitats should be expected (L247-248).

5)	It would be of interest to see maps of the climatic conditions (for selected variables, e.g. bio06 or bio10) for the considered time periods.

R5)	We agree that this is useful information for the readers and have added these to Supplementary Figures 8-15.

Minor items:

6)	I think the material and method section describing the modelling approach could be extended and some items should be clarified. L122: Why are the boundaries of the second study area not shown? And why in Fig. 3 and not in Fig. 1?

R6)	The boundaries of models 1 and 2 are now both shown on Figure 1. We thank the reviewer for the suggestion and agree that this presentation is much clearer!

7)	L 162: Why did you exclude linear and quadratic features?

R7)	We ran iterations both with and without each of these features to test which model’s fit scored best (as indicated by the best AICc - see Supplementary Tables 4 and 5). The text has been re-worded to increase the clarity of our methods (L163-164, 167-169) and we now refer to Supplementary Tables 4 and 5 in the relevant portion of the methods section (L151-152).

8)	20% of data points excluded from model training may be problematic regarding the small number of available records. What about model convergence? Could you please check? The default maximum number of iterations might be too low to ensure convergence.

R8)	We have re-run our models with 2000 iterations to identify when convergence occurred. Model 1 converged after a mean of 892.8 iterations and model 2 after a mean of 1073 iterations. We have added this information to the description of the models in the methods (L191-193).

9)	L.134: How do you deal with the different spatial resolutions of the climatic data (current data used for training vs. past climate data)?

R9)	Maxent automatically does this. It is the numerical value associated with each pixel that is compared, rather than its size. For example, the value of a 5km^2^ pixel is the average of the pixels it contains at greater resolution. This can lead to errors in interpreting the results if one is not conscious of this, but we have included a discussion of caveats related to spatial resolution (L252-255) to indicate that even at 1km^2^ it is almost certain that only subsets of these areas contain suitable habitat.

10)	L136: Why didn’t you use the most recent version of the worldclim data (current conditions)?

R10)	The past climate data are downscaled from Worldclim v1, and we wanted to maintain fidelity between our current and past climate models. Unfortunately, new versions of this past data based on Worldclim v2 are not yet available.

11)	L168: Although it is often done in the literature, I think an interpretation of the exact AUC value is problematic, because the AUC value depends on the prevalence. Models with a low number of occurrences used for training tend to show higher AUC values. Thus, the AUC can only be used to compare different approaches based on the same occurrence data.

R11)	We only use the AUC to compare different approaches based on the same occurrence data (e.g. to compare models based on occurrence data in Asia to other models based on the same). Original Table 2 has been separated into two tables (Table 2 and Table 3) to more clearly communicate that we did not intend for readers to directly compare between the models using the AUC.

12)	L272: the continuous presence of “suitable climatic conditions for” Ae. togoi

R12)	This has been changed as suggested (L279). Thanks!

13)	The comparison of single models of different explaining variable set and of different settings (table 2) is perhaps a bit too extensive. I would focus on the comparison between model 1 and model 2. (I would have preferred both models to be based on the same variables, even if they would then be slightly worse according to statistical criteria, because the modeled niche functions would then be more comparable)

R13)	We included Table 2 in order to be fully transparent about how we arrived at the variable sets that we did. We have also now separated each model into its own table (Table 2 and Table 3).

14)	Figure 2: Are these the so-called marginal response curves or response curves of the corresponding one variable maxent models?

R14)	These are the marginal response curves. We have added this information to the caption of Figure 2 to increase clarity.

15)	All Figures: a scale bar would be preferable. Could you please specify the used projection / reference coordinate system?

R15)	We have added that we used WGS84 Mercator for the last glacial maximum maps and WGS84/PDC Mercator projection for all other maps into the methods section (L131-132) and added scale bars to all maps.

16)	Figure 3: Model 1, trained on the Asian data, projected to a larger extent. Could you please account for potential extrapolation and specify area outside the range of climatic conditions considered in model training? This is especially important for the projections on other time periods (modeling results under past climatic conditions) for both models.

R16)	We have added this to the Supplementary Figures and the Results (please refer to response R3 for more details).

17)	Figure 7 and 8: with glacial coverage displayed as e.g. hatched area it would be easier to see the modelled climatic suitability in these areas.

R17)	We were unable to find a hatched area display that we were happy with to denote glacial coverage. Instead we darkened the transparent grey used to denote glacial coverage which, with the yellow in our revised colour scheme, greatly improves visibility.

18)	References: Please check that all species names are written in italics

R18)	There was an issue with formatting in our reference management software. This has now been corrected. Thanks!
